# Transcription Coactivators p300 and CBP Are Necessary for Photoreceptor-Specific Chromatin Organization and Gene Expression

**DOI:** 10.1371/journal.pone.0069721

**Published:** 2013-07-26

**Authors:** Anne K. Hennig, Guang-Hua Peng, Shiming Chen

**Affiliations:** 1 Department of Ophthalmology and Visual Sciences, Washington University School of Medicine, St. Louis, Missouri, United States of America; 2 Department of Developmental Biology, Washington University School of Medicine, St. Louis, Missouri, United States of America; University of Cologne, Germany

## Abstract

Rod and cone photoreceptor neurons in the mammalian retina possess specialized cellular architecture and functional features for converting light to a neuronal signal. Establishing and maintaining these characteristics requires appropriate expression of a specific set of genes, which is tightly regulated by a network of photoreceptor transcription factors centered on the cone-rod homeobox protein CRX. CRX recruits transcription coactivators p300 and CBP to acetylate promoter-bound histones and activate transcription of target genes. To further elucidate the role of these two coactivators, we conditionally knocked out *Ep300* and/or *CrebBP* in differentiating rods or cones, using *opsin*-driven *Cre recombinase*. Knockout of either factor alone exerted minimal effects, but loss of both factors severely disrupted target cell morphology and function: the unique nuclear chromatin organization seen in mouse rods was reversed, accompanied by redistribution of nuclear territories associated with repressive and active histone marks. Transcription of many genes including CRX targets was severely impaired, correlating with reduced histone H3/H4 acetylation (the products of p300/CBP) on target gene promoters. Interestingly, the presence of a single wild-type allele of either coactivator prevented many of these defects, with *Ep300* more effective than *Cbp*. These results suggest that p300 and CBP play essential roles in maintaining photoreceptor-specific structure, function and gene expression.

## Introduction

The mammalian retina consists of three layers of neurons specialized for light detection and initial processing of visual signals [1] [2]. Photoreceptors are located in the outer layer, and constitute 70% of retinal cells. These cells, which convert light to a neuronal signal, contain specific cellular structures including apical membrane specializations in the “outer segment” that capture light photons, ribbon-type synaptic specializations for transmitting neural signals to interneurons in the inner retinal layers, and a unique nuclear chromatin organization to mediate cell-type-specific gene expression while maximizing the amount of light reaching the outer segments. The vast majority of photoreceptors in most mammalian retinas are rods, which are exquisitely sensitive to low levels of light and mediate night vision. 3–5% of photoreceptors in mouse and human retinas are cones, which mediate color vision in daylight. Cones can be further classified on the basis of the wavelength sensitivity of the light-capturing visual pigment opsin they contain. To establish and maintain their structure and function, each photoreceptor subtype expresses a set of specific genes including the characteristic opsin, under the tight regulation of a network of photoreceptor-specific transcription factors [3] [4]. The central player, the cone-rod homeobox transcription factor CRX, interacts with photoreceptor subtype-specific transcription factors such as NRL and NR2E3 in rods or TRβ2 and RXRγ in cones, to activate or suppress expression of rod vs. cone gene sets. We demonstrated previously that CRX activates transcription by interacting with coactivators or coactivator complexes including CBP, P300, and GCN5 (KAT2A), a component of the STAGA chromatin remodeling complex [5] [8]. All of these coactivators contain intrinsic lysine acetyltransferase (KAT) activity, catalyzing acetylation of core histone tails and other proteins.

Acetylated histones are active marks for transcription, often associated with “open” chromatin that is accessible to the transcription machinery and transcription regulators [9] [10] [11] [12]. Histone acetylation is controlled by two classes of enzymes with opposing functions: Histone lysine (K) acetyltransferases (HATs) add the acetyl groups to specific lysine residues in the tails of core histones, and histone deacetylases (HDACs) remove them.

In mammals, there are four major families of HATs, whose members show high degrees of homology and, in some cases, functional redundancy (reviewed in [13] [14]). This is true for the well-studied KAT3 family members, “CREB Binding Protein” (CREBBP, CBP or KAT3A) [6] and the closely related “Adenovirus E1A-associated 300-kD Protein” (p300 or KAT3B) [7], which catalyze acetylation of all core histones, particularly H3 and H4 [15] [16] [17] [18]. In addition, CBP and p300 act as transcription coactivators by interacting with a variety of specific transcription factors and co-regulators [13] [19] to regulate the expression of numerous genes important in embryonic development [20], cell proliferation and differentiation [21] [22], neuronal function [23] [24] [25] [26] [27], energy homeostasis [20] [28] [29] [30], and tumor suppression [31] [32]. In humans, mutations in *CBP* or *EP300* are associated with Rubinstein-Taybi syndrome (RSTS) (http://omim.org/entry/180849, http://omim.org/entry/613684), an autosomal-dominant disorder characterized by mental and growth retardation and skeletal abnormalities [33] [34]. Molecular mechanism studies have found that RSTS-causing mutations affect acetyltransferase catalytic activity and coactivator function [35] [36]. The importance of HAT catalytic activity was further supported by the finding that p300/CBP-mediated acetylation of histone H3 lysine 18/27 recruits RNA polymerase II to target gene promoters in response to ligand-induced nuclear receptor activation [11]. In mice, p300 and CBP are required for embryonic development and viability. Conventional knockout of either factor in mice is early embryonic lethal [37] [38], complicating investigation of the roles of p300/CBP in fetal development of specific tissues such as the nervous system. This limitation has been circumvented by *Cre-loxP*-mediated conditional knockout strategies, leading to the findings that p300 and CBP play redundant and distinct functions in thymocyte and T-cell development [19] [39], and that p300/CBP in the brain is required for formation of long-term memories [26]. However, the role of p300/CBP in the retina (a part of the central nervous system) is not clear, although retinal dystrophy and glaucoma are commonly seen in RSTS patients [40].

Results of several studies suggest that p300/CBP HATs may play important roles in retinal photoreceptor development and maintenance. First, both coactivators are expressed by developing and mature photoreceptors, and physically interact with the key photoreceptor transcription factor CRX [5] [41] [42]. Second, during photoreceptor development, both factors are found on the promoter/enhancer regions of CRX-regulated photoreceptor genes after CRX binds. These events are followed by acetylation of histone H3 and H4 on these promoters, recruitment of additional photoreceptor-specific transcription factors, and transcriptional activation of the associated genes [5]. Increases in H3 acetylation have also been associated with activation by NRL [43]. Third, in the absence of CRX (*Crx^−/−^* mouse retina), recruitment of CBP to target gene promoters and acetylated histone H3/H4 levels are reduced, correlating with decreased transcription [5]. To examine the role of p300/CBP in CRX-regulated photoreceptor gene expression, we conditionally knocked out *Ep300 and/or Cbp* in rods or cones of the mouse retina using either a r*hodopsin* or *cone opsin* promoter to drive *Cre recombinase* expression. Here we report that loss of both p300 and CBP, but neither alone, causes detrimental defects in rod/cone structure and function, maintenance of photoreceptor gene expression and cell identity. These defects are accompanied by drastically reduced acetylation of histone H3/H4 on photoreceptor genes, and loss of the nuclear chromatin organization pattern characteristic of mouse photoreceptors [44].

## Results

### Generation of photoreceptor-specific *Ep300/Cbp* conditional knockout mice

**Figure 1:**
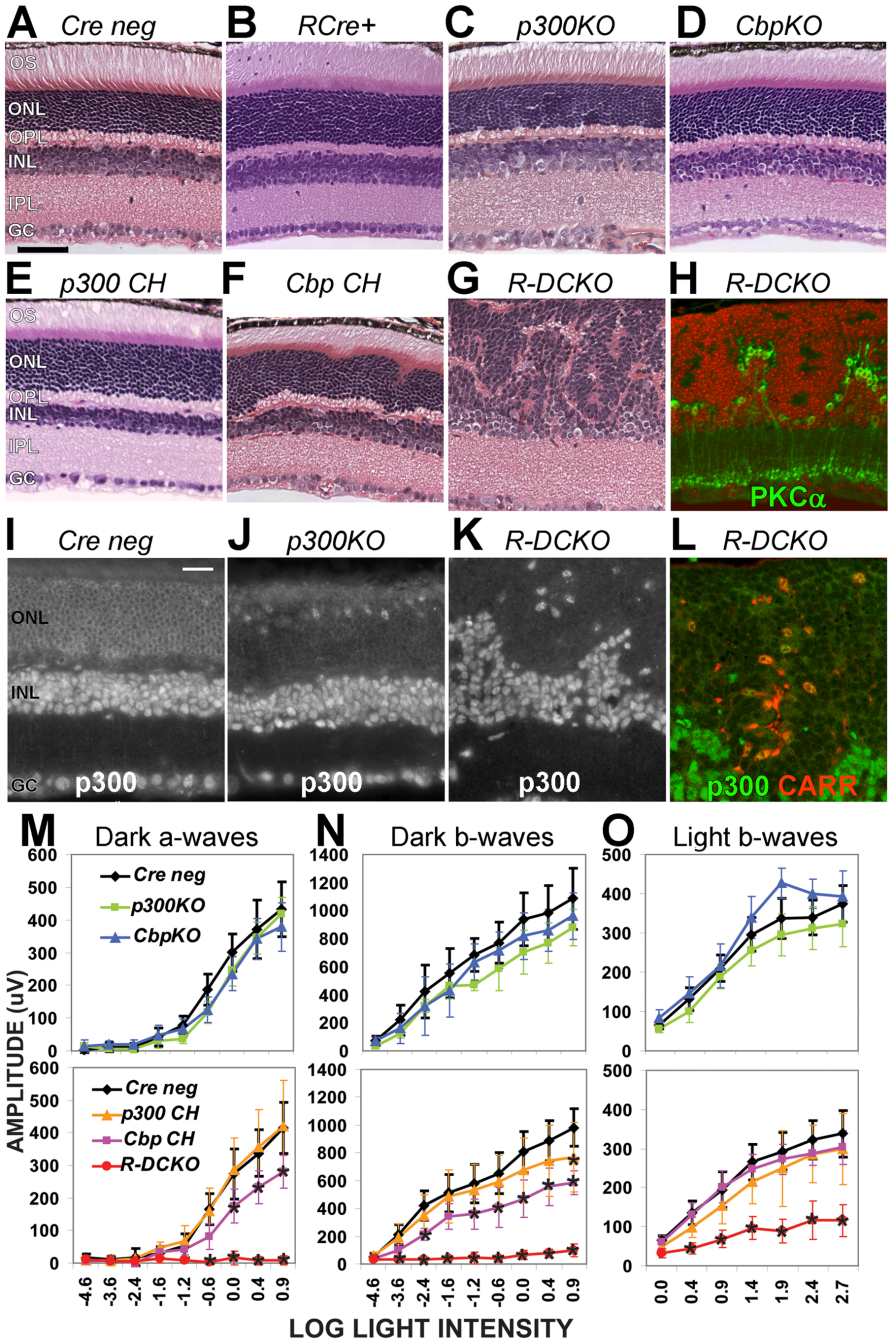
Knockout of both *Ep300* and *Cbp* in rods disrupts photoreceptor architecture and function. **A**–**G.** Cross-sections of 4-week-old retinas of the indicated genotypes (see Table 1), stained with hematoxylin and eosin (H&E). **H.** Section from the same *R-DCKO* eye as in panel G, fluorescently labeled with anti-PKCα (green, for bipolar cells) and DAPI (red), to show the boundary between the outer and inner nuclear layers. Scale bar  = 50 μm for all 8 panels. **I**–**K.** Immunofluorescent staining for p300 protein verified expression in all nuclei in *Cre neg* controls (**I**). *Ep300* expression is lost in the outer nuclear layer (**ONL**) of *p300KO* (**J**) and *R-DCKO* (**K**) retinas. **L.** *R-DCKO* section stained for p300 (green) and cone arrestin (CARR, red), showing that the few remaining p300-positive cells in the outer retina are cones. Scale bar  = 20 μm for all 4 panels. **OS,** outer segments; **ONL,** outer nuclear layer; **OPL,** outer plexiform layer; **INL,** inner nuclear layer; **IPL,** inner plexiform layer; **GC,** ganglion cell layer. **M**–**O.** Amplitudes of dark-adapted (“Dark”) and light-adapted (“Light”) flash electroretinograms (ERG) at 4 weeks of age. Flash intensities (log [CdSec/M^2^]) are indicated on the X-axis. Error bars indicate +/− 1SD of the mean amplitude for 6 animals of each genotype tested. Two-way repeated measures ANOVA showed significant interactions between genotype and log light level at p<0.0001 for dark-adapted a-waves (Panel M), b-waves (Panel N), and light-adapted b-waves (Panel O). Asterisks (*) indicate values significantly different (p<0.001) from *Cre negative* controls in post-hoc tests.

The desired conditional knockout mice listed in Table 1 were generated by crossing mice carrying *floxed* alleles of either *Ep300* [19] or *Cbp* [39]. Each *floxed* allele contains two *LoxP* sites flanking a critical exon, and has been shown to result in depletion of the gene product in cells expressing *Cre recombinase*. To express *Cre* in either rods or cones, we obtained two *Cre* transgenic mouse lines, *rhodopsin* promoter driven-*Cre* (*Rho-iCre or “RCre”*, [45]) and human *red-green cone opsin* promoter driven-*Cre* (*HRGP-Cre or “CCre”*, [46]). *Cre* expression was confirmed by crossing these lines to *ROSA-mTmG Cre* reporter mice, which express membrane-bound green fluorescent protein (GFP) in the presence of CRE activity [47]. As expected, the “*RCre*; *mTmG”* mice express *Cre* in differentiated rods beginning at postnatal day 5 (P5), peaking at P12 and continuing through adulthood (Supplemental Fig. S1A) without affecting retinal morphology or function up to 30 weeks of age (data not shown). In contrast, the “*CCre*; *mTmG”* mice show typical cone patterns of *Cre* expression, starting in a few cells in the ONL at P5, peaking around P12, and continuing through adult ages, with CRE activity restricted to cells in the outer margin of the outer nuclear layer (ONL), where cone cell bodies reside (Supplemental Fig. S1B). The similar expression pattern in dorsal vs. ventral regions suggests that *CCre*-driven *Cre* is expressed in both M-cones (enriched in the dorsal retina) and S-cones (enriched in the ventral retina) (reviewed in [3]). *CCre* mice also have normal retinal morphology and function. *RCre* and *CCre* lines were then mated with mice carrying *Ep300 flox*, *Cbp flox*, or both, to generate mice with the various conditional knockout (CKO) genotypes listed in Table 1. All *CKO* mice are viable and healthy without apparent abnormalities. CRE-mediated loss of p300 or CBP in photoreceptors was confirmed by immunostaining using anti-p300 (Fig. 1I-L) or anti-CBP (Supplemental Fig. S1C) antibodies. Thus, we have successfully created conditional knockouts of *Ep300, Cbp* or both in either rods or cones.

**Table 1:** Genotypes of mice used in this study.

Cre	Flox Genotype	Designated
*Rho-iCre+* [45]	*Ep300^wt^; Cbp^wt^*	*RCre+*
*Rho-iCre+*	*Ep300^wt^; Cbp^f/f^* [39]	*CbpKO*
*Rho-iCre+*	*Ep300^f/f^* [19]; *Cbp^wt^*	*p300KO*
-	*Ep300^f/f^; Cbp^f/f^*	*Cre neg*
*Rho-iCre+*	*Ep300^f/f^; Cbp^+/f^*	*Cbp CH*
*Rho-iCre+*	*Ep300^+/f^; Cbp^f/f^*	*P300 CH*
*Rho-iCre+*	*Ep300^f/f^; Cbp^f/f^*	*R-DCKO*
*HRGP-Cre+* [46]	*Ep300^f/f^; Cbp^+/f^*	*C-Cbp CH*
*HRGP-Cre+*	*Ep300^+/f^; Cbp^f/f^*	*C-p300 CH*
*HRGP-Cre+*	*Ep300^f/f^; Cbp^f/f^*	*C-DCKO*
*Rho-iCre+*	*tm4(ACTB-tdTomato,-EGFP)* [47]	*R-mT/mG*
*HRGP-Cre+*	*tm4(ACTB-tdTomato,-EGFP)*	*C-mT/mG*

### Loss of p300 and CBP in rods disrupts retinal structure and function

Hematoxylin-and-eosin (H&E) stained retina sections from four-week-old mice with rod-specific knockout of *Ep300* and/or *Cbp* (see Table 1) were examined for morphological defects. Compared to the *Cre-negative* (“*Cre-neg*”, Fig. 1A) and *Rho-iCre* (“*RCre+”,* Fig. 1B) controls, single conditional knockout of either *Ep300* (“*p300KO”,* Fig. 1C) or *Cbp* (“*CbpKO”,* Fig. 1D) in rods has little effect on retinal morphology. No apparent morphological changes were detected in these retinas up to 30 weeks of age (data not shown). In contrast, knocking out both *Ep300* and *Cbp* together (“*R-DCKO”*, Fig. 1G&H) severely disrupts lamination of the outer (ONL) and inner (INL) nuclear layers. The ONL forms whorls and rosettes, the cells fail to develop outer segments (OS), and the outer plexiform layer (OPL), where ONL and INL neurons form synaptic connections, is irregular and thin. INL cells positive for the rod on-bipolar cell marker Protein Kinase Cα (PKCα Fig. 1H) that also express P300 (Fig. 1K&L) extend into the ONL in the spaces between the rosettes. The total width of this disorganized retina is about 30–50% greater than control retina sections (data not shown). Cones in *R-DCKO* retinas are scattered throughout the ONL, often in the middle of rosettes of p300/CBP-negative cells (Fig. 1L, red cells), instead of evenly distributed in the outer part of the ONL as in normal retinas (Supplemental Fig. S2A&B). Interestingly, the defects seen in *R-DCKO* retinas are mostly prevented in compound heterozygous mice carrying one wild-type (WT) allele of either *Ep300* (“*p300 CH”*, Fig. 1E) or *Cbp* (“*Cbp CH”*, Fig. 1F), suggesting that p300 and CBP play redundant but critical roles in developing and maintaining appropriate retina architecture. However, in contrast to the essentially normal appearance of the *p300 CH* retina, *Cbp CH* retinas show slight irregularities in the ONL and OPL within 1000 µm of the optic nerve head (Fig. 1F).

Electroretinogram (ERG) testing at 4 weeks of age revealed functional deficits consistent with the morphological changes. Single conditional knockout mice had essentially normal ERGs (Fig. 1M, N&O, top graphs, green & blue vs. black lines). *R-DCKO* mice had very little rod-driven response (Fig. 1M&N, bottom graphs, red lines), and cone ERG responses were significantly smaller than those of *Cre-negative* littermates (Fig. 1O, bottom graph, red vs. black line). Compound heterozygote mice with one WT *Ep300* allele (*p300 CH*) showed essentially normal rod and cone function (Fig. 1M, N&O, bottom panels, orange line). In contrast, mice with a single WT copy of *Cbp* (*Cbp CH*) showed slight but significant decreases in rod-driven a-wave and b-wave amplitudes (Fig. 1M&N, bottom panels, pink line). Together, the ERG results are consistent with the morphological findings, and suggest that p300 and CBP play mostly overlapping roles in retinal structure and function.

**Figure 2:**
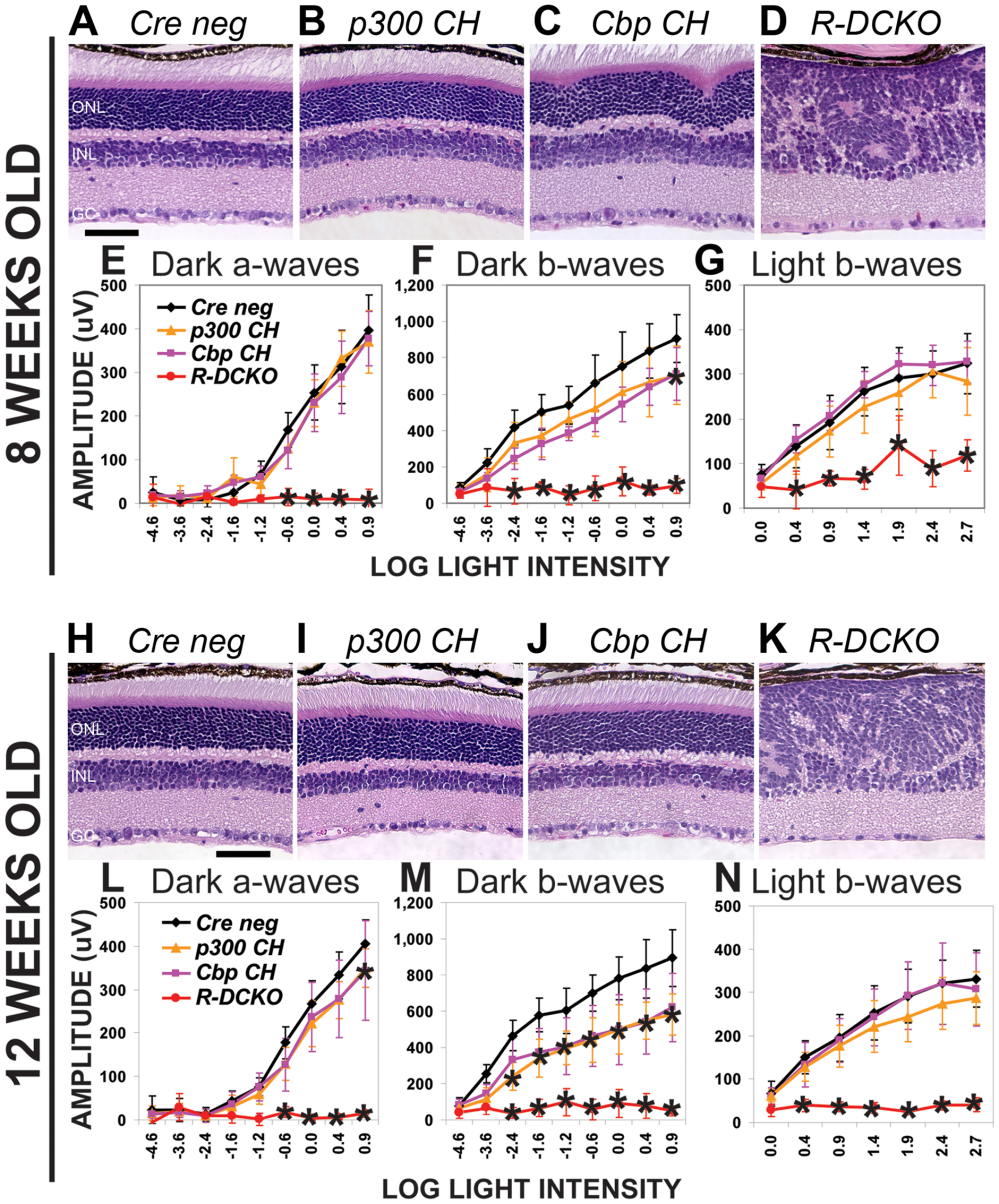
Compound heterozygotes show age-dependent phenotypes. **A**–**D.** Cross-sections of 8-week-old retinas stained with H&E show disrupted morphology similar to that seen at 4 weeks in *Cbp CH* and *R-DCKO* mice. Scale bar  = 50 μm. **E**–**G.** ERG testing shows persistence of the functional impairment in *R-DCKO* retinas (panels F and G). Dark-adapted b-wave deficits in some *p300 CH* mice tested at this time are reflected in the slightly decreased average and broad error bars for this genotype (Panel F orange line). Two-way repeated measures ANOVA indicated significance at p<0.0001 for dark-adapted a-waves (Panel E), b-waves (Panel F), and light-adapted b-waves (Panel G). **H**–**K.** Cross-sections of 12-week-old retinas stained with H&E. Morphologic abnormalities in *Cbp CH* retinas (panel J) have resolved, although whorls and rosettes are still seen in *R-DCKO* retinas (panel K). Scale bar  = 50 μm. **L**–**N.** ERG testing at 12 weeks revealed decreases in function in both *Cbp CH* and *P300 CH*, and *R-DCKO* retinas have lost cone responses in addition to rod function. Two-way repeated measures ANOVA indicated significance at p<0.0001 for dark-adapted a-waves (Panel L), b-waves (Panel M), and light-adapted b-waves (Panel N). Asterisks (*) indicate p<0.001 vs. *Cre negative* controls in post-hoc tests.

To determine if the structural and functional defects in *Ep300/Cbp* conditional knockout mice progress over time, retinal morphology and function were reassessed at 8 and 12 weeks of age. Fig. 2 shows that, while *R-DCKO* mice show no additional changes in retina morphology (Fig. 2D&K vs. Fig. 1G) or rod-driven ERG function (Fig. 2E, F, L&M, red lines, vs Fig. 1M&N, red lines) at either age, cone ERG responses worsened (Fig. 2G&N vs Fig. 1O, red lines), consistent with progressive loss of cones with age (data not shown). The structural and functional defects in *R-DCKO* retinas persist throughout the life of the animals (Fig. 2 and additional data not shown). Interestingly, ONL morphology of the compound heterozygous *Cbp CH* mice improved with age: the ONL irregularities near the optic nerve head (Fig. 1F & Fig. 2C) completely resolved by 12 weeks of age (Fig. 2J). However, the rod-driven ERG remained defective in *Cbp CH* mice (Fig. 2F&M, pink lines). The *p300 CH* mice also developed decreased rod ERG responses (Fig. 2F&M, orange lines) relative to *Cre-negative* littermates (black lines) as they aged, despite the normal appearance of their retinas at all ages, and normal rod ERGs at 1 month of age (Fig. 1N). These results suggest that a single copy of either *Ep300* or *Cbp* is insufficient to maintain normal retinal function throughout life.

**Figure 3:**
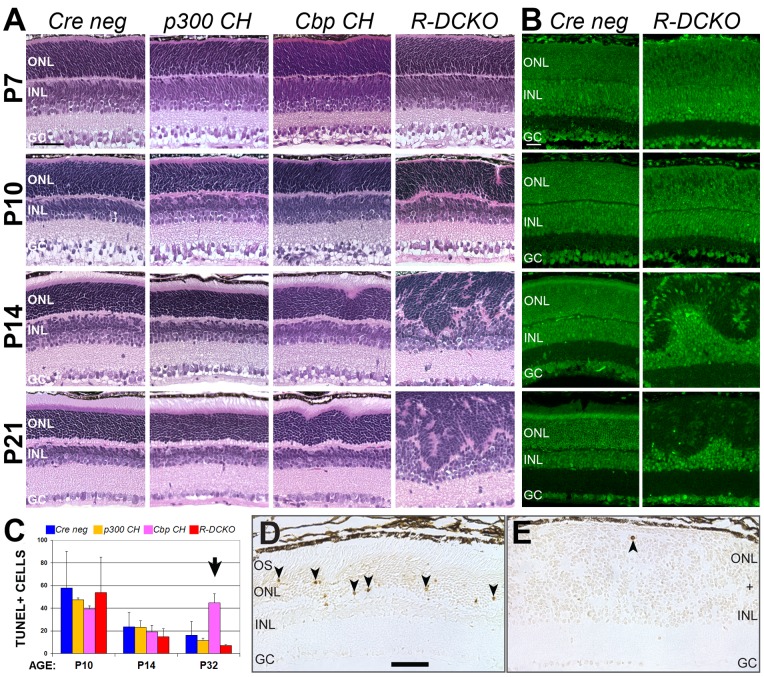
Development of conditional knockout phenotypes. **A.** H&E-stained sagittal sections of retinas from mice representing each indicated genotype (see Table 1), at the indicated ages. Irregularities appear in the ONL of *R-DCKO* mice at P10, and in *Cbp CH* retinas at P14. Scale bar  = 50 μm. **B.** Immunofluorescence staining for p300 (green) shows progressive loss of p300 from ONL cells of *R-DCKO* retinas between P7 and P14. Scale bar  = 25 μm. **C.** TUNEL staining for cell death was performed on three retinas of each genotype at P10, P14, and P32. TUNEL positive cells were only increased relative to age-matched *Cre negative* controls in *Cbp CH* retinas at P32 (arrow). **D.** P32 *Cbp CH* retina showing TUNEL positive cells (black arrowheads). These are frequently seen near ONL irregularities. **OS**, outer segments. Scale bar  = 50 μm. **E.** P32 *R-DCKO* retina containing one TUNEL+ cell (black arrowhead). *R-DCKO* retinas do not show increased cell death relative to *Cre-negative* littermates at any age examined.

To determine if the onset of the morphological disruption in *R-DCKO* and *Cbp CH* mice correlated with the loss of *p300/CBP* expression, retinas were examined histologically at P7, P10, P14 and P21 (Fig. 3A). Control and *R-DCKO* retinas were also examined for p300 immunoreactivity (Fig. 3B). At P7, before morphologic abnormalities appear in *R-DCKO* retinas, a few ONL cells show decreased p300 staining. At P10, as the number of p300-negative cells increases, irregularities in ONL structure begin to appear in *R-DCKO* but not compound heterozygote retinas. By P14, whorls and rosettes are seen throughout the *R-DCKO* ONL, and most cells in this layer have lost p300 (and CBP, data not shown) immunoreactivity. By P21, the only remaining p300-positive cells in the outer retina of *R-DCKO* also react with antibodies to cone proteins (see Fig. 1L and Supplemental Fig. S2A&B), indicating that they are displaced cone photoreceptors. Thus, the severity and timing of phenotype development directly correlate with the loss of expression of *Ep300/Cbp* in the conditional knockout retinas. Similar temporal development of the *Cbp CH* phenotype was also detected: Irregularities are first apparent at P14 (Fig. 3A) and persist through 8 weeks of age (Fig. 2C), but resolve by 12 weeks (Fig. 2J).

To determine whether cell death contributes to the structural and functional defects, we stained for cells containing fragmented DNA using the “Terminal Deoxynucleotidyl Transferase dUTP Nick End Labeling” (TUNEL) assay [48]. When TUNEL-positive cells in retina sections from P10, P14, and P32 animals were counted (Fig. 3C), the only significant difference from age-matched *Cre-negative* control eyes was seen in *Cbp CH* eyes at P32 (pink bar indicated by arrow in Fig. 3C). As shown in Fig. 3D, TUNEL-positive cells in these eyes were frequently found near the abnormal ONL folds, suggesting that these morphologic abnormalities may be resolved through cell death, as has been reported in the *Nr2e3*-null mutant mouse *rd7* [49]. *R-DCKO* retinas showed very few TUNEL-positive cells at this age (Fig. 3E). Immunostaining for histone H2A.X (Supplemental Fig. S3A), which recognizes double-stranded DNA breaks, supported this finding. Cell proliferation in the retina is mostly complete by the time *Rho-iCre* expression begins at P5, but re-entry of CKO cells into the cell cycle could contribute to the abnormal morphology of *R-DCKO* retinas. To investigate whether this occurs, P7 – P14 retinal sections were stained for proliferation markers Ki67 and phosphorylated histone H3 (Supplemental Fig. S3B shows P14, Supplemental Fig. S3C shows P10). Very few cells were positive for either marker in sections from any genotype at any of these ages, and no significant differences were found between *Cre-negative* and *R-DCKO* sections. *R-DCKO* outer retina cells also did not show increases in markers associated with other mature retina cell types (Supplemental Fig. S2) or retinal progenitors (Supplemental Fig. S4). These results suggest that conditional knockout of p300/CBP in differentiated rods does not increase either cell death or proliferation, or lead to a change in cell fate.

### Loss of p300 and CBP in rods alters the characteristic nuclear chromatin organization

**Figure 4:**
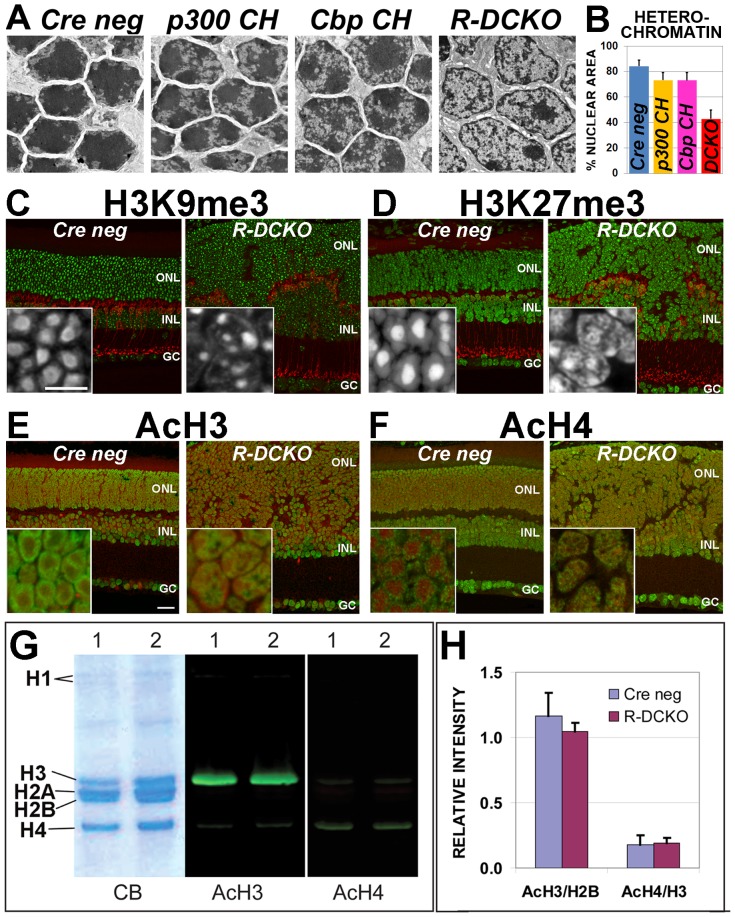
Loss of p300/CBP leads to chromatin decondensation and changes in distribution of histone marks in *R-DCKO* nuclei. **A.** Electron micrographs of nuclei in the ONL of P22 retinas. Compared to *Cre neg* control littermates, compound heterozygotes (*p300 CH* and *Cbp CH*) show slight increases in euchromatin (light areas within nuclei). In *R-DCKO* nuclei areas of euchromatin are greatly increased, and electron-dense heterochromatin appears reduced. **B.** Heterochromatin was quantified as a percentage of the total nuclear area in 50 nuclei from 10 micrographs for each genotype. Error bars  = 1 SD. Differences from *Cre neg* values were significant at p<0.0001. **C & D.** Comparison of immunoreactivity patterns for repressive histone marks H3K9me3 (green in panel C, white in insets) and H3K27me3 (green in panel D, white in insets) in control (left image) and *R-DCKO* (right image) retinas confirm loss of the characteristic rod chromatin condensation pattern in *R-DCKO* outer retina cells. Anti-PKC-alpha (red) marks bipolar cells. **E & F.** Comparison of immunoreactivity patterns for acetylated histone H3 (AcH3, green in panel D) and H4 (AcH4, green in panel E) reveals the redistribution of these activation marks in *R-DCKO* cells, corresponding to loss of the characteristic peripheral rod euchromatin distribution pattern. DNA is counterstained with Draq-5 (red). Scale bars: cross-sections  = 20 µm, insets  = 10 µm. **G.** Western blots of acid-extracted retinal histones from 15-week-old *Cre-negative* (1) or *R-DCKO* (2) retinas. CB, Coomassie blue stained gel. AcH3, blot stained for acetylated histone H3; AcH4, blot stained for acetylated histone H4. **H.** Quantification of band fluorescence intensities for AcH3 levels relative to total H2B levels, and AcH4 levels relative to total H3 levels at P20 did not show significant differences between *Cre neg* and *R-DCKO* samples.

Rod photoreceptors in the mouse retina have a unique chromatin organization: After birth but before the eyes open, dense, gene-poor heterochromatin usually found at the nuclear periphery in other cell types is positioned at the center of rod cell nuclei, surrounded by a peripheral ring of euchromatin (Fig. 4A, “*Cre neg*”) enriched for transcriptionally active DNA [44] [50]. In *R-DCKO* mice, the rods begin to develop this unique chromatin organization around P10, but the process reverses after P12 (data not shown). Most cells in the adult *R-DCKO* ONL show dense heterochromatin in a thin ring at the nuclear periphery and one or two small condensations in the inner nucleus (Fig. 4A, *“R-DCKO”*), a chromatin distribution pattern more similar to inner nuclear layer cells than to wild-type rods. Retinas from both compound heterozygote genotypes also show increases in electron-lucent euchromatin, but retain large dense heterochromatin domains in the center of their nuclei (Fig. 4A, *“p300 CH”* and *“Cbp CH”*). Quantification of the percentage of total nuclear area taken up by heterochromatin (Fig. 4B) shows that heterochromatin is decreased relative to euchromatin in these CKO retinas. These results suggest that *Ep300/Cbp* are involved in maintaining the characteristic nuclear chromatin organization in mouse rods.

Acetylated and methylated histone marks associated with gene activation and repression define concentric chromatin domains in wild-type mouse rod photoreceptor nuclei [49]. “Repressive” marks (trimethylation of H3K27, H3K9 and H4K20) are typically localized to heterochromatin in the dense nuclear core. “Activation” marks associated with euchromatin (H3 and H4 acetylation and H3K4 tri-methylation) are localized to the nuclear periphery, where transcription takes place. We therefore compared the distribution of two repressive histone marks: H3K9me3 (Fig. 4C) and H3K27me3 (Fig. 4D), and two activation marks: AcH3 (Fig. 4E) and AcH4 (Fig. 4F), in *Cre-negative* and *R-DCKO* retinas. The two repressive marks are redistributed in *R-DCKO* rod nuclei: unlike the large area of intense staining in the center of control rod nuclei, *R-DCKO* retinas show smaller, fragmented areas of intense reactivity with a weak ring of stain at the nuclear periphery (Fig. 4C&D). The two activation marks, which are found in a ring near the nuclear periphery in control rods, form scattered speckles in the middle of *R-DCKO* ONL cell nuclei (Fig. 4E&F). Thus, the nuclear organization of histone-marked territories is altered in *R-DCKO* rods, consistent with the heterochromatin and euchromatin redistribution seen by ultrastructural analysis.

To determine whether this altered nuclear organization reflects changes in global levels of acetylated histone H3 and H4, the products of p300/CBP and other acetyltransferases, quantitative Western blots were performed using the Li-Cor Odyssey Infrared Imager to compare AcH3 and AcH4 levels of *R-DCKO* vs. control retinas (Fig. 4G&H). No significant changes in total retinal AcH3 and AcH4 levels were detected in 4-week old *R-DCKO* vs. control retinas, as measured by fluorescent band intensities normalized to total histone H2B or H3 bands (Fig. 4H). Similar results were obtained at P14, P20 and 6 weeks (data not shown). Since rods constitute 70% of cells in the mouse retina, these results, together with the similar overall intensity of AcH3/AcH4 immunostaining of *R-DCKO* vs control retinas, suggest that p300/CBP depletion does not alter total levels of acetylated histone H3 and H4, although their distribution within the nucleus is affected.

### Loss of p300 and CBP in rods alters gene transcription and promoter-bound acetylated H3/H4

**Figure 5:**
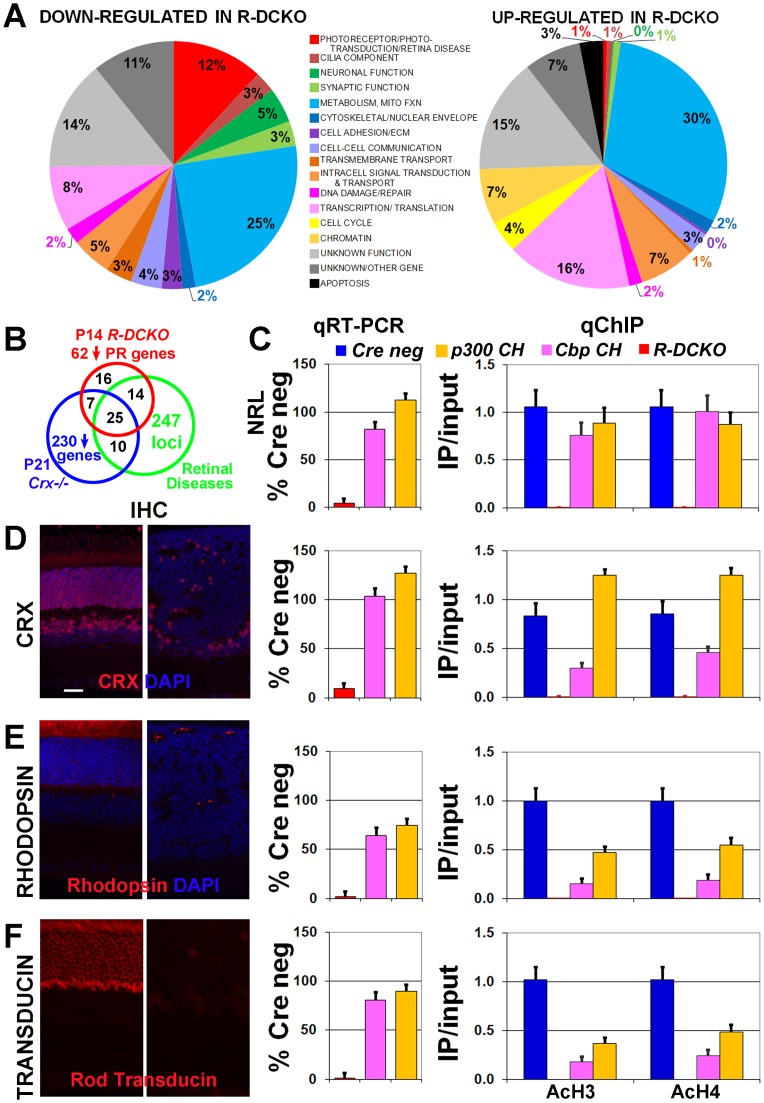
Expression of photoreceptor genes is decreased in *R-DCKO* retinas. **A.** Summarized microarray findings for *R-DCKO* vs. *Cre neg* retinas. Each gene was categorized by the cell process in which it functioned, and results for each category are represented as a percentage of all the down- or up-regulated genes (see Supplemental Tables 2 & 3 for details). **B.** Schematic distribution of the 62 down-regulated photoreceptor or phototransduction-related genes in *R-DCKO* microarrays (red), compared with the 247 retinal disease loci listed in RetNet (https://sph.uth.edu/RetNet/home.htm; green) and a list of 230 genes down-regulated in *Crx^−/−^* retinas compiled from published sources [51]–[53]. Numbers in overlapping areas indicate the numbers of genes affected in both/all three conditions. All overlapping genes are listed in Supplementary Tables S4 and S5. **C**–**F.** Expression of the indicated rod gene (**C.** ***Nrl*** **; D.** ***Crx;*** **E.** ***Rhodopsin (Rho);*** **F.** ***Rod Transducin (Gnat1)***) was assessed by quantitative RT-PCR (**qRT-PCR**) at P14, and is expressed as percent of the level of *Cre-negative* littermate controls (***% Cre neg***). Protein localization was verified by immunohistochemistry (**IHC**) at P30. Scale bar  = 20 µm for all images. Levels of acetylated histone H3 **(AcH3)** or H4 **(AcH4)** on the respective promoter was determined by quantitative chromatin immunoprecipitation (**qChIP**) at P14, and is expressed as the value from the immunoprecipitated sample divided by the value from the untreated “input” sample, multiplied by 100 (“IP/input”).

To investigate global gene expression changes underlying the morphological and functional defects in *R-DCKO* eyes, we performed microarray analysis on P14 whole retina samples. P14 was chosen because 1) CRE-mediated p300/CBP depletion is complete at this age (Fig. 3B); 2) previous findings showed that CRX-dependent p300/CBP recruitment to CRX target genes reaches a peak at this age [5]; and 3) secondary effects on bipolar, horizontal, and Mueller cell gene expression should be minimal this early. Three samples representing each genotype were tested in triplicate on Illumina BeadArray Mouse WG-6 V2 chips. Each microarray sample contained pooled RNA from a male and female littermate of the same genotype, to control for any sex-related differences. The raw datasets are available through the NCBI GEO website (http://www.ncbi.nlm.nih.gov/geo/, accession number GSE47699). Results were examined using Illumina GenomeStudio V1.6 software, and genes with differential scores greater than 13.0 (*P*<0.05) vs the *Cre-negative* control group were considered differentially expressed. Dramatic changes in expression were seen in the *R-DCKO* retinas: 520 genes were differentially down-regulated (Supplemental Table S2) and 579 were up-regulated (Supplemental Table S3). The wide variety of functions affected (Fig. 5A) indicates that p300 and CBP are involved in expression of many genes required for general cell maintenance as well as photoreceptor-specific structures and functions.

Many of the photoreceptor genes down-regulated in *R-DCKO* retinas appear in published lists of retina disease genes (https://sph.uth.edu/RetNet/home.htm), and genes affected in retinas of mice with knockout of the earliest expressed photoreceptor regulatory gene, *Crx* [51] [52] [53]. Figure 5B shows a Venn diagram summarizing the overlap between these gene sets and the 62 photoreceptor-related genes decreased in *R-DCKO* retinas. *Ep300/Cbp* conditional knockout decreased expression of 45 genes associated with retinal diseases (Supplemental Table S4). Interestingly, three additional retinal disease genes expressed in rods were up-regulated in *R-DCKO* retinas: *“Topoisomerase I Binding, Arginine/serine-rich”* (*Topors,* 147% of control levels), *“Transmembrane Protein 126A”* (*Tmem126a,* 160%), and “*Retinitis Pigmentosa 9*” (*Rp9,* 232%). Among the genes down-regulated in *R-DCKO* retinas, 101 were also reported decreased in *Crx^−/−^* retinas in other studies [51] [52] [53] (Supplemental Table S5). These genes are likely components of the CRX/NRL pathway in rods, consistent with p300/CBP acting as a coactivator. We were not surprised that cone-specific CRX downstream targets, such as cone *opsins*, *transducin* (*Gnat2*) and *phosphodiesterase* (*Pde6c*), were not affected in *Rho-iCre* conditional knockout mice, since *Crx* functions in both rods and cones but *Rho-iCre* only drives *Ep300/Cbp* conditional knockout in rods.

Quantitative RT-PCR (qRT-PCR) and immunohistochemistry (IHC) were used to confirm decreases in expression of a subset of rod photoreceptor genes (Fig. 5C–F and Supplemental Fig. S5A). AcH3/AcH4 levels on the promoter regions of these genes were also investigated by quantitative chromatin immunoprecipitation (qChIP) (Fig. 5C–F and Supplemental Fig. S5B). Importantly, in *R-DCKO* retinas, both microarray and qRT-PCR assays revealed drastically reduced expression of *Nrl*, the rod-specific transcription factor essential for rod identity (23% and 12% of control expression levels, respectively) and *Crx*, the pan rod/cone transcription factor (31% and 29% of controls) (Fig. 5C&D, red bars in qRT-PCR graphs, and Supplemental Tables S2, S4 and S5). IHC showed that the defect in *Crx* expression is localized to p300/CBP-negative cells (Fig. 5D far left panel and data not shown), indicating a cell-autonomous effect of *Ep300/Cbp* null mutations on *Crx* transcription in target cells. In *R-DCKO* retinas transcription of *Rhodopsin (Rho,* Fig. 5E, red bar in center graph) and *Rod transducin (Gnat1,* Fig. 5F) was abolished (6% and 4% of control levels), consistent with the microarray results (8% and 15% of control levels). Immunostaining also confirmed little expression of RHO or rod transducin in the *R-DCKO* outer retina (Fig. 5E&F, left panels). Marked decreases in transcription of other rod genes *Pde6b* (12% by qRT-PCR vs 14% by microarray), and *Rbp3* (31% by qRT-PCR vs 25% by microarray) were also confirmed (Supplemental Fig. S5A, red bars). *Cone opsin* (*Opn1MW* and *Opn1SW*) transcription was not affected (Supplemental Fig. S5A). In contrast to the rod-to-cone fate switch seen in *Nrl^−/−^* retinas [54] [55], p300/CBP*-*negative cells in *R-DCKO* retinas do not express cone markers, including cone arrestin (Fig. 1L) or cone opsins (Supplemental Fig. S2A&B).

Chromatin immunoprecipitation assays revealed that knockout of both *Ep300* and *Cbp* in rods completely removed AcH3 and AcH4 marks from the promoter of *Nrl*, *Crx*, *Rho, Gnat1* (Fig. 5C–F, right graphs, red bars), and *Pde6b* (Fig. S5C). These results are consistent with the lack of expression of these genes. AcH3 and AcH4 marks were moderately decreased on the cone *opsin* gene promoters in *R-DCKO* retina (Fig. S5B&C) despite the normal expression levels of these genes. The *Rbp3* promoter, which is expressed in both rods and cones, showed only slightly decreased levels of acetylated histones, comparable to levels on the promoter of the bipolar cell gene *“Metabotropic glutamate receptor type 6”* (*Grm6,* Fig. S5B), despite the decrease in transcription seen both by microarray (25% of controls, Supplemental Tables S2, S4, S5) and qRT-PCR (31% of controls, Supplemental Fig. S5A). These results suggest that AcH3/AcH4 level changes are gene- and cell-type specific.

In compound heterozygous mice (*Cbp CH or p300 CH*), one copy of either *Ep300* or *Cbp* was sufficient to preserve expression of both *Nrl* and *Crx* transcription factors (Fig. 5C&D, center graphs, pink and orange bars vs red), consistent with the higher AcH3 and AcH4 levels seen on the *Nrl* and *Crx* promoters in these retinas (Fig. 5C&D, right graphs, pink and orange bars vs. red). Expression of rod genes and promoter AcH3/AcH4 levels was partially restored (Fig. 5C–F and Supplemental Fig. S5), with *Ep300* (orange bars) more effective than *Cbp* (pink bars), consistent with the morphological and functional data. As expected, cone gene transcription was not affected in compound heterozygous mice (Supplemental Fig. S5A), although *Opn1MW* promoter AcH3/AcH4 levels were still moderately decreased (Supplemental Fig. S5B, orange and pink bars vs. blue). Taken together, the results of all expression assays suggest that p300/CBP keep chromatin containing rod transcription factors and structural genes in a transcriptionally active configuration (marked by AcH3/AcH4), to maintain rod cells in their terminally differentiated state.

### Loss of p300/CBP in cones causes defects in cone gene expression, structure and function

**Figure 6:**
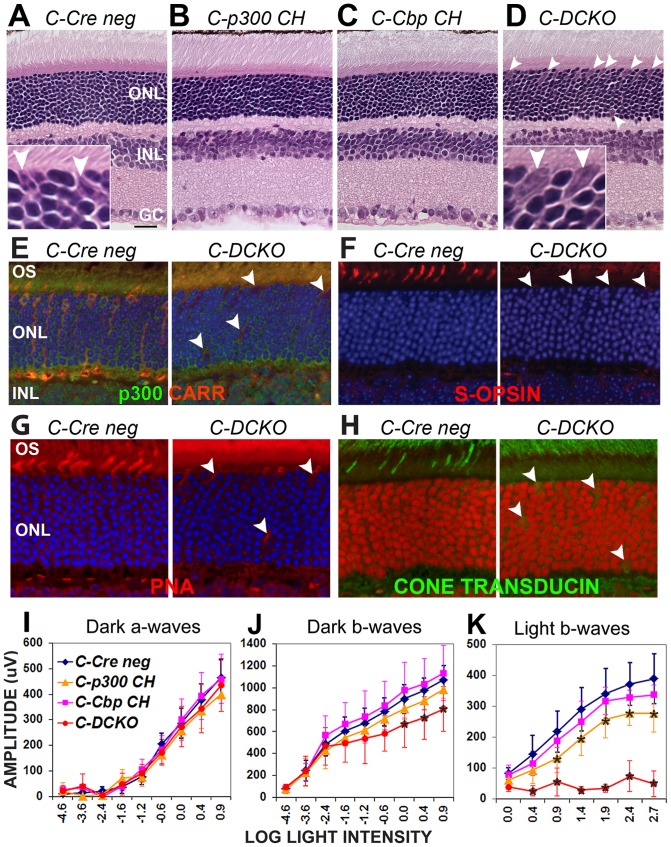
*Ep300/Cbp* conditional knockout in cones also disrupts cone structure, gene expression and function. *Cone opsin*-driven *Cre (CCre)* was used to knock out *Ep300/Cbp* in cone photoreceptors; morphology, cone gene expression/distribution, and ERG function were assessed at 6–7 weeks of age. **A**–**D.** Compared to *Cre negative* controls (Panel A; inset shows two presumptive cones), H&E staining of *CCre* conditional knockout retinas reveals no major abnormalities (panels B–D), but cells with large nuclei can be seen scattered throughout the outer retina in *C-DCKO* mice (Panel D arrowheads and high-magnification inset). **E.** Cone arrestin (red) and p300 (green) expression are decreased in these cells (arrowheads). **F.** S-opsin expression (red) is also decreased in these cells (arrowheads), which lack outer segments. **G.** Peanut agglutinin labelling (red) identifies the displaced, abnormal cells in the outer retina (arrowheads) as cones. Blue in Panels E–G is DAPI labelling of nuclei. **H.** Cone α-transducin (green) is decreased and mislocalized to the cell bodies. Draq5 (red) marks nuclei. **I.** ERGs performed on 6 week old *CCre* mice confirmed decreases in cone-driven responses in *C-DCKO* retinas (red lines). Two-way repeated measures ANOVA indicated significance at p<0.0001 for dark-adapted and light-adapted b-waves. Asterisks (*) indicate significance differences (p<0.001) from *Cre negative* controls in post-hoc tests.

The role of p300 and CBP in differentiated cones was examined using the cone-specific *Cre* line *HRGP-Cre* (*CCre*). Unlike knockout in rods, knockout of both *Ep300* and *Cbp* in cones (*C-DCKO*) did not lead to gross abnormalities in retinal morphology (Fig. 6D vs. 6A). However, the cone nuclei appeared enlarged and abnormally shaped (arrowheads and insets in Fig. 6D), and occasional similar cells are seen in the inner ONL or outer plexiform layer. These abnormal cones were not seen in the retinas of compound heterozygous mice carrying one normal allele of either *Ep300* (Fig. 6B “*C-p300 CH*”) or *Cbp* (Fig. 6C, *“C-Cbp CH*”). Furthermore, the cones in the double knockout retina showed defective outer segments marked by peanut agglutinin (Fig. 6G). The expression of the cone markers cone arrestin (CARR, Fig. 6E), S-opsin (Fig. 6F), and cone transducin-alpha (Fig. 6H) was markedly decreased. Consistent with these morphological and gene expression defects, *C-DCKO* mice completely lack cone ERG responses (Fig. 6K “light b-waves”, red vs. blue lines) without significantly affecting rod ERG responses (Fig. 6I “dark a-waves” graphs). The decrease in dark-adapted b-waves at high stimulus intensities (Fig. 6J) provides further evidence of a cone functional defect, since cones contribute to dark-adapted ERG responses to bright flashes. This cone ERG defect was less pronounced in mice with one normal copy of either *Ep300* (Fig. 6K, orange line) or *Cbp* (Fig. 6K, pink line). Thus, p300 and CBP also play a redundant role in maintaining cone gene expression, structure and function.

## Discussion

### p300 and CBP play overlapping and distinct roles in establishing photoreceptor structure and function and maintaining their cell identity

During postnatal mouse retinal development between P10 and P21, post-mitotic opsin-positive photoreceptors undergo terminal differentiation and maturation. At the cellular level, they elaborate outer segments containing the phototransduction machinery, and make synaptic connections to inner neurons. At the molecular level, expression of many photoreceptor genes increases to adult levels during this time [5]. All these terminal differentiation processes depend on continuing expression of *Ep300* and/or *Cbp*. Although conditional knockout of both genes causes severe defects, one wild-type allele of either *Ep300* or *Cbp* can mostly prevent these defects, indicating that p300 and CBP are redundant for this critical role. These results agree with findings from a study of postmitotic mouse brain neurons [56], that loss of either p300 or CBP alone does not affect cell viability or cause severe defects. However, these investigators found modest memory and transcriptional deficits after brain-specific knockout of either *Ep300* or *Cbp*, whereas we did not observe any functional, structural and molecular consequences of knocking out either gene alone in retinal photoreceptor neurons. This could be due to the late expression of *Opsin*-driven *Cre* in already differentiated photoreceptors, which limits our ability to investigate the role of *Ep300*/*Cbp* in early photoreceptor differentiation from postmitotic precursors. Future studies using an early photoreceptor gene promoter to drive Cre expression will address whether either p300 or CBP alone is required for early photoreceptor development and if they play distinct functions in this process. Such studies are important for understanding the rod/cone dystrophy phenotypes of Rubinstein-Taybi syndrome (RSTS) [40], a disease associated with heterozygous *CBP* [33] or *Ep300* mutations [34]. However, the present study has provided some hints of distinct roles for p300 and CBP in photoreceptor terminal differentiation: Although one copy of either *Ep300* or *Cbp* essentially prevents the *R-DCKO* phenotype, mice expressing a single WT copy of *Cbp* show slight defects in rod morphology, function and gene expression, suggesting that p300 may have functions in photoreceptor maturation and maintenance that CBP cannot fulfill. In this regard, a recent study using a glioma-derived cell line [57] showed that p300 and CBP each binds some unique target gene promoters in addition to the numerous targets they share. Even when both factors bind and regulate the same gene in a given cell type, such as *αA-crystallin* in newborn mouse lens fibrocytes, they have been found differentially distributed along the locus [58], suggesting distinct regulatory mechanisms. Our own studies in *Crx^−/−^* mouse retina show that *Opsin* promoter occupancy by CBP, but not p300, requires Crx [5]. Thus, p300 may have a wider range of CRX-independent photoreceptor target genes than CBP, supporting distinct roles for these two coactivators in photoreceptor gene activation.

### Cellular mechanisms underlying the phenotypes of *Ep300/Cbp* conditional knockouts

#### Cell autonomous and non-autonomous effects

The severely disrupted retinal morphology and photoreceptor function in rod-specific knockout of CBP/p300 (*R-DCKO*) suggest the involvement of both cell autonomous and non-autonomous mechanisms. The cone dysfunction and gene expression defects are likely secondary to ONL disorganization. Cone cell death often occurs in retinas with rod degeneration disorders. It is known that support provided by RPE and soluble growth factors secreted by rods play important roles for cone integrity and survival. In *R-DCKO* retinas, many cones are displaced in the center of whorls and rosettes where they are not in contact with the RPE, which prevents them from getting metabolic support from the RPE. The p300/CBP-negative “rods” likely fail to express protective growth factors/cytokines as well as other rod-specific genes. At least one such factor, rod-derived cone viability factor [59] encoded by the gene *nucleoredoxin-like 2 (Nxnl2)* [60], is down-regulated (11% of control levels) in *R-DCKO* retinas (Supplemental Tables S2 & S5). Secondary, cell non-autonomous defects may also account for *R-DCKO*'s invasive inner nuclear layer (INL) and thin/irregular outer plexiform layer (OPL), where synapses between photoreceptors and inner retina neurons are located. Inner retina abnormalities and remodeling are often seen in late stages of photoreceptor degeneration diseases [61], including the *Crx^−/−^* mouse model. In contrast, the cone-specific knockout *C-DCKO* essentially shows cell autonomous defects in cones without affecting rod structure and function. It remains to be determined whether any changes occur in inner neurons of the *C-DCKO* retina.

#### Cellular basis for whorls and rosettes in R-DCKO

Abnormal retinal folds are often seen in mouse models where the rods undergo a cell fate switch to cones (*Nrl^−/−^* or *Nr2e3^rd7/rd7^*) [49] [54] [62], or which compromise the retinal outer limiting membrane (OLM), composed of adherens junctions between photoreceptors and Müller glia (the naturally occurring *Crumbs 1* mutation *Crb1^rd8/rd8^*, or knockout of the *LIM homeobox protein Lhx2*) [63] [64]. The severe retinal “whorls and rosettes” phenotype seen in *R-DCKO* is likely contributed by both mechanisms: 1) *Nrl* expression is decreased in *R-DCKO* rods, leading to the loss of rod identity and increase in nuclear volume; 2) OLM abnormalities, which could be caused by defective adherens junctions or changes in cell polarity. Microarray showed that expression of both “*Crumbs 1” (Crb1)* and an associated protein *“membrane protein, palmitoylated 4”* (*Mpp4*) [65] are decreased (53% and 14% of controls, respectively). The above two mechanisms are not mutually exclusive, as a recent study has shown that OLM defects are associated with rosette development in the *Nrl^−/−^* mouse retina [66]. Finally, the retinas of *Sca7* mice also develop ONL whorls and rosettes with changes in the rod chromatin pattern [67], similar to those we see in our compound heterozygotes. *Sca7* mice have defects in the GCN5-containing STAGA transcriptional activation promoting complex. This implicates decreased lysine acetyltransferase and/or chromatin remodeling activity as an underlying cause of whorls and rosettes.

#### Cell survival vs. death

To our surprise, *R-DCKO* photoreceptors and inner retina neurons do not die but survive for a long period (at least through 30 weeks of age) despite down-regulation of many photoreceptor genes, normally a trigger for photoreceptor degeneration. *R-DCKO* cells do not re-enter the cell cycle, but remain in a quiescent state. The mechanism for this phenotype is unclear. This does not appear to be a form of senescence, since the microarray analysis showed no changes in expression of the senescence-associated “*Cyclin-dependent kinase inhibitor”* genes *CDKN1a/p21*, *CDKN2a/p16/INK4A*, or their downstream targets [68] in the *R-DCKO* retinas. Since p300 and CBP are required to activate transcription of lineage-specification-associated genes for cell fate switching [5] [69] as well as genes associated with cell proliferation and survival [70], one possible explanation is that the global and severe changes in gene expression in *R-DCKO* strand the cells in a transitional state, unable to either progress along an alternative lineage, regress, or die. Supporting this hypothesis, 4-week-old *Cbp CH* compound heterozygous mice did show increased cell death associated with minor ONL folds (Fig. 3C&D), suggesting that programmed cell death is induced in p300/CBP-deficient photoreceptors if minimal CBP protein is available. A search of the *R-DCKO* microarray data revealed up-regulation of 18 genes involved in cell death pathways, including: *“BCL2-interacting killer”* (*Bik*, 1873% of controls), *Caspase 6* (246%), *“Bcl2-associated X protein”* (*Bax,* 231%), *“apoptotic peptidase activating factor 1”* (*Apaf1,* 189%), *“CASP8 and FADD-like apoptosis regulator”* (*Cflar*, 163%), and *“BCL2-associated agonist of cell death”* (*Bad,* 150%) (Supplemental Table S3). BIK initiates apoptotic responses to genotoxic stress or disruption of host cell protein synthesis in processes like viral infections or treatment with proteasomal or protein synthesis inhibitors. It activates BAX via Ca^2+^ release from intracellular stores, leading to activation of the mitochondrial apoptotic pathway, and has also been implicated in an autophagy-mediated cell death pathway [71]. Thus, the high level of *Bik* activation likely represents an attempted response to the metabolic disruption caused by loss of the two general transcription coactivators. However, at this point we can only speculate that the failure of *R-DCKO* cells to die results from their failure to synthesize or activate late-stage effectors such as Caspase 9/3 or autophagic mediators, since these were not present among the up-regulated genes detected by microarray.

#### The identity of p300/CBP-negative cells

Gene expression and immunohistochemistry studies have shown that p300/CBP-negative ONL cells in *R-DCKO* have completely lost their photoreceptor identity. The true identity of these cells is unknown, but they appear to be undifferentiated cells that do not express any neuronal or glial progenitor markers. At the ultra-structural level, these cells still have neurites extending from their cell bodies (data not shown), suggesting their neuronal origin. This phenotype clearly demonstrates the requirement of p300/CBP in maintaining differentiated photoreceptors.

### Molecular mechanisms for transcriptional dysregulation in *Ep300/Cbp* conditional knockouts

Our results showed that knockout of *Ep300/Cbp* in rods or cones causes severe transcription defects in many genes expressed in photoreceptors, leading to a much more severe phenotype than is seen after knockout of any single photoreceptor transcription factor. This is understandable, since p300/CBP are cofactors for a wide variety of general as well as cell-type specific transcription factors. However, a substantial subset of the genes down-regulated in the microarray assay are involved in photoreceptor structure and function. Furthermore, conditional knockout of *Ep300/Cbp* leads to loss of photoreceptor identity. Our findings emphasize the need for continuing expression of these photoreceptor genes to maintain functional photoreceptors, and the role played by *Ep300/Cbp* in this expression.

This study has provided evidence of at least three mechanisms that directly underlie the transcriptional dysregulation in *DCKO* photoreceptors.

#### Decreased expression of key photoreceptor transcription factors

Transcription factors CRX, NRL and NR2E3 specify rod photoreceptor cell fate by regulating many photoreceptor genes. The expression of all three factors is lost from *R-DCKO* retinas, but preserved in retinas with a single normal copy of either *Ep300* or *Cbp*, suggesting that p300 and CBP are redundant in promoting the expression of these key regulator genes. Although severe transcription defects are expected in cells directed to the photoreceptor lineage that lack these transcription factors, the loss of coactivator/HAT function likely also contributes to the wide-spread transcription dysregulation seen by microarray analysis. Furthermore, transcription of photoreceptor genes remains defective to various degrees even when photoreceptor transcription factor expression is restored in the presence of one copy of *Ep300* or *Cbp*. Future experiments to restore the expression of CRX and NRL in *R-DCKO* retinas will separate the contribution made by the lack of transcription factors from the direct results of coactivator deficiency. Furthermore, genetic interaction studies in mice lacking transcription factor and coactivator pairs might help to determine the importance of functional interactions between a specific transcription factor and either coactivator.

#### Defects in histone acetylation

Quantitative Western blot assays showed that loss of p300 and CBP in rods did not alter the overall levels of acetylated histone H3/H4, suggesting that other factors (HATs or HDACs) are also involved in maintaining acetylated histone levels. However, an important finding is that knockout of p300/CBP selectively affected acetylated histone H3/H4 levels and transcription of all the CRX target genes tested. This raises the question whether loss of p300/CBP causes redistribution of acetylated histone H3/H4 among chromatin regions and gene sets being transcribed. Interestingly, microarray assays detected a similar number of genes that are up-regulated (n = 579) vs. down-regulated (n = 520). The relationship between p300/CBP-dependent histone acetylation and transcription remains to be investigated. Genome-wide ChIP assays on gene association of acetylated histone H3/H4 in the *Ep300/Cbp* mutant vs control retinas might provide additional information. On the other hand, this relationship is supported by the results from the *p300 CH* and *Cbp CH* compound heterozygous mice, where the levels of histone acetylation and target gene transcription are generally correlated. These results are consistent with our previous observations that histone acetylation precedes transcriptional activation of photoreceptor genes during development [5], and strongly support the importance of p300/CBP-mediated histone acetylation in initiating and maintaining photoreceptor gene transcription. It remains to be determined if the HAT catalytic activity of p300/CBP directly accounts for the observed histone acetylation defects, because both factors have additional coactivator functions [14] [35]. They interact with numerous non-HAT and HAT-harboring transcription regulators, including members of the KAT2 family, GCN5-KAT2A and PCAF/KAT2B [19]. GCN5 interacts with CRX indirectly via ataxin-7, a component of the STAGA coactivator complex involved in regulating histone acetylation and transcription of CRX target genes during retinal development [5] and in photoreceptor degeneration associated with Spinocerebellar Ataxia 7 polyglutamine expansion disease [67] [72] [73]. Thus, loss of the action of these HATs could potentially contribute to defective histone acetylation and transcription of photoreceptor genes in *Ep300/Cbp* knockout mice. In addition, levels of histone acetylation and transcription do not always show a linear relationship for each individual gene. For example, in *Cbp CH* mice the *Crx* promoter has less than 50% of normal AcH3/AcH4 levels, but *Crx* transcription appears normal (Fig. 5D, pink bars), suggesting that the effect of histone acetylation on transcription may depend on gene context. Finally, changes in histone acetylation might affect other histone modifications or higher order chromatin organization, which could also contribute to transcription dysregulation in the *Ep300/Cbp* knockout mice.

#### Disruption of normal nuclear chromatin organization

One important finding is that double conditional knockout retinas lose the characteristic densely packed chromatin in rod nuclei, and this phenotype is only partially prevented in *p300 CH* and *Cbp CH* compound heterozygous mice. During the late stages of photoreceptor development, mice and other nocturnal mammals reorganize their rod photoreceptor chromatin to minimize light scatter [44]. The condensed, transcription-poor heterochromatin is organized in the central area of the rod nucleus. The less condensed euchromatin containing transcription factors [67], activated RNA polymerase [50], splicing machinery and nascent RNA transcripts [44] are all localized to the nuclear periphery, indicating that this is the site of active gene transcription. The mechanisms responsible for establishing and maintaining this unique nuclear organization are being elucidated. In non-photoreceptor cells, two apparently parallel interactions involving nuclear Lamin A/C or the Lamin B receptor (LBR) anchor heterochromatin enriched in LINE elements to the nuclear periphery [74]. Mouse retinal cells initially use the Lamin B receptor interaction, then as they differentiate switch to the Lamin A/C mechanism, but rod photoreceptors never activate Lamin A/C expression. Loss of *Lbr* expression correlates with heterochromatin reorganization in normal mouse rods. Although expression of *Lbr* is not significantly increased in *R-DCKO* retinas, another receptor for Lamin B, *Lmnb2*, is upregulated (181% of Cre neg in microarray assays).

The rod transcription factor NRL appears to be involved in the reorganization of mouse rod nuclear chromatin. Mice in which *NRL* or its upstream activator *“Retinoid-related orphan nuclear receptor beta”* (*Rorb*) are knocked out fail to reorganize their heterochromatin, but in *Rorb^−/−^* mice expression of an *NRL* transgene driven by the *Crx* promoter restores rod nuclear morphology [75]. In addition, the process may involve the Retinoblastoma (RB) family of pocket proteins, which are active in developing mouse rod photoreceptors at the time when nuclear reorganization occurs. Heterochromatin condensation also fails to occur in the absence of RB [76]. The RB protein directs pericentric and telomeric heterochromatin formation [77] as well as recruiting histone deacetylases (HDAC) to active gene promoters to silence transcription (reviewed in [78]). Longworth and Dyson [79] suggest that RB functions as a “master regulator of chromatin structure”; expression of *Rb* and its mouse homologs *Rbl1*/*p107* and *Rbl2/p130* were unaffected in the microarray assays as expected if CBP/p300 are downstream effectors. Since rod nuclear chromatin also decondenses in mice with defects in STAGA complex (containing GCN5) [67], transcription coactivators with histone acetyltransferase (HAT) activity seem to be important for maintaining this chromatin architecture. It is unclear why the loss of coactivator HATs results in rod chromatin decondensation similar to that seen in the absence of RB, a repressor associated with HDACs, but these findings all point to the importance of homeostasis between histone acetylation and deacetylation in rod nuclear chromatin organization. Furthermore, our recent studies have demonstrated that the specific rod transcription factors CRX, NRL and NR2E3, play important roles in higher order chromatin organizations, such as chromosomal loops [80], which may contribute to the rod-specific nuclear architecture. A better understanding of how interactions between specific transcription factors and epigenetic modulators regulate rod nuclear organization will provide new insights into general chromatin regulatory mechanisms and how they relate to transcriptional regulation.

## Materials and Methods

### Ethics statement

This study was approved by the Animal Studies Committee of Washington University in St. Louis, and performed under Protocols # 20090359 and 20120246 (to SC). Mice were housed in a barrier facility operated and maintained by the Division of Comparative Medicine of Washington University School of Medicine. Experiments were carried out in strict accordance with recommendations in the Guide for the Care and Use of Laboratory Animals of the National Institutes of Health (NIH); the Washington University Policy on the Use of Animals in Research; and the Guidelines for the Use of Animals in Visual Research of the Association for Research in Ophthalmology and Visual Science (http://www.arvo.org/animals/). Every effort was made to minimize the animals' suffering, anxiety, and discomfort.

### Reagents

#### Mouse Lines

*Ep300 flox* mice [19] were obtained from Dr. Paul K. Brindle of St. Jude Children's Research Hospital, Memphis, TN. *Cbp Flox* mice [39] were obtained from Dr. Jan van Deursen, The Mayo Clinic, Rochester MN. *Rho-iCre* mice [45] were provided by Dr. Ching-Kang Jason Chen, Virginia Commonwealth University, Richmond, VA. *HGRP-Cre* mice [46] were provided by Dr. Yun Zheng Le, University of Oklahoma Health Sciences Center, Oklahoma City, OK. *ROSA-mTmG Cre* reporter mice (B6.129(Cg)-*Gt(ROSA)26Sor^tm4(ACTB-tdTomato,-EGFP)Luo^*) [47] were obtained commercially (Jax Mice Stock #007576, The Jackson Lab, Bar Harbor, ME). Mice used in this study were bred and housed in Washington University School of Medicine barrier facilities; the genotypes are listed in Table 1. Genotyping primers are listed in Supplemental Table 1. All mice used were negative for RD1 [81] and RD8 [82] mutations.

#### Antibodies

Used in these studies are listed in Supplemental Table S7. Rhodamine-conjugated peanut agglutinin (RL-1072) was obtained from Vector Laboratories, Burlingame, CA, and the TUNEL staining kit (S7100) from EMD Millipore, Billerica, MA.

### Methods

#### Electroretinography

At least 6 mice of each genotype were tested for ERG at 4, 6, 8 ,12, 20, 30, 40 and 52 weeks of age. Bilateral flash ERG measurements were performed using a UTAS-E3000 Visual Electrodiagnostic System running EM for Windows (LKC Technologies, Inc., Gaithersburg, MD). Mice were dark-adapted overnight, then anesthetized with 80 mg/kg ketamine and 15 mg/kg xylazine under dim red illumination for electrode placement and testing. Body temperature was maintained at 37±0.5°C with a heating pad controlled by a rectal temperature probe (FHC Inc., Bowdoin, ME). The mouse's head was positioned just inside the opening of the Ganzfeld dome and pupils were dilated with 1.0% atropine sulfate (Bausch & Lomb, Tampa, FL). The recording electrode was a platinum loop 2.0 mm in diameter, positioned in a drop of 1.25% hydroxypropyl methylcellulose (GONAK; Akorn Inc., Buffalo Grove, IL) on the corneal surface of each eye. The reference needle electrode was inserted under the skin at the vertex of the skull. The ground electrode was inserted under the skin of the mouse's back or tail. The stimulus (trial) consisted of a brief, full-field flash (10 µs) either in darkness, or in the presence of dim (30.0 cd/m^2^) background illumination after 10 minutes adaptation time to the background light. The initiation of the flash was taken as time zero. The response was recorded over 250 ms plus 25 ms of pre-trial baseline. Responses from several trials were averaged (see Supplemental Table S6). The amplitude of the a-wave was measured from the average pre-trial baseline to the most negative point of the average trace, and the b-wave amplitude from that point to the highest positive point, without subtracting oscillatory potentials. The log light intensity (log [^cd*s^/_m2_]) was calculated based on the manufacturer's calibrations. The amplitudes (in microvolts) of dark-adapted a- and b-waves and light-adapted b-waves were measured from the lowest point of the raw averaged response trace (occurring prior to 50 ms after the flash) to the subsequent highest point (oscillatory potentials were not subtracted). Responses from at least 6 mice of each genotype were compared for each time point. The distributions of ERG response across genotype groups at different light intensities were described by means and standard deviations. The between-group differences were compared using two-way ANOVA for repeated measurement data to account for potential correlations among readings from the same mice, followed by post-hoc multiple comparisons for differences between each genotype and the control group at each light intensity level. All the tests were two-sided and a p-value of 0.05 or less was taken to indicate statistical significance. The statistical analysis was performed using SAS 9.3 (SAS Institutes, Cary, NC). P-values were adjusted for multiple comparisons by a permutation test using the default parameters provided in the LSMestimate statement in Proc Mixed.

#### Histology and Immunohistochemistry

Eyes from at least three representative mice from each age and genotype group were examined histologically. Mice were sacrificed by pentobarbital overdose and eyes were dissected, immersion-fixed and corneas removed in 4% paraformaldehyde in phosphate buffered saline (PBS). After overnight incubation in fresh fixative, retinas were dehydrated and paraffin-embedded using a Tissue-Tek VIP tissue processor. Sagittal sections 4 µm thick were cut through the optic nerve head, mounted on poly-lysine-coated slides, air-dried, and either stained with hematoxylin and eosin (H&E) for morphologic assessment or used for immunohistochemistry. Antigen retrieval was performed by treating de-paraffinized sections in 0.1 M citrate buffer, pH 6.0 in a pressure cooker for 3 minutes. Cooled slides were blocked 30 minutes in 20% normal goat serum, incubated overnight at 4°C in primary antibody diluted in PBS; rinsed well, then incubated for one hour at room temperature in secondary antibodies conjugated to Alexa-fluor (Invitrogen) or Cy-dyes (Jackson Immunoresearch) diluted in PBS. For some samples, Draq5 (Cell Signaling Technology, Danvers, MA) for nuclear DNA was added to the secondary incubation mix at 1∶5000. After thorough rinsing, slides were coverslipped using Vectashield hard set mounting medium for fluorescence with (H-1500) or without (H-1000) DAPI (Vector Laboratories, Burlingame, CA). Sections were examined and photographed using an Olympus BX51 fluorescence microscope fitted with a Spot RT3 cooled CCD camera (Diagnostic Instruments, Inc). Confocal microscopy was performed on an Olympus Fluo-view FV1000 confocal microscope.

#### Electron microscopy

Eyecups were fixed by immersion for 12–24 hrs in 2% paraformaldehyde/3% glutaraldehyde in 0.1 M phosphate buffer (pH 7.35), post-fixed in 1% osmium tetroxide for 1 hour and stained *en bloc* with 1% uranyl acetate in 0.1 M acetate buffer for 1 hr. Blocks were then dehydrated in a graded series of acetones and embedded in Araldite 6005/EMbed 812 resin (Electron Microscopy Sciences). Semi-thin sections (0.5–1 µm) were cut through the entire retina at the level of the optic nerve and stained with toluidine blue. Ultra-thin sections were taken from a 600–800 µm length of retina adjacent to the optic nerve, post-stained with uranyl acetate and lead citrate, viewed on a Hitachi H7500 electron microscope and documented in digital images. For the 5 largest nuclear profiles in each of 10 micrographs of ONL cell bodies from each genotype, total nuclear area and the amount of dense heterochromatin as a percentage of total nuclear area was measured using NIH “Image J” software. For each genotype, the mean and standard deviation was calculated, and compared to values for *Cre negative* controls using paired Student's t-test.

#### Nuclear extraction and western blot analysis

The histone extraction protocol used is a modification of the protocol of Shechter et al. [83]. Briefly, retinas were dissected, washed in PBS with proteases (“cOmplete mini” tablets, Roche 11 836 153 001), and incubated 30 minutes at 4°C in hypotonic lysis buffer (10 mM Tris buffer, pH 8.0, with 1.0 mM KCl, 1.5 mM MgCl_2_, 1 mM DTT, 0.003 mM Trichostatin A and protease inhibitors including 2 mM PMSF. Nuclei were concentrated by centrifugation at 10,000xG for 10 minutes, then lysed by incubating overnight at 4°C in 0.4 N H_2_SO_4_. Histones were precipitated from the supernate with trifluoroacetic acid, washed twice with chilled acetone to remove the acid, air-dried, resuspended in deionized, distilled water and quantified on a NanoDrop ND-1000 spectrophotometer (NanoDrop Technologies, Wilmington, DE). Samples containing 1.0 µg protein were separated on a 17% SDS-PAGE gel (mini-protean TGX pre-cast gels, BioRad), blotted to nitrocellulose using a Transblot Turbo semi-dry transfer system (BioRad), and stained using Li-Cor Odyssey blocking reagent and secondary antibodies (Li-Cor, Lincoln, NE). Blots were imaged on a Li-Cor Odyssey Classic system and analyzed using Image Studio software.

#### Microarray

Retinas were removed from 3 male and 3 female P14 mice of each genotype, stored in RNAlater (Qiagen), and extracted using the RNeasy kit (Qiagen). Purified RNA was quantified on the NanoDrop and submitted to the Washington University Genome Technology Access Center for quality assessment with the Agilent Technologies 2100 Bioanalyzer “Lab-on-a-chip” system (Agilent Technologies, Santa Clara, CA). Equal amounts of high-quality (RIN score >8.4) RNA samples from one male and one female littermate were pooled for microarray assay on Illumina BeadArray Mouse WG-6 V2 chips (Illumina, San Diego, CA). The raw microarray data were submitted to NCBI GEO (accession number GSE47699). The results were examined using Illumina Genome Studio V1.6 software. Differential expression analysis (Direct hyb-differential expression) was performed using Average normalization and the Illumina custom error model with Benjamini and Hochberg False Discovery Rate (FDR). Genes with differential scores greater than 13.0 between *R-DCKO* samples and *cre-neg* littermate control samples (corresponding to *P*<0.05) are listed in Supplemental Tables 2 (down-regulated in *R-DCKO*) and 3 (up-regulated in *R-DCKO*). Cell process categories were determined by searching NCBI GENE (http://www.ncbi.nlm.nih.gov/gene), the Mouse Genome Informatics database (http://www.informatics.jax.org/), Sigma-Aldrich's “My Gene” search tool (http://www.sigmaaldrich.com/catalog/genes/), and Ingenuity Systems (http://www.ingenuity.com/).

#### Chromatin Immunoprecipitation (ChIP)

Was performed as previously described [84] [85]. Basically, 6 retinas per sample were dissected and chromatin was cross-linked with 1% formaldehyde in PBS for one minute at room temperature. After cell lysis and chromatin fragmentation by sonication, chromatin fragments were immunoprecipitated with antibodies against acetyl-histone H3 or acetyl-histone H4 bound to Protein A beads (GE Healthcare Life Sciences, Piscataway, NJ). After extensive washing, the immunoprecipitated chromatin was eluted with 50 mM NaHCO_3_/1% SDS, heated to 67°C to reverse the cross-links, the DNA purified by ethanol precipitation and analyzed by PCR with gene-specific primers (see references [5] [84] and [85] for sequences).

#### qRT-PCR and qChIP

Were performed as previously described [5] [86], in accordance with MIQE guidelines [87]. Primers were designed using MacVector software (MacVector, Inc., Cary, NC). RT-PCR were designed so that the product crosses at least one intron to prevent amplification of any residual genomic DNA. Optimal annealing temperatures and linearity of primer reactions were validated using dilutions of cDNA from control retinas, and primer pairs were only used if reaction efficiency fell between 90–110% and r^2^>0.980 [88]. Amplification of a single species was confirmed by melt curve analysis and agarose gel electrophoresis.

For qRT-PCR, RNA was extracted from two retinas per sample (∼30 mg tissue) using PerfectPure RNA cell & tissue extraction kit (5-Prime Inc., Gaithersburg, MD) and quantified on the NanoDrop. cDNA was synthesized from 1.0 µg RNA with oligo-dT primers, using the Transcriptor First Strand cDNA Synthesis Kit (Roche 04 379 012 001) according to the manufacturer's directions.

For both qRT-PCR and qChIP, 10 µl reactions were set up in triplicate in 96-well low-profile frosted PCR plates (Midsci, St. Louis, MO), with 2 µM primers (see references [5] [84] [85] [86] for sequences), using SYBR Green JumpStart Readymix (Sigma, St. Louis, MO), and run on a BioRad CFX thermocycler. The test protocol consists of 40 cycles of two-step amplification followed by melt curve analysis. For unknowns, only C_q_ values that fell within the linear range determined for each primer pair were used; samples giving results outside this range were diluted appropriately and re-tested. Relative expression levels were normalized to three reference genes (*ß-Actin [Actb], Glyceraldheyde-3-phosphate dehydrogenase, [GAPDH] and Ubiquitin B [UBB]*), which were determined to be highly stable, using qbasePLUS software (BioGazelle NV, Zwijnaarde, Belgium). The mean value and standard deviation (STDEV) were calculated, and statistical significance (p<0.05) was determined using unpaired two-tailed Student's t-test.

## Supporting Information

Figure S1 ***Cre*** **expression and validation of** ***Cbp*** **depletion in target cells.** **A.** GFP expression in *Rho-iCre+; mTmG* mice shows that *Cre* activity is detectable by Postnatal Day 5 (P5) in a few cells in the outer neuroblast layer (**ONBL**). By P7 most ONBL cells express GFP, and levels remain high in the outer nuclear layer (**ONL**) through adulthood. Scale bar  = 25 µm for all panels. **B.** GFP expression in *HRGP-Cre+; mTmG* mice begins near the optic nerve head around P5, extending outward to the retinal periphery over the next few days. By P7, there are GFP positive cells scattered throughout both the dorsal and ventral retina. As the retina matures, GFP-positive cells become localized to the outer edge of the ONL in both dorsal (**D**) and ventral (**V**) regions. The numbers of GFP-expressing cells are comparable in the dorsal and ventral regions through adulthood. Scale bar  = 20 µm for all panels. **C.** Immunolabeling of 4-week-old retina sections for CBP shows reactivity in all nuclei of *Cre-negative* control retinas (left panel), with rod nuclei in the ONL showing the characteristic peripheral nuclear distribution pattern. The retina from a *p300 CH* mouse (middle panel), in which both copies of *Cbp* have been conditionally knocked out by *Rho-iCre* expression, has lost much of this pattern, although reactivity can still be seen in cones along the outer edge of the ONL. Specific reactivity is also missing from the irregular ONL in *R-DCKO* mice (right panel). Because of the high background staining with this anti-CBP antibody, anti-p300 was used to verify *Cbp/p300* conditional knockout in the studies reported here.
(TIF)

Figure S2 **IHC for retinal cell type markers at P32. A & B.** Cone S-Opsin (**A**) and M-Opsin (**B**), localized to cone outer segments in *Cre neg* retinae, are seen associated with the few cells still expressing p300 in the outer nuclear layer (ONL) of *R-DCKO* retinae, often in the middle of rosettes. **C.** Vesicular glutamate transporter 1 (VGLUT1), found in pre-synaptic terminals, marks the outer (OPL) and inner (IPL) plexiform layers. Protein Kinase C-alpha (PKC-alpha) is expressed by rod on-bipolar cells in the inner nuclear layer (INL). VGLUT1 staining is severely decreased in the OPL of *R-DCKO* retinae but still seen in the IPL. Bipolar cell processes in these retinae extend into the ONL. **D.** Glutamine synthetase is expressed by Mueller glia. Although their orderly arrangement across the retina is disrupted in *R-DCKO* eyes, additional expression in ONL cells is not seen. **E.** Calbindin is expressed in horizontal cells in the INL, and syntaxin marks amacrine cell processes in the IPL of both *Cre neg* and *R-DCKO* retinae. **F.** Neurofilament NF200 is expressed in ganglion cell (GC), amacrine, and horizontal cell processes in both *Cre neg* and *R-DCKO* retinae. These findings led us to conclude that non-photoreceptor cells were present in apparently normal numbers and positions in *R-DCKO* retinae, and that the ONL cells in these retinae were not expressing markers of other lineages.
(TIF)

Figure S3 **IHC for markers of DNA damage and replication.** **A.** Histone H2A.X phosphorylated on Serine 139 (green) accumulates at sites of double-strand DNA breaks [89]. Elongating lens fiber cells undergoing nuclear lysis and endothelial cells outside the lens epithelium at P14 serve as positive controls (left panel). Very few H2A.X-positive retinal cells are seen in either control *Cre neg* (middle panel) or *R-DCKO* retinas (right panel) at this age. Sections are counter-stained with peanut agglutinin (PNA, red), which marks cone cell sheaths and other extracellular matrix landmarks. **B.** Ki-67 (green) is a nuclear proliferation antigen expressed in all stages of the cell cycle [90]. Proliferating cells in the lens germinal zone and ciliary body at P14 serve as positive controls (left panel). Rare positive cells are found within control or R-DCKO retinas at P14. Sections are counter-stained with PNA (red). **B.** Phosphorylation of histone H3 serine 10 (green) occurs during mitosis and is required for chromosome condensation [91]. Dividing cells in the lens germinal zone and ciliary body of P10 retinal sections serve as positive controls (left panel). Positive cells can be seen in the RPE of control and *R-DCKO* retinas at this age, but few positive cells are seen within the retina itself. **CB,** ciliary body; **ONL,** outer nuclear layer; **INL,** inner nuclear layer; **GC,** ganglion cell layer. Scale bars  = 25 µm for all panels.
(TIF)

Figure S4 **IHC for markers associated with neural or retinal precursor cells.** *Cre neg* and *R-DCKO* retinal sections were examined for markers reported to be associated with retinal precursors, to determine whether *R-DCKO* outer nuclear layer cells re-express early differentiation markers. **A.** The C-15 anti-Retinoblastoma antibody stains most nuclei in both *Cre-neg* control and *R-DCKO* sections. At P22, the staining pattern in ONL nuclei reflects the euchromatin distribution pattern. **B.** P15 ONL cells are negative for expression of Retinoblastoma-like 1/p107, which is expressed in embryonic mouse retina [76]. **C & D.** Nestin and NeuN are expressed in most developing neurons soon after withdrawal from the cell cycle. **C.** P15 sections express little Nestin (background fluorescence is associated with blood vessels). **D.** NeuN expression at P32 marks neurons in the INL and GC layers. **E & F.** Pax6 and Sox2 are highly expressed in proliferating retinal progenitors, but are also expressed in subsets of inner retina cells later during development [92] [93]. **E.** At P15, Pax6 immunoreactivity is seen in the INL and GC in both *Cre neg* and *R-DCKO* cells, but is absent from the ONL. **F.** Rare Sox2-positive cells are seen in the GC layer of P15 retinae, with diffuse reactivity evident in the ONL of both genotypes. **G.** The nuclear protein Geminin is a dual-function molecule that is involved in marking DNA during replication, and in controlling fate choice during neural development [94] [95]. **H.** The Tuj1 monoclonal antibody recognizes a class III beta-tubulin epitope that is expressed early in differentiation of vertebrate neurons. It strongly recognizes ganglion cells and their fibers, and is weakly expressed in some INL cells at P32, but is not expressed by *R-DCKO* ONL cells. These findings led us to conclude that loss of *Ep300/Cbp* did not lead rod cells to adopt a more primitive cell fate.
(TIF)

Figure S5 **qRT-PCR & qChIP supplemental data.** **A.** Comparison of gene expression in P14 *Rho-iCre* conditional knockout (CKO) retinas, by qRT-PCR: The rod gene *phosphodiesterase 6b (* ***Pde6b*** *)*; cone *M-opsin* ***(Opn1MW*** *)* and *S-opsin (* ***Opn1SW*** *)*; *interphotoreceptor retinoid binding protein (* ***Rbp3*** *)* expressed in both rods and cones; HAT-containing coactivators ***Cbp***, ***p300***, and ***Gcn5***; and the bipolar gene *metabotropic glutamate receptor type 6 (* ***Grm6*** *)*. Since cDNA was made from RNA isolated from whole retinas, *Cbp* and *p300* expression were detected in CKO retinas due to expression in unaffected inner retina cell types. In contrast, expression of *Gcn5* was high in CKO retinas, suggesting that knockout of *Ep300/Cbp* does not abolish all transcription in rods. **B.** qChIP assays for acetylated histone H3 (**AcH3**) and H4 (**AcH4**) occupancy on the promoters of *interphotoreceptor retinoid binding protein (* ***Rbp3*** *),* cone *M-opsin (* ***Opn1mw*** *)* and *S-opsin (* ***Opn1sw*** *)*, and *Grm6*, expressed by bipolar cells. In contrast to the severely affected rod genes in the conditional knockouts (Fig. 5C–F), these controls show that occupancy is preserved for cone genes whose expression is unaffected, for photoreceptor genes that are CRX-independent (*RBP3*), and for genes are expressed in other cell types (*Grm6*) in the samples tested for Figure 5. **C.** Gel images of ChIP-PCR results for AcH3 promoter occupancy in control and CKO retinas. For each sample type, the first lane (**IP**) is from immunoprecipitated samples; the second (**IgG**) from “no antibody” negative controls, the third (**noDNA**) from “no DNA” control reactions, and the fourth lane (**Input**) is nuclear lysate prior to immunoprecipitation. In CKO samples, all CRX-dependent gene promoters tested lost AcH3 occupancy, confirming the qChIP results. **M**, 100-bp DNA molecular weight ladder.
(TIF)

Table S1 **Genotyping primers.**
(DOCX)

Table S2 **520 genes down-regulated in** ***R-DCKO*** **vs** ***Cre negative.***
(DOCX)

Table S3 **520 genes down-regulated in** ***R-DCKO*** **vs** ***Cre negative.***
(DOCX)

Table S4 **p300/CBP dependent genes linked to retinal disease.**
(DOCX)

Table S5 **p300/CBP dependent genes downregulated in** ***Crx^−/−^_._***
(DOCX)

Table S6 **ERG Test Parameters.**
(DOCX)

Table S7 **Antibodies.**
(DOCX)
