# Supplementary figures and images for: Transcription Coactivators p300 and CBP Are Necessary for Photoreceptor-Specific Chromatin Organization and Gene Expression
Source: PLoS One. 2013 Jul 26;8(7):e69721. doi: 10.1371/journal.pone.0069721 (PMC3724885; doi:10.1371/journal.pone.0069721)

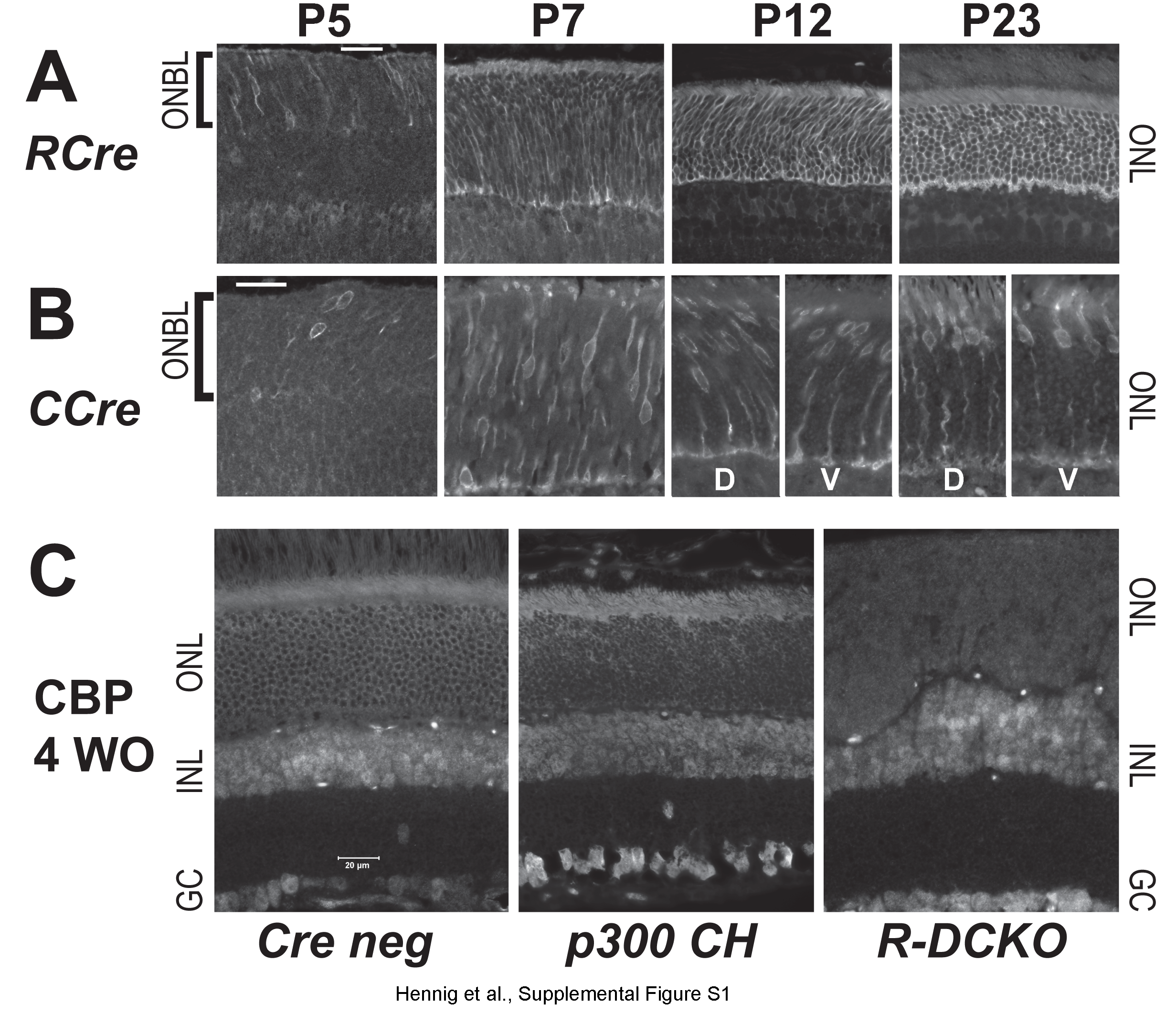

Supplement: Figure S1 — Cre expression and validation of Cbp depletion in target cells. A. GFP expression in Rho-iCre+; mTmG mice shows that Cre activity is detectable by Postnatal Day 5 (P5) in a few cells in the outer neuroblast layer (ONBL). By P7 most ONBL cells express GFP, and levels remain high in the outer nuclear layer (ONL) through adulthood. Scale bar = 25 µm for all panels. B. GFP expression in HRGP-Cre+; mTmG mice begins near the optic nerve head around P5, extending outward to the retinal periphery over the next few days. By P7, there are GFP positive cells scattered throughout both the dorsal and ventral retina. As the retina matures, GFP-positive cells become localized to the outer edge of the ONL in both dorsal (D) and ventral (V) regions. The numbers of GFP-expressing cells are comparable in the dorsal and ventral regions through adulthood. Scale bar = 20 µm for all panels. C. Immunolabeling of 4-week-old retina sections for CBP shows reactivity in all nuclei of Cre-negative control retinas (left panel), with rod nuclei in the ONL showing the characteristic peripheral nuclear distribution pattern. The retina from a p300 CH mouse (middle panel), in which both copies of Cbp have been conditionally knocked out by Rho-iCre expression, has lost much of this pattern, although reactivity can still be seen in cones along the outer edge of the ONL. Specific reactivity is also missing from the irregular ONL in R-DCKO mice (right panel). Because of the high background staining with this anti-CBP antibody, anti-p300 was used to verify Cbp/p300 conditional knockout in the studies reported here. (TIF) [file pone.0069721.s001.tif]

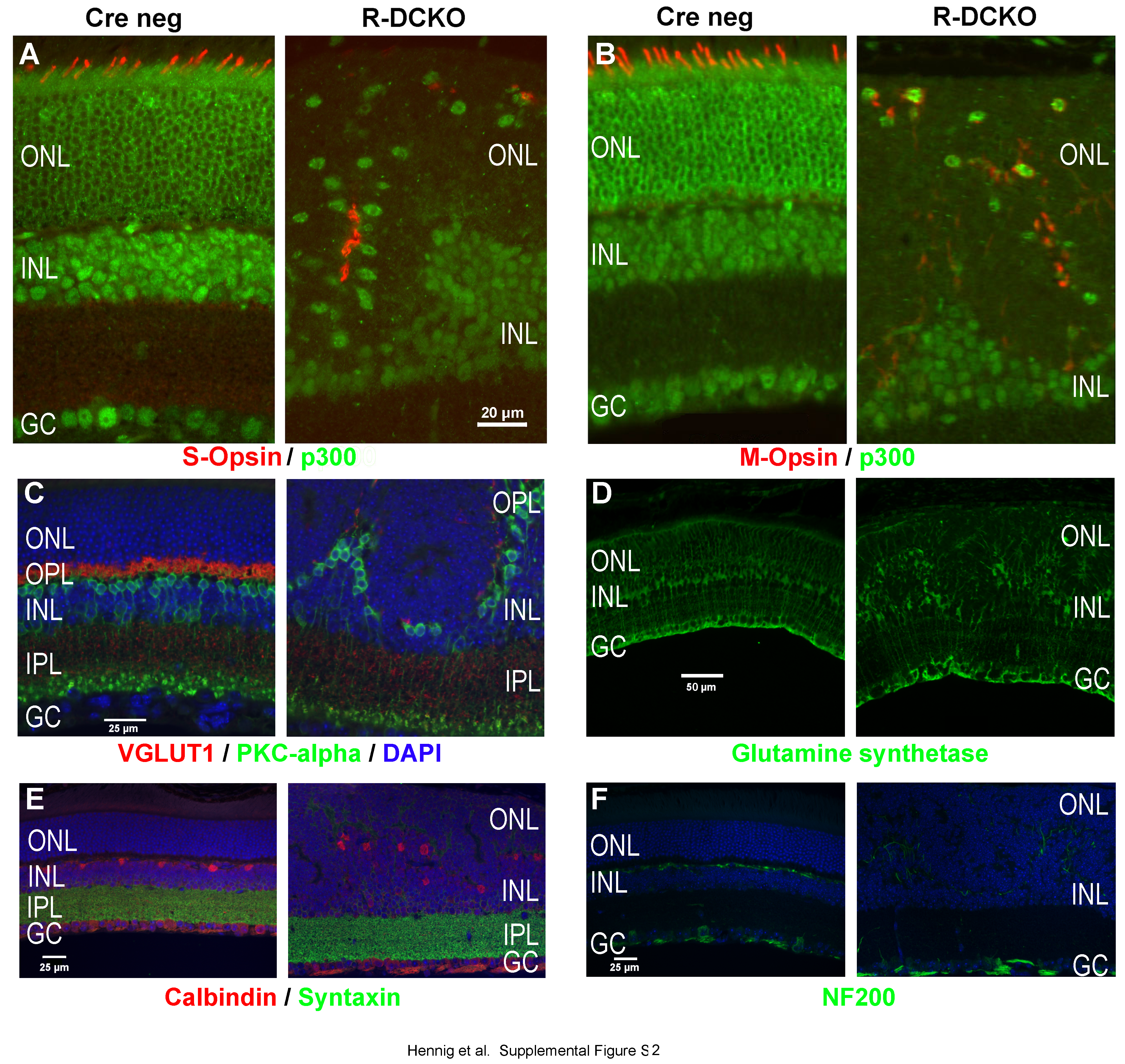

Supplement: Figure S2 — IHC for retinal cell type markers at P32. A & B. Cone S-Opsin (A) and M-Opsin (B), localized to cone outer segments in Cre neg retinae, are seen associated with the few cells still expressing p300 in the outer nuclear layer (ONL) of R-DCKO retinae, often in the middle of rosettes. C. Vesicular glutamate transporter 1 (VGLUT1), found in pre-synaptic terminals, marks the outer (OPL) and inner (IPL) plexiform layers. Protein Kinase C-alpha (PKC-alpha) is expressed by rod on-bipolar cells in the inner nuclear layer (INL). VGLUT1 staining is severely decreased in the OPL of R-DCKO retinae but still seen in the IPL. Bipolar cell processes in these retinae extend into the ONL. D. Glutamine synthetase is expressed by Mueller glia. Although their orderly arrangement across the retina is disrupted in R-DCKO eyes, additional expression in ONL cells is not seen. E. Calbindin is expressed in horizontal cells in the INL, and syntaxin marks amacrine cell processes in the IPL of both Cre neg and R-DCKO retinae. F. Neurofilament NF200 is expressed in ganglion cell (GC), amacrine, and horizontal cell processes in both Cre neg and R-DCKO retinae. These findings led us to conclude that non-photoreceptor cells were present in apparently normal numbers and positions in R-DCKO retinae, and that the ONL cells in these retinae were not expressing markers of other lineages. (TIF) [file pone.0069721.s002.tif]

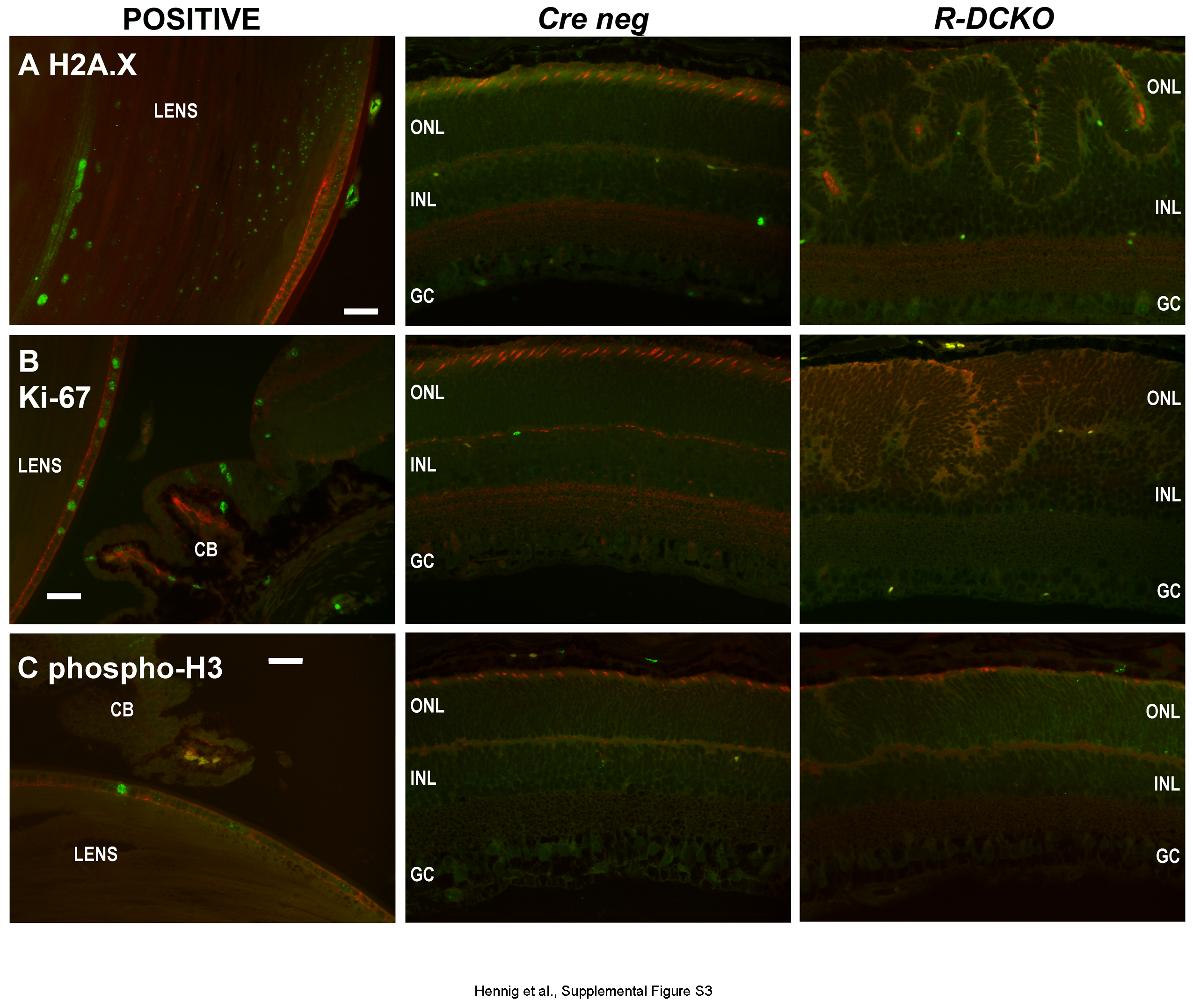

Supplement: Figure S3 — IHC for markers of DNA damage and replication. A. Histone H2A.X phosphorylated on Serine 139 (green) accumulates at sites of double-strand DNA breaks [89]. Elongating lens fiber cells undergoing nuclear lysis and endothelial cells outside the lens epithelium at P14 serve as positive controls (left panel). Very few H2A.X-positive retinal cells are seen in either control Cre neg (middle panel) or R-DCKO retinas (right panel) at this age. Sections are counter-stained with peanut agglutinin (PNA, red), which marks cone cell sheaths and other extracellular matrix landmarks. B. Ki-67 (green) is a nuclear proliferation antigen expressed in all stages of the cell cycle [90]. Proliferating cells in the lens germinal zone and ciliary body at P14 serve as positive controls (left panel). Rare positive cells are found within control or R-DCKO retinas at P14. Sections are counter-stained with PNA (red). B. Phosphorylation of histone H3 serine 10 (green) occurs during mitosis and is required for chromosome condensation [91]. Dividing cells in the lens germinal zone and ciliary body of P10 retinal sections serve as positive controls (left panel). Positive cells can be seen in the RPE of control and R-DCKO retinas at this age, but few positive cells are seen within the retina itself. CB, ciliary body; ONL, outer nuclear layer; INL, inner nuclear layer; GC, ganglion cell layer. Scale bars = 25 µm for all panels. (TIF) [file pone.0069721.s003.tif]

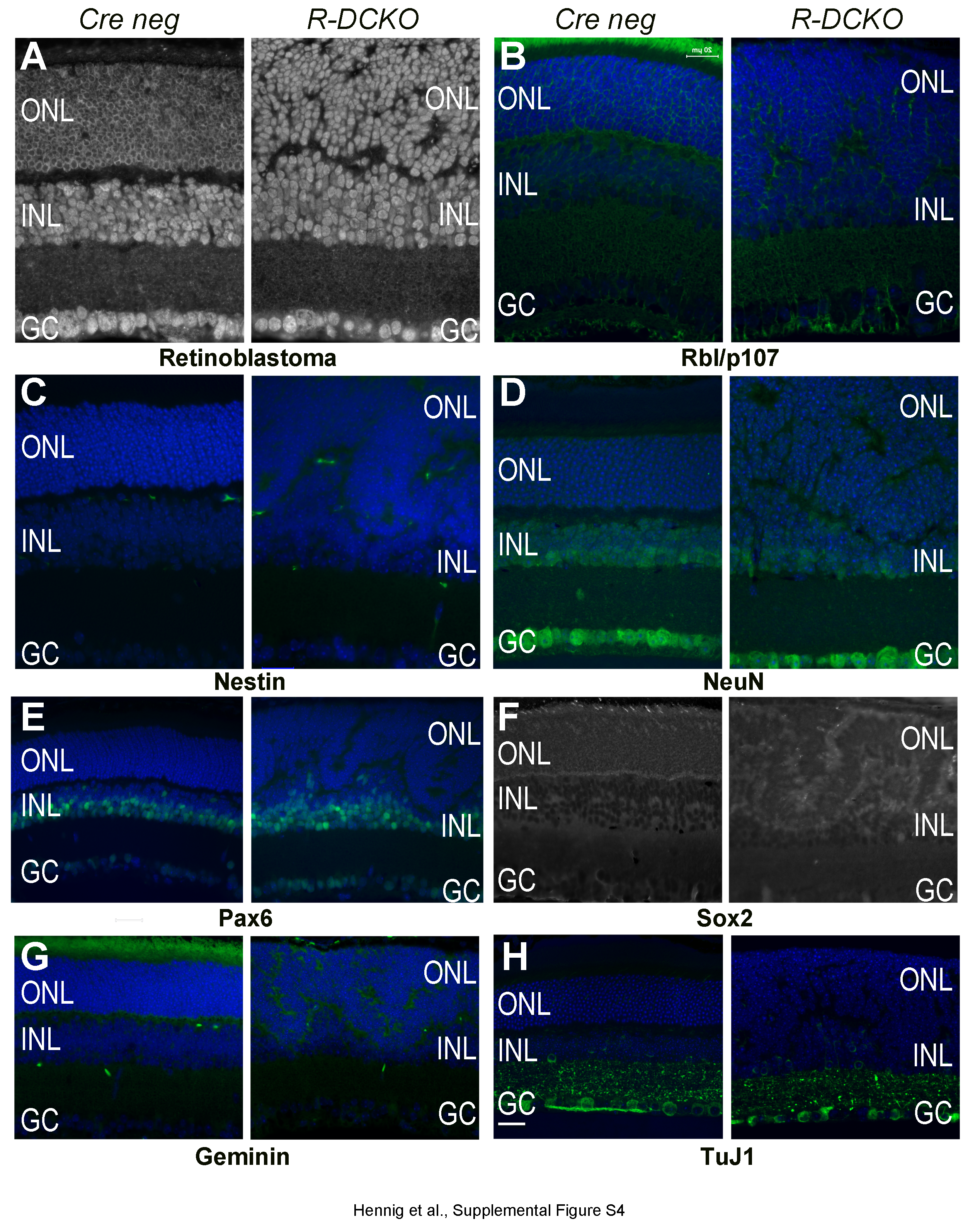

Supplement: Figure S4 — IHC for markers associated with neural or retinal precursor cells. Cre neg and R-DCKO retinal sections were examined for markers reported to be associated with retinal precursors, to determine whether R-DCKO outer nuclear layer cells re-express early differentiation markers. A. The C-15 anti-Retinoblastoma antibody stains most nuclei in both Cre-neg control and R-DCKO sections. At P22, the staining pattern in ONL nuclei reflects the euchromatin distribution pattern. B. P15 ONL cells are negative for expression of Retinoblastoma-like 1/p107, which is expressed in embryonic mouse retina [76]. C & D. Nestin and NeuN are expressed in most developing neurons soon after withdrawal from the cell cycle. C. P15 sections express little Nestin (background fluorescence is associated with blood vessels). D. NeuN expression at P32 marks neurons in the INL and GC layers. E & F. Pax6 and Sox2 are highly expressed in proliferating retinal progenitors, but are also expressed in subsets of inner retina cells later during development [92] [93]. E. At P15, Pax6 immunoreactivity is seen in the INL and GC in both Cre neg and R-DCKO cells, but is absent from the ONL. F. Rare Sox2-positive cells are seen in the GC layer of P15 retinae, with diffuse reactivity evident in the ONL of both genotypes. G. The nuclear protein Geminin is a dual-function molecule that is involved in marking DNA during replication, and in controlling fate choice during neural development [94] [95]. H. The Tuj1 monoclonal antibody recognizes a class III beta-tubulin epitope that is expressed early in differentiation of vertebrate neurons. It strongly recognizes ganglion cells and their fibers, and is weakly expressed in some INL cells at P32, but is not expressed by R-DCKO ONL cells. These findings led us to conclude that loss of Ep300/Cbp did not lead rod cells to adopt a more primitive cell fate. (TIF) [file pone.0069721.s004.tif]

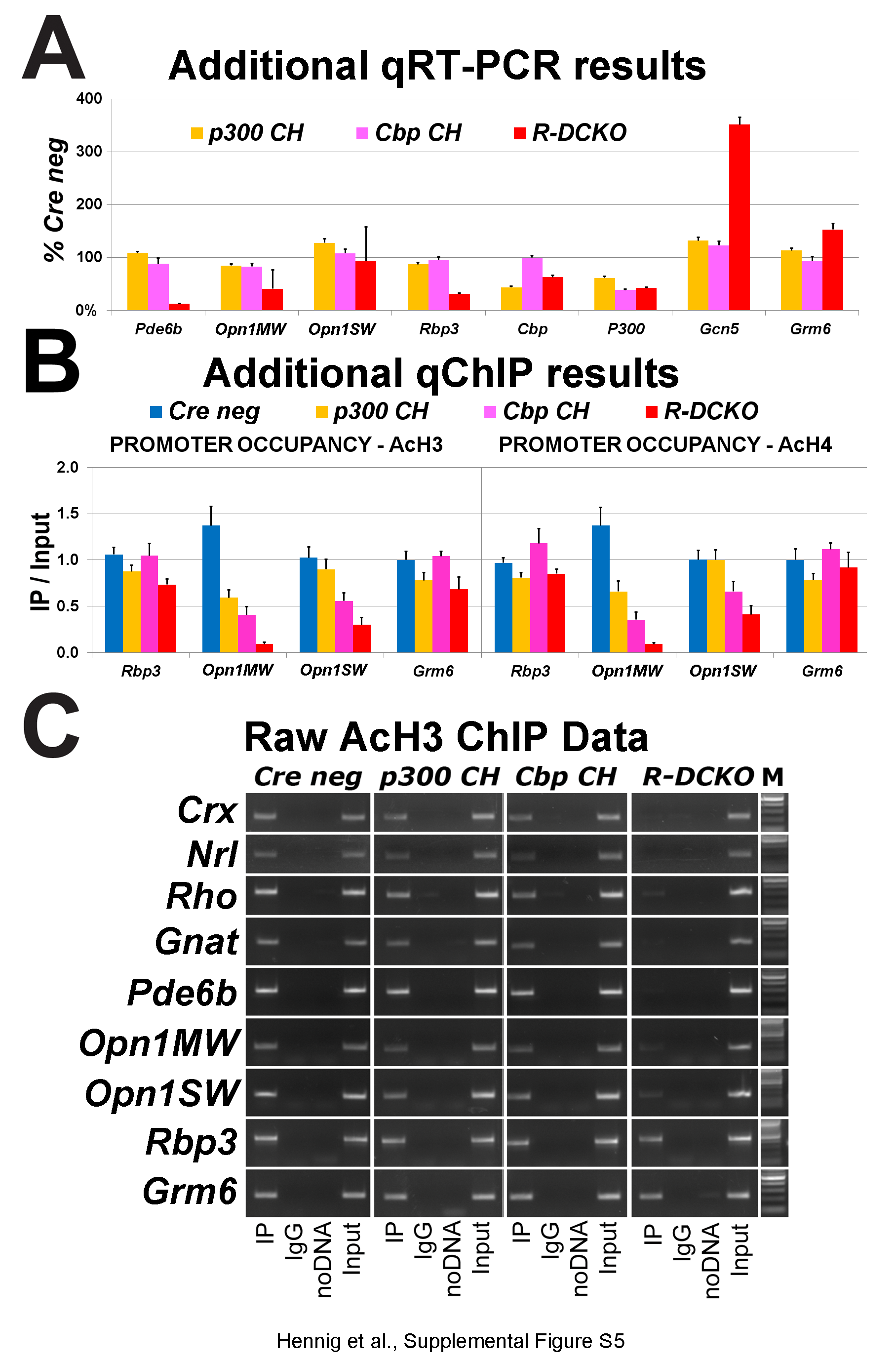

Supplement: Figure S5 — qRT-PCR & qChIP supplemental data. A. Comparison of gene expression in P14 Rho-iCre conditional knockout (CKO) retinas, by qRT-PCR: The rod gene phosphodiesterase 6b ( Pde6b ); cone M-opsin (Opn1MW ) and S-opsin ( Opn1SW ); interphotoreceptor retinoid binding protein ( Rbp3 ) expressed in both rods and cones; HAT-containing coactivators Cbp, p300, and Gcn5; and the bipolar gene metabotropic glutamate receptor type 6 ( Grm6 ). Since cDNA was made from RNA isolated from whole retinas, Cbp and p300 expression were detected in CKO retinas due to expression in unaffected inner retina cell types. In contrast, expression of Gcn5 was high in CKO retinas, suggesting that knockout of Ep300/Cbp does not abolish all transcription in rods. B. qChIP assays for acetylated histone H3 (AcH3) and H4 (AcH4) occupancy on the promoters of interphotoreceptor retinoid binding protein ( Rbp3 ), cone M-opsin ( Opn1mw ) and S-opsin ( Opn1sw ), and Grm6, expressed by bipolar cells. In contrast to the severely affected rod genes in the conditional knockouts (Fig. 5C–F), these controls show that occupancy is preserved for cone genes whose expression is unaffected, for photoreceptor genes that are CRX-independent (RBP3), and for genes are expressed in other cell types (Grm6) in the samples tested for Figure 5. C. Gel images of ChIP-PCR results for AcH3 promoter occupancy in control and CKO retinas. For each sample type, the first lane (IP) is from immunoprecipitated samples; the second (IgG) from “no antibody” negative controls, the third (noDNA) from “no DNA” control reactions, and the fourth lane (Input) is nuclear lysate prior to immunoprecipitation. In CKO samples, all CRX-dependent gene promoters tested lost AcH3 occupancy, confirming the qChIP results. M, 100-bp DNA molecular weight ladder. (TIF) [file pone.0069721.s005.tif]
