# Supplementary material for: Transcription Coactivators p300 and CBP Are Necessary for Photoreceptor-Specific Chromatin Organization and Gene Expression
Source: PLoS One. 2013 Jul 26;8(7):e69721. doi: 10.1371/journal.pone.0069721 (PMC3724885; doi:10.1371/journal.pone.0069721)
Supplement: Table S1 — Genotyping primers. (DOCX) [file pone.0069721.s006.docx]

| **Table S1. Genotyping primers** | | |  |  |  |  |
| --- | --- | --- | --- | --- | --- | --- |
| **NAME** | **GENE** | **F PRIMER** | **R PRIMER** | **BAND SIZES** | **TEXT REF #** |  |
| p300 Flox | p300 | 5'-TGGACTGGTTATCGGTTCACC | 5'-CAGTTACATACAGCTGTGATG-3' | Flox = 1000 bp WT = 800 bp | 19 | |
| Cbp Flox | Cbp | 5'-CCTGGTTGCCTATGCTAAGAAAG | 5'-CTGCTCTACCTAAATTCCCAG | Flox = 800 bp WT = 650 bp | 39 | |
| Rho-iCre | Cre (rods) | 5’-TCAGTGCCTGGAGTTGCGCTGTGG | 5’-TTCAAAGGCCAGGGCCTGCTTGGC | 650 bp | 45 | |
| HRGP-Cre | Cre (cones) | 5’-GGAGGATCCGGTTCCAGGCC | 5’-CGACGATGAAGCATGTTTAGCTGG | 600-700 bp | 46 | |
| mT/mG | GFP | 5'-CTCTGCTGCCTCCTGGCTTCT | 5'-CGAGGCGGATCACAAGCAATA | WT = 330 | 47 | |
|  |  | 5'-CTCTGCTGCCTCCTGGCTTCT | 5'-TCAATGGGCGGGGGTCGTT | mTmG = 250 |  |  |
| RD1 | Pde6b mutation | 5' -TGACAATTACTCCTTTTCCCTCAGTCTG | 5'-GTAAACAGCAAGAGGCTTTATTGGGAAC | rd1 = 550 bp | 81 | |
|  |  | 5' -TGACAATTACTCCTTTTCCCTCAGTCTG | 5'-TACCCACCCTTCCTAATTTTTCTCACGC | WT = 400 bp |  |  |
| RD8 | Crb mutation | 5'-GTGAAGACAGCTACAGTTCTGATC | 5'-GCCCCATTTGCACACTGATGAC | WT = 220bp | 82 | |
|  |  | 5'-GCCCCTGTTTGCATGGAGGAAACTTGG-AAGACAGCTACAGTTCTTCTG | 5'-GCCCCATTTGCACACTGATGAC | rd8 = 244bp |  |  |
