# Supplementary material for: Transcription Coactivators p300 and CBP Are Necessary for Photoreceptor-Specific Chromatin Organization and Gene Expression
Source: PLoS One. 2013 Jul 26;8(7):e69721. doi: 10.1371/journal.pone.0069721 (PMC3724885; doi:10.1371/journal.pone.0069721)
Supplement: Table S2 — 520 genes down-regulated in R-DCKO vs Cre negative. (DOCX) [file pone.0069721.s007.docx]

| **Table S2. 520 genes down-regulated in *R-DCKO* vs *Cre negative*** | | | | | | |  | |  | |  | |
| --- | --- | --- | --- | --- | --- | --- | --- | --- | --- | --- | --- | --- |
| **SYMBOL** | **ILMN_GENE** | **CHROMOSOME** | | **DEFINITION** | | **R-DCKO DIFF SCORE** | | **% CRE NEG** | | **CELL PROCESS** | |  |
| Guca1b | GUCA1B | | 17 | | Mus musculus guanylate cyclase activator 1B (Guca1b), mRNA. | | -232.73 | | 2.7 | | PHOTOTRANSDUCTION | |
| Drd4 | DRD4 | | 7 | | Mus musculus dopamine receptor 4 (Drd4), mRNA. | | -199.06 | | 9.1 | | SYNAPTIC/SECRETORY FUNCTION | |
| Vtn | VTN | | 11 | | Mus musculus vitronectin (Vtn), mRNA. | | -194.01 | | 8.6 | | CELL ADHESION | |
| Cabp4 | CABP4 | | 19 | | Mus musculus calcium binding protein 4 (Cabp4), mRNA. | | -189.84 | | 10.0 | | PHOTOTRANSDUCTION | |
| Rho | RHO | | 6 | | Mus musculus rhodopsin (Rho), mRNA. | | -189.49 | | 8.2 | | PHOTOTRANSDUCTION | |
| LOC100047468 | LOC100047468 | | 8 | | PREDICTED: Mus musculus similar to Thioredoxin-like 6 (LOC100047468), mRNA. | | -176.07 | | 3.8 | |  | |
| Dohh | 1110033C18RIK | | 10 | | Mus musculus deoxyhypusine hydroxylase/monooxygenase | | -171.81 | | 12.3 | | METABOLISM, MITO FXN | |
| Glb1l2 | GLB1L2 | | 9 | | Mus musculus galactosidase, beta 1-like 2 (Glb1l2), mRNA. | | -171.04 | | 14.1 | | METABOLISM, MITO FXN | |
| A930034L24Rik | A930034L24RIK | |  | |  | | -168.18 | | 8.6 | | UNKNOWN GENE | |
| Ppef2 | PPEF2 | | 5 | | Mus musculus protein phosphatase, EF hand calcium-binding domain 2 (Ppef2), mRNA. | | -167.47 | | 12.8 | | PHOTOTRANSDUCTION | |
| Fam57b | 1500016O10RIK | | 7 | | PREDICTED: Mus musculus family with sequence similarity 57, member B | | -164.49 | | 13.1 | | UNKNOWN FUNCTION | |
| Gnb1 | GNB1 | | 4 | | Mus musculus guanine nucleotide binding protein (G protein), beta 1 (Gnb1), mRNA. | | -157.85 | | 19.2 | | PHOTOTRANSDUCTION | |
| Ppargc1b | PPARGC1B | | 18 | | Mus musculus peroxisome proliferative activated receptor, gamma, coactivator 1 beta (Ppargc1b), mRNA. | | -156.95 | | 16.1 | | METABOLISM, MITO FXN | |
| LOC380890 | LOC380890 | |  | |  | | -150.60 | | 6.3 | | UNKNOWN GENE | |
| Rdh12 | RDH12 | | 12 | | Mus musculus retinol dehydrogenase 12 | | -140.87 | | 13.9 | | PHOTOTRANSDUCTION | |
| Pde6b | PDE6B | | 5 | | Mus musculus phosphodiesterase 6B, cGMP, rod receptor, beta polypeptide | | -140.13 | | 13.6 | | PHOTOTRANSDUCTION | |
| Slc17a7 | SLC17A7 | | 7 | | Mus musculus solute carrier family 17 (sodium-dependent inorganic phosphate cotransporter), member 7 (Slc17a7), mRNA. | | -138.89 | | 22.1 | | NEURON FUNCTION | |
| Kcnv2 | KCNV2 | | 19 | | Mus musculus potassium channel, subfamily V, member 2 (Kcnv2), mRNA. | | -135.73 | | 16.7 | | INTRACELLULAR TRANSPORT | |
| Igsf9 | IGSF9 | | 1 | | Mus musculus immunoglobulin superfamily, member 9 (Igsf9), mRNA. | | -134.84 | | 16.1 | | SYNAPTIC/SECRETORY FUNCTION | |
| Akp2 | AKP2 | | 4 | | Mus musculus alkaline phosphatase 2, liver (Akp2), mRNA. | | -134.63 | | 15.2 | | METABOLISM, MITO FXN | |
| scl0004166.1_8 | SCL0004166.1_8 | |  | |  | | -128.08 | | 17.5 | | UNKNOWN GENE | |
| Ano2 | TMEM16B | | 6 | | Mus musculus anoctamin2 (transmembrane protein 16B (Tmem16b), mRNA. | | -128.02 | | 18.4 | | PHOTORECEPTOR GENE | |
| Unc119 | UNC119 | | 11 | | Mus musculus unc-119 homolog (C. elegans) (Unc119), mRNA. XM_001004318 | | -125.03 | | 23.4 | | PHOTOTRANSDUCTION | |
| Cngb1 | BC016201 | | 8 | | Mus musculus cDNA sequence BC016201 (BC016201), cyclic nucleotide gated channel beta 1, mRNA. | | -123.23 | | 16.3 | | PHOTOTRANSDUCTION | |
| A930004D18Rik | A930004D18RIK | | 2 | | Mus musculus RIKEN cDNA A930004D18 gene | | -122.64 | | 8.7 | | UNKNOWN FUNCTION | |
| Fabp12 | 1700008G05RIK | | 3 | | Mus musculus fatty acid binding protein 12 | | -122.13 | | 13.0 | | INTRACELLULAR TRANSPORT | |
| BC027072 | BC027072 | | 17 | | Mus musculus cDNA sequence BC027072 (BC027072), mRNA homolog of human C2ORF71. | | -121.30 | | 13.7 | | PHOTOTRANSDUCTION | |
| Cplx4 | CPLX4 | | 18 | | Mus musculus complexin 4 (Cplx4), mRNA. | | -119.48 | | 13.0 | | SYNAPTIC/SECRETORY FUNCTION | |
| Susd3 | SUSD3 | | 13 | | Mus musculus sushi domain containing 3 (Susd3), mRNA. | | -119.06 | | 11.0 | | UNKNOWN FUNCTION | |
| Fscn2 | FSCN2 | | 11 | | Mus musculus fascin homolog 2, actin-bundling protein, retinal (Strongylocentrotus purpuratus) (Fscn2), mRNA. | | -118.84 | | 3.1 | | PHOTORECEPTOR GENE | |
| Plcd3 | PLCD3 | | 11 | | Mus musculus phospholipase C, delta 3 (Plcd3), mRNA. | | -117.89 | | 20.5 | | METABOLISM, MITO FXN | |
| Rp1l1 | RP1L1 | | 14 | | Mus musculus retinitis pigmentosa 1 homolog (human)-like 1 (Rp1l1), mRNA. | | -116.53 | | 13.0 | | PHOTORECEPTOR GENE | |
| Ankrd33 | ANKRD33 | | 15 | | Mus musculus ankyrin repeat domain 33 (Ankrd33), mRNA. | | -116.36 | | 18.2 | | UNKNOWN FUNCTION | |
| Cacna2d4 | CACNA2D4 | | 6 | | Mus musculus calcium channel, voltage-dependent, alpha 2/delta subunit 4 (Cacna2d4), mRNA. | | -115.60 | | 8.8 | | PHOTORECEPTOR GENE | |
| Ccdc126 | CCDC126 | | 6 | | Mus musculus coiled-coil domain containing 126 (Ccdc126), mRNA. | | -114.73 | | 29.5 | | UNKNOWN FUNCTION | |
| Psd2 | PSD2 | | 18 | | Mus musculus pleckstrin and Sec7 domain containing 2 (Psd2), mRNA. | | -113.15 | | 27.7 | | SYNAPTIC/SECRETORY FUNCTION | |
| Slc38a3 | SLC38A3 | | 9 | | Mus musculus solute carrier family 38, member 3 (Slc38a3), mRNA. | | -112.36 | | 28.0 | | TRANSMEMBRANE TRANSPORT | |
| Rom1 | ROM1 | | 19 | | Mus musculus rod outer segment membrane protein 1 (Rom1), mRNA. | | -111.86 | | 18.6 | | PHOTORECEPTOR GENE | |
| A930010M14Rik | A930010M14RIK | |  | |  | | -111.02 | | 11.3 | | UNKNOWN GENE | |
| 2610034M16Rik | 2610034M16RIK | | 17 | | Mus musculus RIKEN cDNA 2610034M16 gene (2610034M16Rik), mRNA. XM_925124 XM_980421 XM_980454 XM_980485 | | -110.73 | | 3.2 | | UNKNOWN FUNCTION | |
| Mak | MAK | | 13 | | Mus musculus male germ cell-associated kinase (Mak), mRNA. | | -109.12 | | 22.0 | | PHOTORECEPTOR GENE | |
| Nt5e | NT5E | | 9 | | Mus musculus 5' nucleotidase, ecto (Nt5e), mRNA. | | -107.37 | | 13.9 | | METABOLISM, MITO FXN | |
| 4930405J17Rik | 4930405J17RIK | | 10 | | Mus musculus RIKEN cDNA 4930405J17 ge | | -105.33 | | 3.4 | | UNKNOWN FUNCTION | |
| A930011O08Rik | A930011O08RIK | |  | |  | | -105.33 | | 20.5 | | UNKNOWN GENE | |
| LOC546006 | LOC546006 | | 7 | | PREDICTED: Mus musculus similar to deleted in malignant brain tumors 1 (LOC546006), mRNA. | | -104.03 | | 19.5 | | UNKNOWN FUNCTION | |
| B230312E02Rik | B230312E02RIK | |  | |  | | -104.01 | | 24.7 | | UNKNOWN GENE | |
| Serinc4 | SERINC4 | | 2 | | Mus musculus serine incorporator 4 (Serinc4), mRNA. | | -103.62 | | 12.6 | | METABOLISM, MITO FXN | |
| Grk1 | GRK1 | | 8 | | Mus musculus G protein-coupled receptor kinase 1 (Grk1), mRNA. | | -102.56 | | 10.2 | | INTRACELLULAR SIGNAL TRANSDUCTION | |
| Tnfsf12-tnfsf13 | TNFSF12-TNFSF13 | | 11 | | Mus musculus tumor necrosis factor (ligand) superfamily, member 12-member 13 (Tnfsf12-tnfsf13), transcript variant 1, mRNA. | | -100.25 | | 15.2 | | CELL-CELL COMMUNICATION | |
| Mylk | MYLK | | 16 | | Mus musculus myosin, light polypeptide kinase (Mylk), mRNA. | | -100.23 | | 17.4 | | INTRACELLULAR TRANSPORT | |
| Ppap2c | PPAP2C | | 10 | | Mus musculus phosphatidic acid phosphatase type 2C | | -99.81 | | 18.1 | | METABOLISM, MITO FXN | |
| Slc4a7 | SLC4A7 | | 14 | | Mus musculus solute carrier family 4, sodium bicarbonate cotransporter, member 7 | | -97.94 | | 23.0 | | PHOTORECEPTOR GENE | |
| Slco4a1 | SLCO4A1 | | 2 | | Mus musculus solute carrier organic anion transporter family, member 4a1 (Slco4a1), mRNA. | | -97.08 | | 8.2 | | TRANSMEMBRANE TRANSPORT | |
| Rrp1b | 2600005C20RIK | | 17 | | Mus musculus ribosomal RNA processing 1 homolog B (S. cerevisiae) | | -96.40 | | 33.1 | | TRANSCRIPTION/TRANSLATION | |
| Plch2 | PLCH2 | | 4 | | Mus musculus phospholipase C, eta 2 (Plch2), transcript variant 1, mRNA. | | -95.84 | | 18.9 | | METABOLISM, MITO FXN | |
| Pde6g | PDE6G | | 11 | | Mus musculus phosphodiesterase 6G, cGMP-specific, rod, gamma (Pde6g), mRNA. | | -94.11 | | 24.7 | | PHOTOTRANSDUCTION | |
| Mpp4 | MPP4 | | 1 | | Mus musculus membrane protein, palmitoylated 4 (MAGUK p55 subfamily member 4) (Mpp4), mRNA. | | -93.11 | | 13.8 | | PHOTORECEPTOR GENE | |
| Rtbdn | RTBDN | | 8 | | Mus musculus retbindin (Rtbdn), mRNA. | | -92.89 | | 20.6 | | PHOTOTRANSDUCTION | |
| Cdr2 | CDR2 | | 7 | | Mus musculus cerebellar degeneration-related 2 (Cdr2), mRNA. | | -90.51 | | 10.8 | | UNKNOWN FUNCTION | |
| Snta1 | SNTA1 | | 2 | | Mus musculus syntrophin, acidic 1 (Snta1), mRNA. | | -90.39 | | 27.0 | | CELL ADHESION | |
| Kcnb1 | KCNB1 | | 2 | | Mus musculus potassium voltage gated channel, Shab-related subfamily, member 1 (Kcnb1), mRNA. | | -89.81 | | 30.2 | | SYNAPTIC/SECRETORY FUNCTION | |
| Aqp1 | AQP1 | | 6 | | Mus musculus aquaporin 1 (Aqp1), mRNA. | | -88.74 | | 8.0 | | METABOLISM, MITO FXN | |
| Gm514 | LOC208080 | | 9 | | PREDICTED: Mus musculus predicted gene 514 | | -85.60 | | 17.4 | | UNKNOWN FUNCTION | |
| Nxnl2 | 4930519N16RIK | | 13 | | Nucleoredoxin-like 2 [Hum Mol Genet. 2012 May 15;21(10):2298-311. Epub 2012 Feb 15] ; Crx response element; may be related to RDCVF [BMC Mol Biol 2007 8:74 doi:10.1186/1471-2199-8-74] - REF 56 Lambard! | | -84.72 | | 11.2 | | PHOTORECEPTOR GENE | |
| Fbxo27 | FBXO27 | | 7 | | Mus musculus F-box protein 27 (Fbxo27), mRNA. | | -84.58 | | 33.4 | | METABOLISM, MITO FXN | |
| Aipl1 | AIPL1 | | 11 | | Mus musculus aryl hydrocarbon receptor-interacting protein-like 1 | | -84.33 | | 17.5 | | PHOTOTRANSDUCTION | |
| LOC100047738 | LOC100047738 | |  | | PREDICTED: Mus musculus similar to DENN/MADD domain containing 1A (LOC100047738), mRNA. | | -84.01 | | 26.1 | | OTHER | |
| Slc16a6 | SLC16A6 | | 11 | | Mus musculus solute carrier family 16 (monocarboxylic acid transporters), member 6 (Slc16a6), transcript variant 2, mRNA. | | -83.84 | | 13.8 | | TRANSMEMBRANE TRANSPORT | |
| C330036H15Rik | C330036H15RIK | |  | |  | | -83.71 | | 37.4 | | UNKNOWN GENE | |
| Lrrc8 | LRRC8 | | 2 | | Mus musculus leucine rich repeat containing 8A | | -83.11 | | 26.0 | | METABOLISM, MITO FXN | |
| Taok3 | TAOK3 | | 5 | | Mus musculus TAO kinase 3 (Taok3), mRNA. | | -82.86 | | 16.1 | | DNA DAMAGE/REPAIR | |
| Samd11 | SAMD11 | | 4 | | Mus musculus sterile alpha motif domain containing 11 (Samd11), mRNA. | | -81.60 | | 11.9 | | PHOTORECEPTOR GENE | |
| Cds1 | CDS1 | | 5 | | Mus musculus CDP-diacylglycerol synthase 1 (Cds1), mRNA. | | -81.29 | | 15.3 | | METABOLISM, MITO FXN | |
| Taok3 | A130052D22 | | 5 | | Mus musculus TAO kinase 3 (Taok3), mRNA. | | -81.19 | | 16.8 | | DNA DAMAGE/REPAIR | |
| Tulp1 | TULP1 | | 17 | | Mus musculus tubby like protein 1 (Tulp1), mRNA. | | -80.80 | | 21.4 | | PHOTOTRANSDUCTION | |
| Gm15706 | LOC100045738 | | 6 | | PREDICTED: Mus musculus hypothetical protein LOC100045738; predicted gene 15706. | | -80.74 | | 32.5 | | UNKNOWN FUNCTION | |
| Ssu72 | SSU72 | | 4 | | Mus musculus Ssu72 RNA polymerase II CTD phosphatase homolog (yeast) (Ssu72), mRNA. | | -80.62 | | 39.2 | | TRANSCRIPTION/TRANSLATION | |
| Wisp1 | WISP1 | | 15 | | Mus musculus WNT1 inducible signaling pathway protein 1 (Wisp1), mRNA. | | -80.45 | | 4.2 | | CELL-CELL COMMUNICATION | |
| Esrrb | ESRRB | | 12 | | Mus musculus estrogen related receptor, beta (Esrrb), mRNA. | | -79.72 | | 5.6 | | TRANSCRIPTION/TRANSLATION | |
| Kirrel | 6720469N11RIK | | 3 | | Mus musculus kin of IRRE like (Drosophila) | | -79.15 | | 31.7 | | METABOLISM, MITO FXN | |
| Cnksr1 | CNKSR1 | | 4 | | Mus musculus connector enhancer of kinase suppressor of Ras 1 (Cnksr1), mRNA. | | -79.08 | | 23.4 | | INTRACELLULAR SIGNAL TRANSDUCTION | |
| Crx | CRX | | 7 | | Mus musculus cone-rod homeobox containing gene (Crx), mRNA. | | -78.67 | | 30.8 | | PHOTOTRANSDUCTION | |
| Tmem108 | TMEM108 | | 9 | | Mus musculus transmembrane protein 108 (Tmem108), mRNA. | | -78.28 | | 35.3 | | UNKNOWN FUNCTION | |
| Dgke | DGKE | | 11 | | Mus musculus diacylglycerol kinase, epsilon (Dgke), mRNA. | | -78.04 | | 26.8 | | METABOLISM, MITO FXN | |
| A930018I09Rik | A930018I09RIK | |  | |  | | -77.99 | | 9.6 | | UNKNOWN GENE | |
| Pde6a | PDE6A | | 18 | | Mus musculus phosphodiesterase 6A, cGMP-specific, rod, alpha (Pde6a), mRNA. | | -77.57 | | 6.1 | | PHOTOTRANSDUCTION | |
| Cacna1f | CACNA1F | | X | | Mus musculus calcium channel, voltage-dependent, alpha 1F subunit. | | -77.18 | | 20.0 | | PHOTORECEPTOR GENE | |
| Sag | ILMN_203052 | | 1 | | Mus musculus retinal S-antigen (Arr1) | | -77.04 | | 31.8 | | PHOTOTRANSDUCTION | |
| Abca4 | ABCA4 | | 3 | | Mus musculus ATP-binding cassette, sub-family A (ABC1), member 4 | | -76.36 | | 12.8 | | PHOTOTRANSDUCTION | |
| Prkab1 | PRKAB1 | | 5 | | Mus musculus protein kinase, AMP-activated, beta 1 non-catalytic subunit (Prkab1), mRNA. | | -75.85 | | 40.1 | | METABOLISM, MITO FXN | |
| Sdc4 | SDC4 | | 2 | | Mus musculus syndecan 4 (Sdc4), mRNA. | | -74.30 | | 38.1 | | CELL-CELL COMMUNICATION | |
| Whrn | WHRN | | 4 | | Mus musculus whirlin (Whrn), transcript variant 6, mRNA. | | -73.13 | | 38.5 | | RETINAL DISEASE GENE | |
| Rgs9bp | RGS9BP | | 7 | | Mus musculus regulator of G-protein signalling 9 binding protein (Rgs9bp), mRNA. | | -72.75 | | 22.1 | | PHOTOTRANSDUCTION | |
| Arl6 | ARL6 | | 16 | | Mus musculus ADP-ribosylation factor-like 6 (Arl6), mRNA. | | -72.67 | | 41.4 | | PHOTORECEPTOR GENE | |
| LOC381739 | LOC381739 | |  | |  | | -72.60 | | 39.7 | | OTHER | |
| Ccdc64 | CCDC64 | | 5 | | Mus musculus coiled-coil domain containing 64 (Ccdc64), mRNA. | | -71.91 | | 33.8 | | NEURON FUNCTION | |
| Gnaz | GNAZ | | 10 | | Mus musculus guanine nucleotide binding protein, alpha z subunit (Gnaz), mRNA. | | -71.91 | | 41.4 | | INTRACELLULAR SIGNAL TRANSDUCTION | |
| Rorb | RORB | | 19 | | Mus musculus RAR-related orphan receptor beta (Rorb), transcript variant 1, mRNA. | | -71.81 | | 25.2 | | TRANSCRIPTION/TRANSLATION | |
| AI118078 | AI118078 | | 9 | | Mus musculus expressed sequence AI118078 (AI118078, C15orf27), mRNA. | | -70.93 | | 37.5 | | UNKNOWN FUNCTION | |
| Ckmt1 | CKMT1 | | 2 | | Mus musculus creatine kinase, mitochondrial 1, ubiquitous | | -70.06 | | 40.1 | | METABOLISM, MITO FXN | |
| Cpm | CPM | | 10 | | PREDICTED: Mus musculus carboxypeptidase M (Cpm), mRNA. | | -69.61 | | 24.2 | | METABOLISM, MITO FXN | |
| 6430571L13Rik | 6430571L13RIK | | 9 | | Mus musculus RIKEN cDNA 6430571L13 gene (6430571L13Rik), mRNA. | | -69.17 | | 39.8 | | UNKNOWN FUNCTION | |
| Wdr78 | WDR78 | | 4 | | Mus musculus WD repeat domain 78 (Wdr78), mRNA. | | -68.43 | | 22.4 | | CILIA COMPONENT/FUNCTION | |
| Elovl4 | ELOVL4 | | 9 | | Mus musculus elongation of very long chain fatty acids (FEN1/Elo2, SUR4/Elo3, yeast)-like 4 (Elovl4), mRNA. | | -68.30 | | 42.9 | | PHOTOTRANSDUCTION | |
| Trafd1 | TRAFD1 | | 5 | | Mus musculus TRAF type zinc finger domain containing 1 (Trafd1), mRNA. | | -67.80 | | 42.4 | | CELL-CELL COMMUNICATION | |
| Plekhg3 | PLEKHG3 | | 12 | | Mus musculus pleckstrin homology domain containing, family G (with RhoGef domain) member 3 (Plekhg3), mRNA. | | -67.56 | | 29.9 | | METABOLISM, MITO FXN | |
| Ap2a2 | AP2A2 | | 7 | | Mus musculus adaptor protein complex AP-2, alpha 2 subunit (Ap2a2), mRNA. | | -67.14 | | 43.1 | | NEURON FUNCTION | |
| Pip4k2c | PIP4K2C | | 10 | | Mus musculus phosphatidylinositol-5-phosphate 4-kinase, type II, gamma (Pip4k2c), mRNA. | | -67.11 | | 42.9 | | METABOLISM, MITO FXN | |
| A930003A15Rik | A930003A15RIK | | 16 | | PREDICTED: Mus musculus RIKEN cDNA A930003A15 gene (A930003A15Rik), misc RNA. | | -66.36 | | 17.5 | | UNKNOWN FUNCTION | |
| ENSMUSG00000052143 | ENSMUSG00000052143 | | 9 | | PREDICTED: Mus musculus predicted gene, ENSMUSG00000052143 (ENSMUSG00000052143), mRNA. | | -66.26 | | 28.4 | | UNKNOWN FUNCTION | |
| Gas6 | GAS6 | | 8 | | Mus musculus growth arrest specific 6 (Gas6), mRNA. | | -65.63 | | 43.2 | | TRANSCRIPTION/TRANSLATION | |
| VOPP1 | AW146242 | | 6 | | Mus musculus Vesicular, overexpressed in cancer, prosurvival protein 1 (VOPP1); expressed sequence AW146242 (AW146242), mRNA. | | -65.44 | | 36.2 | | TRANSCRIPTION/TRANSLATION | |
| Pitpnm3 | PITPNM3 | | 11 | | Mus musculus PITPNM family member 3 (Pitpnm3), transcript variant 1, mRNA. | | -65.44 | | 13.5 | | PHOTORECEPTOR GENE | |
| Gadd45a | GADD45A | | 6 | | Mus musculus growth arrest and DNA-damage-inducible 45 alpha (Gadd45a), mRNA. | | -65.33 | | 33.3 | | DNA DAMAGE/REPAIR | |
| Gnb5 | GNB5 | | 9 | | Mus musculus guanine nucleotide binding protein (G protein), beta 5 (Gnb5), transcript variant 1, mRNA. | | -64.89 | | 34.2 | | INTRACELLULAR SIGNAL TRANSDUCTION | |
| 1810009A15Rik | 1810009A15RIK | | 19 | | Mus musculus RIKEN cDNA 1810009A15 gene (1810009A15Rik), mRNA. | | -63.37 | | 25.9 | | UNKNOWN FUNCTION | |
| Sema3f | SEMA3F | | 9 | | Mus musculus sema domain, immunoglobulin domain (Ig), short basic domain, secreted, (semaphorin) 3F (Sema3f), mRNA. | | -63.03 | | 27.5 | | NEURON FUNCTION | |
| Rapgef5 | RAPGEF5 | | 12 | | Mus musculus Rap guanine nucleotide exchange factor (GEF) 5 (Rapgef5), mRNA. | | -62.80 | | 24.9 | | METABOLISM, MITO FXN | |
| Ptp4a3 | PTP4A3 | | 15 | | Mus musculus protein tyrosine phosphatase 4a3 (Ptp4a3), mRNA. | | -62.65 | | 26.2 | | METABOLISM, MITO FXN | |
| 8430403J19Rik | 8430403J19RIK | |  | |  | | -62.46 | | 17.7 | | UNKNOWN GENE | |
| Lbh | LBH | | 17 | | Mus musculus limb-bud and heart (Lbh), mRNA. | | -62.39 | | 48.8 | | TRANSCRIPTION/TRANSLATION | |
| Nrl | NRL | | 14 | | Mus musculus neural retina leucine zipper gene (Nrl), mRNA. | | -62.04 | | 23.4 | | PHOTORECEPTOR GENE | |
| 3830612M24 | 3830612M24 | | 7 | | Mus musculus uncharacterized protein 3830612M24 | | -61.95 | | 37.3 | | UNKNOWN FUNCTION | |
| Rcvrn | RCVRN | | 11 | | Mus musculus recoverin (Rcvrn), mRNA. | | -61.41 | | 18.0 | | PHOTOTRANSDUCTION | |
| Sntg2 | SNTG2 | | 12 | | Mus musculus syntrophin, gamma 2 (Sntg2), mRNA. | | -61.16 | | 8.7 | | CELL ADHESION | |
| Doc2b | DOC2B | | 11 | | Mus musculus double C2, beta (Doc2b), mRNA. | | -60.53 | | 27.9 | | INTRACELLULAR TRANSPORT | |
| Eps8 | EPS8 | | 6 | | Mus musculus epidermal growth factor receptor pathway substrate 8 (Eps8), mRNA. | | -60.44 | | 35.7 | | RETINAL DISEASE GENE | |
| Ier3 | IER3 | | 17 | | Mus musculus immediate early response 3 (Ier3), mRNA. | | -60.44 | | 25.0 | | METABOLISM, MITO FXN | |
| Rnf207 | RNF207 | | 4 | | Mus musculus ring finger protein 207 (Rnf207), mRNA. | | -60.38 | | 11.9 | | UNKNOWN FUNCTION | |
| Tmem229b | 6330442E10RIK | | 12 | | Mus musculus RIKEN cDNA 6330442E10 gene; transmembrane protein 229B | | -60.34 | | 30.7 | |  | |
| Plekha2 | PLEKHA2 | | 8 | | Mus musculus pleckstrin homology domain-containing, family A (phosphoinositide binding specific) member 2 (Plekha2), mRNA. | | -59.65 | | 44.4 | | CELL ADHESION | |
| Cldn23 | CLDN23 | | 8 | | Mus musculus claudin 23 (Cldn23), mRNA. | | -59.53 | | -5.2 | | CELL-CELL COMMUNICATION | |
| Soga2 | 1110012J17RIK | | 17 | | Mus musculus SOGA family member 2; RIKEN cDNA 1110012J17 gene (1110012J17Rik), mRNA. | | -59.17 | | 45.4 | | UNKNOWN FUNCTION | |
| Ppargc1a | PPARGC1A | | 5 | | Mus musculus peroxisome proliferative activated receptor, gamma, coactivator 1 alpha (Ppargc1a), mRNA. | | -59.01 | | 32.2 | | METABOLISM, MITO FXN | |
| Arhgap24 | ARHGAP24 | | 5 | | Mus musculus Rho GTPase activating protein 24 (Arhgap24), transcript variant 1, mRNA. | | -58.31 | | 28.3 | | CELL-CELL COMMUNICATION | |
| Wdr31 | WDR31 | | 4 | | Mus musculus WD repeat domain 31 (Wdr31), mRNA. | | -57.57 | | 27.0 | | CILIA COMPONENT/FUNCTION | |
| Rpgrip1 | RPGRIP1 | | 14 | | Mus musculus retinitis pigmentosa GTPase regulator interactin protein 1 | | -57.56 | | 5.9 | | PHOTOTRANSDUCTION | |
| Prph2 | PRPH2 | | 17 | | Mus musculus peripherin 2 | | -57.45 | | 19.1 | | PHOTORECEPTOR GENE | |
| Optn | OPTN | | 2 | | Mus musculus optineurin (Optn), mRNA. | | -56.66 | | 41.3 | | NEURON FUNCTION | |
| Elfn1 | ELFN1 | | 5 | | Mus musculus leucine rich repeat and fibronectin type III, extracellular 1 (Elfn1), mRNA. | | -56.34 | | 29.5 | | METABOLISM, MITO FXN | |
| Dlg4 | DLG4 | | 11 | | Mus musculus discs, large homolog 4 (Drosophila) (Dlg4), mRNA. | | -55.05 | | 45.0 | | SYNAPTIC/SECRETORY FUNCTION | |
| Rims2 | RIMS2 | | 15 | | Mus musculus regulating synaptic membrane exocytosis 2 (Rims2), mRNA. | | -54.91 | | 45.6 | | SYNAPTIC/SECRETORY FUNCTION | |
| Rrp1b | RRP1B | | 17 | | Mus musculus ribosomal RNA processing 1 homolog B (S. cerevisiae) (Rrp1b), mRNA. | | -54.87 | | 31.7 | | TRANSCRIPTION/TRANSLATION | |
| Gas7 | GAS7 | | 11 | | Mus musculus growth arrest specific 7 (Gas7), mRNA. | | -54.80 | | 13.4 | | NEURON FUNCTION | |
| Myo7a | MYO7A | | 7 | | Mus musculus myosin VIIa (Myo7a), mRNA. | | -54.10 | | 43.6 | | INTRACELLULAR TRANSPORT | |
| Plekhf2 | PLEKHF2 | | 4 | | Mus musculus pleckstrin homology domain containing, family F (with FYVE domain) member 2 (Plekhf2), mRNA. | | -54.07 | | 25.8 | | METABOLISM, MITO FXN | |
| Ldha | LDHA | | 7 | | Mus musculus lactate dehydrogenase A (Ldha), mRNA. | | -53.42 | | 48.3 | | METABOLISM, MITO FXN | |
| Impg2 | IMPG2 | | 16 | | Mus musculus interphotoreceptor matrix proteoglycan 2 (Impg2), mRNA. | | -53.39 | | 21.4 | | PHOTORECEPTOR GENE | |
| Rd3 | 3322402L07RIK | | 1 | | Mus musculus retinal degeneration 3 (Rd3), mRNA. | | -53.23 | | 19.6 | | PHOTORECEPTOR GENE | |
| Arhgef2 | LBCL1 | | 3 | | Mus musculus rho/rac guanine nucleotide exchange factor (GEF) 2; lymphoid blast crisis-like 1 | | -53.14 | | 37.0 | | CELL-CELL COMMUNICATION | |
| 2810037O22Rik | 2810037O22RIK | | 14 | | Mus musculus RIKEN cDNA 2810037O22 gene | | -52.97 | | 44.3 | | UNKNOWN FUNCTION | |
| Tnfaip3 | TNFAIP3 | | 10 | | Mus musculus tumor necrosis factor, alpha-induced protein 3 (Tnfaip3), mRNA. | | -52.90 | | 11.7 | | METABOLISM, MITO FXN | |
| Csda | CSDA | | 6 | | Mus musculus cold shock domain protein A (Csda), mRNA. | | -52.66 | | 30.7 | | TRANSCRIPTION/TRANSLATION | |
| Supt16h | SUPT16H | | 14 | | Mus musculus suppressor of Ty 16 homolog (S. cerevisiae) (Supt16h), mRNA. | | -52.49 | | 21.1 | | TRANSCRIPTION/TRANSLATION | |
| Grtp1 | GRTP1 | | 8 | | Mus musculus GH regulated TBC protein 1 (Grtp1), mRNA. | | -52.47 | | 9.9 | | METABOLISM, MITO FXN | |
| Rbm20 | 1110018J23RIK | | 19 | | Mus musculus RNA binding motif protein 20. | | -52.31 | | 46.6 | | TRANSCRIPTION/TRANSLATION | |
| LOC100047808 | LOC100047808 | |  | | PREDICTED: Mus musculus hypothetical protein LOC100047808 (LOC100047808), mRNA. | | -51.54 | | 35.4 | | OTHER | |
| Man2a2 | MAN2A2 | | 7 | | Mus musculus mannosidase 2, alpha 2 (Man2a2), mRNA. | | -51.46 | | 32.5 | | METABOLISM, MITO FXN | |
| Nphp4 | NPHP4 | | 4 | | Mus musculus nephronophthisis 4 (juvenile) homolog (human) (Nphp4), mRNA. | | -51.19 | | 46.0 | | PHOTORECEPTOR GENE | |
| Tdrd7 | TDRD7 | | 4 | | Mus musculus tudor domain containing 7 (Tdrd7), mRNA. | | -51.11 | | 31.6 | | TRANSCRIPTION/TRANSLATION | |
| Llgl2 | LLGL2 | | 11 | | Mus musculus lethal giant larvae homolog 2 (Drosophila) (Llgl2), mRNA. | | -51.07 | | 41.6 | | METABOLISM, MITO FXN | |
| Ipmk | IPMK | | 10 | | Mus musculus inositol polyphosphate multikinase (Ipmk), mRNA. | | -51.03 | | 36.9 | | METABOLISM, MITO FXN | |
| Cngb1 | CNGB1 | | 8 | | PREDICTED: Mus musculus cyclic nucleotide gated channel beta 1 (Cngb1), mRNA. | | -50.69 | | 18.2 | | PHOTOTRANSDUCTION | |
| Fos | FOS | | 12 | | Mus musculus FBJ osteosarcoma oncogene (Fos), mRNA. | | -50.03 | | 35.7 | | TRANSCRIPTION/TRANSLATION | |
| 1190005I06Rik | 1190005I06RIK | | 8 | | Mus musculus RIKEN cDNA 1190005I06 gene (1190005I06Rik), mRNA. | | -49.66 | | 45.7 | | UNKNOWN FUNCTION | |
| Rasl11a | RASL11A | | 5 | | Mus musculus RAS-like, family 11, member A (Rasl11a), mRNA. | | -49.07 | | 29.2 | | METABOLISM, MITO FXN | |
| Nedd4l | NEDD4L | | 18 | | Mus musculus neural precursor cell expressed, developmentally down-regulated gene 4-like (Nedd4l), mRNA. | | -49.00 | | 43.9 | | METABOLISM, MITO FXN | |
| Fryl | 2310004H21RIK | | 5 | | Mus musculus furry homolog-like (Drosophila) | | -48.59 | | 31.7 | | TRANSCRIPTION/TRANSLATION | |
| Cst3 | CST3 | | 2 | | Mus musculus cystatin C (Cst3), mRNA. | | -47.76 | | 50.5 | | METABOLISM, MITO FXN | |
| Lrrc2 | LRRC2 | | 9 | | Mus musculus leucine rich repeat containing 2 (Lrrc2), mRNA. | | -47.20 | | 19.1 | | UNKNOWN FUNCTION | |
| Reep6 | REEP6 | | 10 | | Mus musculus receptor accessory protein 6 (Reep6), mRNA. | | -47.13 | | 11.5 | | UNKNOWN FUNCTION | |
| Atp1b2 | ATP1B2 | | 11 | | Mus musculus ATPase, Na+/K+ transporting, beta 2 polypeptide (Atp1b2), mRNA. | | -47.00 | | 50.7 | | SYNAPTIC/SECRETORY FUNCTION | |
| Vasn | VASN | | 16 | | Mus musculus vasorin (Vasn), mRNA. | | -46.94 | | 47.4 | | UNKNOWN FUNCTION | |
| Ddhd1 | DDHD1 | | 14 | | Mus musculus DDHD domain containing 1 | | -46.59 | | 34.2 | | METABOLISM, MITO FXN | |
| Hook3 | 5830454D03RIK | | 8 | | Mus musculus hook homolog 3 (Drosophila) | | -46.40 | | 42.2 | | INTRACELLULAR TRANSPORT | |
| Plagl2 | PLAGL2 | | 2 | | Mus musculus pleiomorphic adenoma gene-like 2 (Plagl2), mRNA. | | -46.32 | | 46.6 | | TRANSCRIPTION/TRANSLATION | |
| Otud7b | 4930463P07RIK | | 3 | | Mus musculus OTU domain containing 7B. | | -46.26 | | 47.3 | | CILIA COMPONENT/FUNCTION | |
| Cited2 | CITED2 | | 10 | | Mus musculus Cbp/p300-interacting transactivator, with Glu/Asp-rich carboxy-terminal domain, 2 (Cited2), mRNA. | | -46.08 | | 51.0 | | TRANSCRIPTION/TRANSLATION | |
| Pfkfb2 | PFKFB2 | | 1 | | Mus musculus 6-phosphofructo-2-kinase/fructose-2,6-biphosphatase 2 (Pfkfb2), mRNA. | | -46.02 | | 23.6 | | METABOLISM, MITO FXN | |
| Acadvl | ACADVL | | 11 | | Mus musculus acyl-Coenzyme A dehydrogenase, very long chain (Acadvl), mRNA. | | -45.07 | | 29.3 | | METABOLISM, MITO FXN | |
| Htra1 | HTRA1 | | 7 | | Mus musculus HtrA serine peptidase 1 (Htra1), mRNA. | | -44.95 | | 48.9 | | EXTRACELLULAR MATRIX | |
| Plxna2 | PLXNA2 | | 1 | | Mus musculus plexin A2 (Plxna2), mRNA. | | -44.34 | | 31.1 | | NEURON FUNCTION | |
| Dusp5 | LOC240672 | | 19 | | Mus musculus dual specificity phosphatase 5 | | -44.27 | | 45.1 | | METABOLISM, MITO FXN | |
| Dpysl3 | DPYSL3 | | 18 | | Mus musculus dihydropyrimidinase-like 3 (Dpysl3), mRNA. | | -43.82 | | 51.5 | | NEURON FUNCTION | |
| D430042O09Rik | D430042O09RIK | | 7 | | Mus musculus RIKEN cDNA D430042O09 gene (D430042O09Rik), mRNA. | | -43.73 | | 26.6 | | UNKNOWN FUNCTION | |
| 5330431K02Rik | 5330431K02RIK | | 13 | | Mus musculus RIKEN cDNA 5330431K02 gene | | -43.70 | | 35.0 | | UNKNOWN FUNCTION | |
| Abhd14a | DORZ1 | | 9 | | Mus musculus abhydrolase domain containing 14A | | -43.67 | | 38.2 | | UNKNOWN FUNCTION | |
| Icmt | ICMT | | 4 | | Mus musculus isoprenylcysteine carboxyl methyltransferase (Icmt), mRNA. | | -43.58 | | 29.3 | | TRANSCRIPTION/TRANSLATION | |
| Gas7 | B230343A10RIK | | 11 | | Mus musculus growth arrest specific 7 | | -42.92 | | 47.9 | | NEURON FUNCTION | |
| Adipor1 | ADIPOR1 | | 1 | | Mus musculus adiponectin receptor 1 (Adipor1), mRNA. | | -42.28 | | 52.6 | | INTRACELLULAR SIGNALING | |
| EG623230 | EG623230 | | 4 | | PREDICTED: Mus musculus predicted gene, EG623230 (EG623230), mRNA. | | -42.28 | | 52.9 | | UNKNOWN FUNCTION | |
| Arid3b | ARID3B | | 9 | | Mus musculus AT rich interactive domain 3B (BRIGHT-like) (Arid3b), mRNA. | | -41.44 | | 46.6 | | TRANSCRIPTION/TRANSLATION | |
| Heatr5a | HEATR5A | | 12 | | Mus musculus HEAT repeat containing 5A (Heatr5a), mRNA. | | -41.43 | | 24.1 | | UNKNOWN FUNCTION | |
| Impg1 | IMPG1 | | 9 | | Mus musculus interphotoreceptor matrix proteoglycan 1 (Impg1), mRNA. | | -41.40 | | 14.2 | | PHOTORECEPTOR GENE | |
| March 1 | 2900024D24Rik | | 8 | | Mus musculus membrane-associated ring finger (C3HC4) 1 (March1), mRNA. | | -41.39 | | 20.4 | | METABOLISM, MITO FXN | |
| Acsl1 | ACSL1 | | 8 | | Mus musculus acyl-CoA synthetase long-chain family member 1 (Acsl1), mRNA. | | -41.23 | | 50.2 | | METABOLISM, MITO FXN | |
| Pias3 | PIAS3 | | 3 | | Mus musculus protein inhibitor of activated STAT 3 (Pias3), transcript variant 1, mRNA. | | -41.17 | | 43.1 | | TRANSCRIPTION/TRANSLATION | |
| Xrcc6 | XRCC6 | | 15 | | Mus musculus X-ray repair complementing defective repair in Chinese hamster cells 6 (Xrcc6), mRNA. | | -41.09 | | 38.8 | | DNA DAMAGE/REPAIR | |
| LOC100047651 | LOC100047651 | |  | | PREDICTED: Mus musculus similar to FOG (LOC100047651), mRNA. | | -40.82 | | 46.0 | | OTHER | |
| Ppp3cc | PPP3CC | | 14 | | Mus musculus protein phosphatase 3, catalytic subunit, gamma isoform | | -40.60 | | 38.2 | | METABOLISM, MITO FXN | |
| Abcg4 | ABCG4 | | 9 | | Mus musculus ATP-binding cassette, sub-family G (WHITE), member 4 (Abcg4), mRNA. XM_989296 XM_989324 | | -40.57 | | 52.6 | | METABOLISM, MITO FXN | |
| Rd3 | RD3 | | 1 | | Mus musculus retinal degeneration 3 (Rd3), mRNA. | | -40.24 | | 23.0 | | PHOTORECEPTOR GENE | |
| E230024B12Rik | E230024B12RIK | |  | |  | | -40.08 | | 46.2 | | UNKNOWN GENE | |
| Fam161a | 4930430E16RIK | | 11 | | Mus musculus RIKEN cDNA 4930430E16 gene; family with sequence similarity 161, member A | | -39.92 | | 11.6 | | PHOTORECEPTOR GENE | |
| Vps37c | VPS37C | | 19 | | Mus musculus vacuolar protein sorting 37C (yeast) (Vps37c), mRNA. | | -39.58 | | 42.3 | | INTRACELLULAR TRANSPORT | |
| Pcdh21 | PCDH21 | | 14 | | Mus musculus protocadherin 21 (Pcdh21), mRNA. | | -39.03 | | 30.9 | | PHOTORECEPTOR GENE | |
| Plekhb1 | PLEKHB1 | | 7 | | Mus musculus pleckstrin homology domain containing, family B (evectins) member 1 (Plekhb1), mRNA. | | -38.54 | | 46.1 | | CILIA COMPONENT/FUNCTION | |
| LOC100044376 | LOC100044376 | | 10 | | PREDICTED: Mus musculus similar to Dual-specificity tyrosine-(Y)-phosphorylation regulated kinase 2 (LOC100044376), misc RNA. | | -38.36 | | 37.5 | | OTHER | |
| Samd7 | SAMD7 | | 3 | | Mus musculus sterile alpha motif domain containing 7 (Samd7), mRNA. | | -38.25 | | 18.9 | | UNKNOWN FUNCTION | |
| Cnga1 | CNGA1 | | 5 | | Mus musculus cyclic nucleotide gated channel alpha 1 (Cnga1), mRNA. | | -38.19 | | 13.9 | | PHOTOTRANSDUCTION | |
| Tube1 | TUBE1 | | 10 | | Mus musculus epsilon tubulin 1 | | -37.78 | | 29.8 | | METABOLISM, MITO FXN | |
| Cnbd2 | 4921517L17RIK | | 2 | | Mus musculus cyclic nucleotide binding domain containing 2 | | -37.50 | | 34.4 | | UNKNOWN FUNCTION | |
| Cd276 | CD276 | | 9 | | Mus musculus CD276 antigen (Cd276), mRNA. | | -37.27 | | 47.9 | | CELL-CELL COMMUNICATION | |
| Dusp7 | DUSP7 | | 9 | | Mus musculus dual specificity phosphatase 7 (Dusp7), mRNA. | | -37.27 | | 53.9 | | METABOLISM, MITO FXN | |
| Impdh1 | IMPDH1 | | 6 | | Mus musculus inosine 5'-phosphate dehydrogenase 1 (Impdh1), mRNA. | | -37.10 | | 26.0 | | PHOTORECEPTOR GENE | |
| Crocc | CROCC | | 4 | | Mus musculus ciliary rootlet coiled-coil, rootletin (Crocc), mRNA. | | -36.68 | | 38.4 | | CYTOSKELETAL/NUCLEAR ENVELOPE | |
| Spry3 | SPRY3 | | X\|NT_165789.2 | | Mus musculus sprouty homolog 3 (Drosophila) (Spry3), mRNA. | | -36.66 | | 43.3 | | METABOLISM, MITO FXN | |
| Rhot2 | RHOT2 | | 17 | | Mus musculus ras homolog gene family, member T2 (Rhot2), nuclear gene encoding mitochondrial protein, mRNA. | | -36.50 | | 47.9 | | METABOLISM, MITO FXN | |
| D4Bwg1540e | D4BWG1540E | | 4 | | Mus musculus DNA segment, Chr 4, Brigham & Women's Genetics 1540 expressed | | -36.47 | | 30.8 | | INTRACELLULAR SIGNALING | |
| E530004K11Rik | E530004K11RIK | |  | |  | | -36.47 | | 23.3 | | UNKNOWN GENE | |
| Gucd1 | 1110038D17RIK | | 10 | | Mus musculus guanylyl cyclase domain containing 1 | | -35.84 | | 41.2 | | UNKNOWN FUNCTION | |
| Lcn2 | LCN2 | | 2 | | Mus musculus lipocalin 2 (Lcn2), mRNA. | | -35.81 | | 29.5 | | SYNAPTIC/SECRETORY FUNCTION | |
| LOC100048116 | LOC100048116 | |  | | PREDICTED: Mus musculus similar to Jmy-pending protein (LOC100048116), mRNA. | | -35.81 | | 31.3 | | OTHER | |
| Mreg | MREG | | 1 | | Mus musculus melanoregulin (Mreg), mRNA. | | -35.81 | | 48.2 | | PHOTORECEPTOR GENE | |
| Trp53inp2 | TRP53INP2 | | 2 | | Mus musculus transformation related protein 53 inducible nuclear protein 2 (Trp53inp2), mRNA. | | -35.51 | | 54.3 | | NEURON FUNCTION | |
| Eml3 | EML3 | | 19 | | Mus musculus echinoderm microtubule associated protein like 3 (Eml3), mRNA. | | -35.43 | | 40.4 | | METABOLISM, MITO FXN | |
| Fryl | 2010313D22RIK | | 5 | | Mus musculus furry homolog-like (Drosophila) | | -35.39 | | 49.1 | | TRANSCRIPTION/TRANSLATION | |
| Slc1a7 | SLC1A7 | | 4 | | Mus musculus solute carrier family 1 (glutamate transporter), member 7 (Slc1a7), mRNA. | | -34.94 | | 38.4 | | PHOTORECEPTOR GENE | |
| Polg2 | POLG2 | | 11 | | Mus musculus polymerase (DNA directed), gamma 2, accessory subunit (Polg2), mRNA. | | -34.93 | | 25.9 | | TRANSCRIPTION/TRANSLATION | |
| Spg7 | SPG7 | | 8 | | Mus musculus spastic paraplegia 7 homolog (human) (Spg7), nuclear gene encoding mitochondrial protein, mRNA. | | -34.86 | | 45.0 | | METABOLISM, MITO FXN | |
| Stard7 | STARD7 | | 2 | | Mus musculus START domain containing 7 (Stard7), mRNA. | | -34.80 | | 42.3 | | METABOLISM, MITO FXN | |
| Gm6705 | LOC224532 | | 17 | | PREDICTED: Mus musculus predicted gene 6705 | | -34.09 | | 48.7 | | UNKNOWN FUNCTION | |
| Gnat1 | GNAT1 | | 9 | | Mus musculus guanine nucleotide binding protein, alpha transducing 1 (Gnat1), mRNA. | | -33.91 | | 15.3 | | PHOTOTRANSDUCTION | |
| Rhobtb1 | RHOBTB1 | | 10 | | Mus musculus Rho-related BTB domain containing 1 (Rhobtb1), mRNA. | | -33.41 | | 41.5 | | INTRACELLULAR SIGNAL TRANSDUCTION | |
| A930013B19Rik | A930013B19RIK | |  | |  | | -33.24 | | 3.3 | | UNKNOWN GENE | |
| Ttc8 | TTC8 | | 12 | | Mus musculus tetratricopeptide repeat domain 8 (Ttc8), transcript variant 1, mRNA. | | -33.01 | | 55.2 | | PHOTORECEPTOR GENE | |
| Kank1 | KANK1 | | 19 | | Mus musculus KN motif and ankyrin repeat domains 1 (Kank1), mRNA. | | -32.90 | | 30.0 | | CYTOSKELETAL/NUCLEAR ENVELOPE | |
| Tdrd9 | TDRD9 | | 12 | | PREDICTED: Mus musculus tudor domain containing 9 (Tdrd9), mRNA. | | -32.83 | | 38.5 | | UNKNOWN FUNCTION | |
| Rad54b | E130016E03RIK | | 4 | | Mus musculus RAD54 homolog B (S. cerevisiae) | | -32.72 | | 25.0 | | DNA damage/repair | |
| Hcn1 | C630013B14RIK | | 13 | | Mus musculus hyperpolarization-activated, cyclic nucleotide-gated K+ 1 | | -32.32 | | 40.7 | | NEURON FUNCTION | |
| Ddit4 | DDIT4 | | 10 | | Mus musculus DNA-damage-inducible transcript 4 (Ddit4), mRNA. | | -32.19 | | 56.4 | | DNA DAMAGE/REPAIR | |
| Nrm | NRM | | 17 | | Mus musculus nurim (nuclear envelope membrane protein) (Nrm), mRNA. | | -32.18 | | 40.9 | | CYTOSKELETAL/NUCLEAR ENVELOPE | |
| Timm44 | TIMM44 | | 8 | | Mus musculus translocase of inner mitochondrial membrane 44 (Timm44), mRNA. | | -32.16 | | 54.6 | | METABOLISM, MITO FXN | |
| Eepd1 | 2310005P05RIK | | 9 | | Mus musculus endonuclease/exonuclease/phosphatase family domain containing 1 | | -32.13 | | 24.0 | | DNA DAMAGE/REPAIR | |
| Exoc3l | EXOC3L | | 8 | | Mus musculus exocyst complex component 3-like (Exoc3l), mRNA. | | -31.85 | | 18.0 | | SYNAPTIC/SECRETORY FUNCTION | |
| LOC666403 | LOC666403 | | 11 | | PREDICTED: Mus musculus similar to ribosomal protein S2 (LOC666403), misc RNA. | | -31.58 | | 24.6 | | OTHER | |
| 1110018N20Rik | 1110018N20RIK | | 2 | |  | | -31.57 | | 48.8 | | UNKNOWN FUNCTION | |
| LOC100046129 | LOC100046129 | |  | | PREDICTED: Mus musculus similar to Hypocretin (orexin) receptor 1 (LOC100046129), mRNA. | | -31.47 | | 42.0 | | OTHER | |
| Atxn1 | ATXN1 | | 17 | | Mus musculus ataxin 1 (Atxn1), mRNA. | | -31.05 | | 43.8 | | NEURON FUNCTION | |
| Slc24a1 | SLC24A1 | | 9 | | Mus musculus solute carrier family 24 (sodium/potassium/calcium exchanger), member 1 (Slc24a1), mRNA. | | -30.97 | | 8.3 | | PHOTORECEPTOR GENE | |
| Map4k3 | MAP4K3 | | 17 | | Mus musculus mitogen-activated protein kinase kinase kinase kinase 3 (Map4k3), mRNA. | | -30.97 | | 55.5 | | INTRACELLULAR SIGNALING | |
| Fyco1 | 2810409M01RIK | | 9 | | Mus musculus FYVE and coiled-coil domain containing 1 | | -30.82 | | 33.1 | | INTRACELLULAR TRANSPORT | |
| Epb4.1l2 | EPB4.1L2 | | 10 | | Mus musculus erythrocyte protein band 4.1-like 2 | | -30.51 | | 34.5 | | CYTOSKELETAL/NUCLEAR ENVELOPE | |
| Tob2 | TOB2 | | 15 | | Mus musculus transducer of ERBB2, 2 | | -30.37 | | 34.7 | | TRANSCRIPTION/TRANSLATION | |
| Osbp2 | OSBP2 | | 11 | | Mus musculus oxysterol binding protein 2 (Osbp2), mRNA. | | -30.35 | | 17.9 | | INTRACELLULAR TRANSPORT | |
| Ppm1n | C79127 | | 7 | | Mus musculus protein phosphatase, Mg2+/Mn2+ dependent, 1N (putative); expressed sequence C79127 (C79127), mRNA. | | -30.33 | | 25.3 | | METABOLISM, MITO FXN | |
| Pigz | PIGZ | | 16 | | Mus musculus phosphatidylinositol glycan anchor biosynthesis, class Z (Pigz), mRNA. | | -30.28 | | 51.5 | | METABOLISM, MITO FXN | |
| BC024537 | BC024537 | | X | | [copy of repeat sequence; does not appear to represent an mRNA from a protein-coding gene.] | | -30.11 | | 24.8 | | OTHER | |
| Hs3st3b1 | HS3ST3B1 | | 11 | | Mus musculus heparan sulfate (glucosamine) 3-O-sulfotransferase 3B1 (Hs3st3b1), mRNA. | | -30.01 | | 28.9 | | METABOLISM, MITO FXN | |
| Ppp1r18 | 2310014H01RIK | | 17 | | Mus musculus Ppp1r18 protein phosphatase 1, regulatory subunit 18; RIKEN cDNA 2310014H01 gene, transcript variant 3 (2310014H01Rik), mRNA. | | -29.79 | | 50.1 | | CYTOSKELETAL/NUCLEAR ENVELOPE | |
| B430216N15Rik | B430216N15RIK | |  | |  | | -29.67 | | 43.7 | | UNKNOWN GENE | |
| Hcls1 | HCLS1 | | 16 | | Mus musculus hematopoietic cell specific Lyn substrate 1 (Hcls1), mRNA. | | -29.57 | | 12.0 | | INTRACELLULAR TRANSPORT | |
| Prickle1 | PRICKLE1 | | 15 | | Mus musculus prickle like 1 (Drosophila) (Prickle1), mRNA. | | -29.56 | | 50.0 | | TRANSCRIPTION/TRANSLATION | |
| EG545758 | EG545758 | | 5 | | Mus musculus predicted gene, EG545758 (EG545758), mRNA. | | -29.34 | | 30.3 | | UNKNOWN FUNCTION | |
| Rabgef1 | RABGEF1 | | 5 | | Mus musculus RAB guanine nucleotide exchange factor (GEF) 1 (Rabgef1), mRNA. | | -29.29 | | 35.8 | | INTRACELLULAR TRANSPORT | |
| Jam2 | JAM2 | | 16 | | Mus musculus junction adhesion molecule 2 (Jam2), mRNA. | | -29.25 | | 26.5 | | CELL-CELL COMMUNICATION | |
| Fam107b | 3110001A13RIK | | 2 | | Mus musculus RIKEN cDNA 3110001A13 gene (3110001A13Rik), mRNA. | | -29.17 | | 29.1 | | UNKNOWN FUNCTION | |
| E230008N13Rik | E230008N13RIK | | 4 | | PREDICTED: Mus musculus RIKEN cDNA E230008N13 gene (E230008N13Rik), mRNA. | | -28.93 | | 28.9 | | UNKNOWN FUNCTION | |
| Slc6a6 | SLC6A6 | | 6 | | Mus musculus solute carrier family 6 (neurotransmitter transporter, taurine), member 6 | | -28.85 | | 43.8 | | TRANSMEMBRANE TRANSPORT | |
| Camk1d | CAMK1D | | 2 | | Mus musculus calcium/calmodulin-dependent protein kinase ID (Camk1d), mRNA. | | -28.85 | | 30.4 | | METABOLISM, MITO FXN | |
| Gulo | GULO | | 14 | | Mus musculus gulonolactone (L-) oxidase (Gulo), mRNA. | | -28.67 | | 50.9 | | METABOLISM, MITO FXN | |
| 9330200H04Rik | 9330200H04RIK | |  | |  | | -28.65 | | 12.1 | | UNKNOWN GENE | |
| Pank4 | PANK4 | | 4 | | Mus musculus pantothenate kinase 4 (Pank4), mRNA. | | -28.53 | | 58.6 | | METABOLISM, MITO FXN | |
| Mc1r | MC1R | | 8 | | Mus musculus melanocortin 1 receptor (Mc1r), mRNA. | | -28.47 | | 10.6 | | CELL-CELL COMMUNICATION | |
| Prom1 | PROM | | 5 | | Mus musculus prominin 1 | | -28.38 | | 19.4 | | PHOTORECEPTOR GENE | |
| Ebpl | EBPL | | 14 | | Mus musculus emopamil binding protein-like (Ebpl), mRNA. | | -28.32 | | 46.9 | | METABOLISM, MITO FXN | |
| Galnt10 | GALNT10 | | 11 | | Mus musculus UDP-N-acetyl-alpha-D-galactosamine:polypeptide N-acetylgalactosaminyltransferase 10 (Galnt10), mRNA. | | -28.25 | | 47.6 | | METABOLISM, MITO FXN | |
| Nme5 | NME5 | | 18 | | Mus musculus non-metastatic cells 5, protein expressed in (nucleoside-diphosphate kinase) (Nme5), mRNA. | | -28.11 | | 50.2 | | CILIA COMPONENT/FUNCTION | |
| LOC547380 | LOC547380 | | 4 | | PREDICTED: Mus musculus similar to castor homolog 1, zinc finger (LOC547380), misc RNA. | | -27.96 | | 24.0 | | OTHER | |
| Fance | FANCE | | 17 | | PREDICTED: Mus musculus Fanconi anemia, complementation group E (Fance), mRNA. | | -27.91 | | 56.6 | | DNA DAMAGE/REPAIR | |
| Bmp15 | BMP15 | | X | | Mus musculus bone morphogenetic protein 15 (Bmp15), mRNA. | | -27.88 | | 22.5 | | CELL-CELL COMMUNICATION | |
| Hemk1 | HEMK1 | | 9 | | Mus musculus HemK methyltransferase family member 1 (Hemk1), mRNA. | | -27.87 | | 56.1 | | TRANSCRIPTION/TRANSLATION | |
| 5430411C19Rik | 5430411C19RIK | | 17 | | PREDICTED: Mus musculus RIKEN cDNA 5430411C19 gene (5430411C19Rik), mRNA. | | -27.79 | | 34.8 | | UNKNOWN FUNCTION | |
| Pmm1 | PMM1 | | 15 | | Mus musculus phosphomannomutase 1 (Pmm1), mRNA. | | -27.78 | | 54.0 | | METABOLISM, MITO FXN | |
| Dnahc9 | DNAHC9 | | 11 | | PREDICTED: Mus musculus dynein, axonemal, heavy chain 9, transcript variant 2 (Dnahc9), mRNA. | | -27.78 | | 9.9 | | INTRACELLULAR TRANSPORT | |
| Heg1 | HEG1 | | 16 | | Mus musculus HEG homolog 1 (zebrafish) (Heg1), mRNA. | | -27.53 | | 24.3 | | CELL-CELL COMMUNICATION | |
| Tmem86a | TMEM86A | | 7 | | Mus musculus transmembrane protein 86A (Tmem86a), mRNA. | | -27.51 | | 55.6 | | UNKNOWN FUNCTION | |
| Ubtd1 | UBTD1 | | 19 | | Mus musculus ubiquitin domain containing 1 (Ubtd1), mRNA. | | -27.51 | | 26.9 | | UNKNOWN FUNCTION | |
| Col20a1 | COL20A1 | | 2 | | PREDICTED: Mus musculus collagen, type XX, alpha 1 (Col20a1), mRNA. | | -27.48 | | 25.3 | | EXTRACELLULAR MATRIX | |
| OTTMUSG00000005065 | OTTMUSG00000005065 | | 11 | | Mus musculus predicted gene, OTTMUSG00000005065 (OTTMUSG00000005065), mRNA. | | -27.48 | | 8.3 | | UNKNOWN FUNCTION | |
| Pcdh15 | PCDH15 | | 10 | | Mus musculus protocadherin 15 | | -27.42 | | 22.0 | | PHOTORECEPTOR GENE | |
| Rbp3 | RBP3 | | 14 | | Mus musculus retinol binding protein 3, interstitial (Rbp3), mRNA. | | -27.28 | | 25.3 | | PHOTOTRANSDUCTION | |
| Stx3 | STX3 | | 19 | | Mus musculus syntaxin 3 (Stx3), transcript variant C, mRNA. | | -27.28 | | 45.5 | | SYNAPTIC/SECRETORY FUNCTION | |
| Glp2r | GLP2R | | 11 | | Mus musculus glucagon-like peptide 2 receptor (Glp2r), mRNA. | | -27.04 | | 38.5 | | CELL-CELL COMMUNICATION | |
| C730029F17Rik | C730029F17RIK | |  | |  | | -27.02 | | 53.0 | | UNKNOWN GENE | |
| Klhdc8b | KLHDC8B | | 9 | | Mus musculus kelch domain containing 8B (Klhdc8b), mRNA. | | -26.77 | | 40.7 | | UNKNOWN FUNCTION | |
| Pwwp2b | PWWP2B | | 7 | | Mus musculus PWWP domain containing 2B (Pwwp2b), transcript variant 2, mRNA. | | -26.69 | | 46.2 | | UNKNOWN FUNCTION | |
| Zfp385a | ZFP385A | | 15 | | Mus musculus zinc finger protein 385A (Zfp385a), mRNA. | | -26.57 | | 40.7 | | TRANSCRIPTION/TRANSLATION | |
| Dixdc1 | 4930563F16RIK | | 9 | | Mus musculus DIX domain containing 1 | | -26.55 | | 44.1 | | NEURON FUNCTION | |
| Chst3 | CHST3 | | 10 | | Mus musculus carbohydrate (chondroitin 6/keratan) sulfotransferase 3 (Chst3), mRNA. | | -26.50 | | 32.9 | | METABOLISM, MITO FXN | |
| Tmem35 | TMEM35 | | X | | Mus musculus transmembrane protein 35 (Tmem35), mRNA. | | -26.39 | | 53.5 | | UNKNOWN FUNCTION | |
| Arsg | ARSG | | 11 | | Mus musculus arylsulfatase G (Arsg), mRNA. | | -26.39 | | 44.0 | | METABOLISM, MITO FXN | |
| Rhpn1 | RHPN1 | | 15 | | Mus musculus rhophilin, Rho GTPase binding protein 1 (Rhpn1), mRNA. | | -26.26 | | 38.5 | | METABOLISM, MITO FXN | |
| Rhot1 | RHOT1 | | 11 | | Mus musculus ras homolog gene family, member T1 (Rhot1), mRNA. | | -26.03 | | 60.4 | | INTRACELLULAR SIGNAL TRANSDUCTION | |
| Sgtb | SGTB | | 13 | | Mus musculus small glutamine-rich tetratricopeptide repeat (TPR)-containing, beta (Sgtb), mRNA. | | -25.95 | | 48.9 | | METABOLISM, MITO FXN | |
| Glo1 | GLO1 | | 17 | | Mus musculus glyoxalase 1 (Glo1), mRNA. | | -25.77 | | 60.9 | | METABOLISM, MITO FXN | |
| LOC381140 | LOC381140 | |  | |  | | -25.73 | | 56.3 | | OTHER | |
| Myo1g | MYO1G | | 11 | | Mus musculus myosin IG (Myo1g), mRNA. | | -25.42 | | 21.9 | | SYNAPTIC/SECRETORY FUNCTION | |
| Guk1 | GUK1 | | 11 | | Mus musculus guanylate kinase 1 | | -25.38 | | 54.0 | | METABOLISM, MITO FXN | |
| scl0001534.1_16 | SCL0001534.1_16 | | | |  | | -25.36 | | 45.9 | | UNKNOWN GENE | |
| Grm8 | GRM8 | | 6 | | Mus musculus glutamate receptor, metabotropic 8 (Grm8), mRNA. | | -25.31 | | 37.1 | | SYNAPTIC/SECRETORY FUNCTION | |
| Dcun1d3 | DCUN1D3 | | 7 | | Mus musculus DCN1, defective in cullin neddylation 1, domain containing 3 (S. cerevisiae) (Dcun1d3), mRNA. | | -25.12 | | 50.9 | | METABOLISM, MITO FXN | |
| Ndr3 | NDR3 | | 2 | | Mus musculus N-myc downstream regulated gene 3 | | -25.09 | | 19.9 | | NEURON FUNCTION | |
| Dlc1 | DLC1 | | 8 | | Mus musculus deleted in liver cancer 1 (Dlc1), mRNA. | | -25.08 | | 38.0 | | CYTOSKELETAL/NUCLEAR ENVELOPE | |
| Crb1 | CRB1 | | 1 | | Mus musculus crumbs homolog 1 (Drosophila) (Crb1), mRNA. | | -25.08 | | 53.2 | | CELL-CELL COMMUNICATION | |
| Fam53B | A930008G19RIK | | 7 | | Mus musculus RIKEN cDNA A930008G19 gene (A930008G19Rik), mRNA. | | -24.98 | | 26.0 | | UNKNOWN FUNCTION | |
| Pkd1 | PKD1 | | 17 | | Mus musculus polycystic kidney disease 1 homolog (Pkd1), mRNA. | | -24.91 | | 50.4 | | CILIA COMPONENT/FUNCTION | |
| Emid2 | EMID2 | | 5 | | Mus musculus EMI domain containing 2 (Emid2), mRNA. | | -24.69 | | 18.7 | | EXTRACELLULAR MATRIX | |
| Zfp654 | ZFP654 | | 16 | | Mus musculus zinc finger protein 654 (Zfp654), mRNA. | | -24.51 | | 42.8 | | TRANSCRIPTION/TRANSLATION | |
| Myo10 | MYO10 | | 15 | | Mus musculus myosin X (Myo10), mRNA. | | -24.43 | | 27.2 | | CYTOSKELETAL/NUCLEAR ENVELOPE | |
| Snrk | SNRK | | 9 | | Mus musculus SNF related kinase (Snrk), mRNA. | | -24.31 | | 50.7 | | METABOLISM, MITO FXN | |
| LOC676420 | LOC676420 | |  | | PREDICTED: Mus musculus similar to ceramide kinases (LOC676420), misc RNA. | | -24.27 | | 51.1 | | OTHER | |
| Atxn1 | 2900016G23RIK | | 13 | | Mus musculus ataxin 1 | | -24.17 | | 37.1 | | NEURON FUNCTION | |
| Plekhb2 | PLEKHB2 | | 1 | | Mus musculus pleckstrin homology domain containing, family B (evectins) member 2 (Plekhb2), mRNA. | | -23.97 | | 61.8 | | UNKNOWN FUNCTION | |
| Ext1 | EXT1 | | 15 | | Mus musculus exostoses (multiple) 1 (Ext1), mRNA. | | -23.92 | | 49.9 | | METABOLISM, MITO FXN | |
| Hspb6 | HSPB6 | | 7 | | Mus musculus heat shock protein, alpha-crystallin-related, B6 (Hspb6), mRNA. | | -23.91 | | 37.1 | | METABOLISM, MITO FXN | |
| Mier2 | 2700087H15RIK | | 10 | | mesoderm induction early response 1, family member 2 | | -23.91 | | 61.6 | | TRANSCRIPTION/TRANSLATION | |
| Usp6nl | USP6NL | | 2 | | Mus musculus USP6 N-terminal like (Usp6nl), transcript variant 1, mRNA. | | -23.88 | | 51.3 | | METABOLISM, MITO FXN | |
| Stk35 | STK35 | | 2 | | Mus musculus serine/threonine kinase 35 (Stk35), transcript variant 2, mRNA. | | -23.85 | | 27.2 | | METABOLISM, MITO FXN | |
| Zfp516 | ZFP516 | | 18 | | Mus musculus zinc finger protein 516 (Zfp516), mRNA. | | -23.57 | | 47.0 | | TRANSCRIPTION/TRANSLATION | |
| Arhgef26 | 4631416L12RIK | | 3 | | Mus musculus Rho guanine nucleotide exchange factor 26; RIKEN cDNA 4631416L12 gene (4631416L12Rik), mRNA. | | -23.53 | | 16.4 | | METABOLISM, MITO FXN | |
| Fam3c | D6WSU176E | | 6 | | Mus musculus family with sequence similarity 3, member C | | -23.44 | | 50.3 | | CELL-CELL COMMUNICATION | |
| E430025L11Rik | E430025L11RIK | |  | |  | | -23.41 | | 19.8 | | UNKNOWN GENE | |
| Crxos1 | CRXOS1 | | 7 | | Mus musculus Crx opposite strand transcript 1 (Crxos1), mRNA. | | -23.39 | | 11.2 | | TRANSCRIPTION/TRANSLATION | |
| Tmem106c | TMEM106C | | 15 | | Mus musculus transmembrane protein 106C (Tmem106c), mRNA. | | -23.20 | | 51.6 | | UNKNOWN FUNCTION | |
| Lrrc1 | LRRC1 | | 9 | | Mus musculus leucine rich repeat containing 1 (Lrrc1), mRNA. | | -23.14 | | 45.4 | | UNKNOWN FUNCTION | |
| Rab43 | 2500004H21RIK | | 6 | | Mus musculus RAB43, member RAS oncogene family | | -23.09 | | 49.7 | | INTRACELLULAR TRANSPORT | |
| C130057N11Rik | C130057N11RIK | | 2 | |  | | -23.04 | | 60.2 | | UNKNOWN FUNCTION | |
| Fbxo36 | FBXO36 | | 1 | | Mus musculus F-box protein 36 (Fbxo36), mRNA. | | -23.01 | | 52.9 | | UNKNOWN FUNCTION | |
| C030005D05Rik | C030005D05RIK | |  | |  | | -22.97 | | 37.6 | | UNKNOWN GENE | |
| Akna | AKNA | | 4 | | Mus musculus AT-hook transcription factor (Akna), mRNA. | | -22.89 | | 39.3 | | TRANSCRIPTION/TRANSLATION | |
| Camta2 | CAMTA2 | | 11 | | Mus musculus calmodulin binding transcription activator 2 (Camta2), mRNA. | | -22.80 | | 62.4 | | TRANSCRIPTION/TRANSLATION | |
| Icmt | 1700008E11RIK | | 4 | | Mus musculus isoprenylcysteine carboxyl methyltransferase (Icmt), mRNA. | | -22.71 | | 20.2 | | TRANSCRIPTION/TRANSLATION | |
| LOC100046959 | LOC100046959 | |  | | PREDICTED: Mus musculus similar to zinc finger protein 533 (LOC100046959), mRNA. | | -22.70 | | 53.3 | | OTHER | |
| Armc9 | 4930438O05RIK | | 1 | | Mus musculus armidillo repeat containing 9 | | -22.55 | | 44.3 | | UNKNOWN FUNCTION | |
| Bbs7 | BBS7 | | 3 | | Mus musculus Bardet-Biedl syndrome 7 (Bbs7), mRNA. | | -22.51 | | 39.3 | | PHOTORECEPTOR GENE | |
| Sntb2 | SNTB2 | | 8 | | Mus musculus syntrophin, basic 2 | | -22.41 | | 29.2 | | CELL ADHESION | |
| Ccdc127 | C130002N06 | | 13 | | Mus musculus doiled-doil domain containing 127 | | -22.30 | | 54.9 | | UNKNOWN FUNCTION | |
| Usp2 | USP2 | | 9 | | Mus musculus ubiquitin specific peptidase 2 (Usp2), transcript variant 2, mRNA. | | -22.29 | | 34.9 | | METABOLISM, MITO FXN | |
| A530021P12Rik | A530021P12RIK | |  | |  | | -22.05 | | 32.4 | | UNKNOWN GENE | |
| Shisa2 | SHISA2 | | 14 | | Mus musculus shisa homolog 2 (Xenopus laevis) (Shisa2), mRNA. | | -22.02 | | 61.1 | | CELL-CELL COMMUNICATION | |
| Eltd1 | ELTD1 | | 3 | | Mus musculus EGF, latrophilin seven transmembrane domain containing 1 (Eltd1), mRNA. | | -21.99 | | 59.2 | | CELL-CELL COMMUNICATION | |
| Vldlr | VLDLR | | 19 | | Mus musculus very low density lipoprotein receptor (Vldlr), mRNA. | | -21.89 | | 45.1 | | METABOLISM, MITO FXN | |
| BC034090 | BC034090 | | 1 | | PREDICTED: Mus musculus cDNA sequence BC034090, transcript variant 1 (BC034090), mRNA. | | -21.80 | | 47.5 | | UNKNOWN FUNCTION | |
| Ramp3 | RAMP3 | | 11 | | Mus musculus receptor (calcitonin) activity modifying protein 3 (Ramp3), mRNA. | | -21.80 | | 17.4 | | METABOLISM, MITO FXN | |
| Zfp533 | ZFP533 | | 2 | | Mus musculus zinc finger protein 533 (Zfp533), mRNA. | | -21.68 | | 62.8 | | UNKNOWN FUNCTION | |
| Akap6 | AKAP6 | | 12 | | Mus musculus A kinase (PRKA) anchor protein 6 (Akap6), mRNA. | | -21.65 | | 44.2 | | INTRACELLULAR SIGNAL TRANSDUCTION | |
| D130092D14Rik | D130092D14RIK | |  | |  | | -21.65 | | 32.1 | | UNKNOWN GENE | |
| Tnnt1 | TNNT1 | | 7 | | Mus musculus troponin T1, skeletal, slow (Tnnt1), mRNA. | | -21.58 | | 60.5 | | INTRACELLULAR TRANSPORT | |
| Alox5ap | ALOX5AP | | 5 | | Mus musculus arachidonate 5-lipoxygenase activating protein | | -21.32 | | 31.7 | | METABOLISM, MITO FXN | |
| Stac2 | STAC2 | | 11 | | Mus musculus SH3 and cysteine rich domain 2 (Stac2), mRNA. | | -21.22 | | 60.4 | | NEURON FUNCTION | |
| Tmod1 | TMOD1 | | 4 | | Mus musculus tropomodulin 1 (Tmod1), mRNA. | | -21.18 | | 44.7 | | CYTOSKELETAL/NUCLEAR ENVELOPE | |
| Mdm1 | MDM1 | | 10 | | Mus musculus transformed mouse 3T3 cell double minute 1 (Mdm1), transcript variant 2, mRNA. | | -21.07 | | 20.9 | | PHOTORECEPTOR GENE | |
| Stk17b | STK17B | | 1 | | Mus musculus serine/threonine kinase 17b (apoptosis-inducing) (Stk17b), mRNA. | | -21.02 | | 43.3 | | METABOLISM, MITO FXN | |
| Slc7a8 | SLC7A8 | | 14 | | Mus musculus solute carrier family 7 (cationic amino acid transporter, y+ system), member 8 (Slc7a8), mRNA. | | -20.88 | | 46.0 | | TRANSMEMBRANE TRANSPORT | |
| Mybphl | MYBPHL | | 3 | | Mus musculus myosin binding protein H-like (Mybphl), mRNA. | | -20.84 | | 21.2 | | UNKNOWN FUNCTION | |
| LOC100044204 | LOC100044204 | |  | | PREDICTED: Mus musculus hypothetical protein LOC100044204 (LOC100044204), mRNA. | | -20.80 | | 33.5 | | OTHER | |
| Gldc | GLDC | | 19 | | Mus musculus glycine decarboxylase (Gldc), mRNA. | | -20.78 | | 62.2 | | METABOLISM, MITO FXN | |
| A630056H20Rik | A630056H20RIK | |  | |  | | -20.63 | | 52.9 | | UNKNOWN GENE | |
| Slc24a1 | LOC214111 | | 9 | | Mus musculus solute carrier family 24 (sodium/potassium/calcium exchanger), member 1 | | -20.63 | | 17.1 | | PHOTORECEPTOR GENE | |
| Slc25a25 | SLC25A25 | | 2 | | Mus musculus solute carrier family 25 (mitochondrial carrier, phosphate carrier), member 25 (Slc25a25), nuclear gene encoding mitochondrial protein, mRNA. | | -20.39 | | 36.0 | | METABOLISM, MITO FXN | |
| Wdr17 | WDR17 | | 8 | | Mus musculus WD repeat domain 17 (Wdr17), mRNA. | | -20.34 | | 17.1 | | RETINAL DISEASE GENE | |
| Pex5l | PEX5L | | 3 | | Mus musculus peroxisomal biogenesis factor 5-like (Pex5l), mRNA. | | -20.32 | | 13.8 | | METABOLISM, MITO FXN | |
| Pdc | PDC | | 1 | | Mus musculus phosducin (Pdc), mRNA. | | -20.13 | | 23.2 | | PHOTOTRANSDUCTION | |
| 9630018J20Rik | 9630018J20RIK | |  | |  | | -19.95 | | 20.4 | | UNKNOWN GENE | |
| 2600011E07Rik | 2600011E07RIK | | 14 | | Mus musculus RIKEN cDNA 2600011E07 gene (2600011E07Rik), mRNA. | | -19.88 | | 57.3 | | UNKNOWN GENE | |
| Fam123a | A130030M01RIK | | 14 | | Mus musculus family with sequence similarity 123, member A | | -19.71 | | 24.0 | | INTRACELLULAR SIGNALING | |
| LOC100039590 | LOC100039590 | | 8 | | PREDICTED: Mus musculus similar to transforming growth factor, beta receptor III (betaglycan, 300kDa) (LOC100039590), mRNA. | | -19.70 | | 32.9 | | UNKNOWN GENE | |
| Hr | HR | | 14 | | Mus musculus hairless (Hr), mRNA. | | -19.70 | | 52.4 | | TRANSCRIPTION/TRANSLATION | |
| Gramd1b | A930008A22RIK | | 9 | | Mus musculus GRAM domain containing 1B | | -19.66 | | 45.5 | | UNKNOWN FUNCTION | |
| Pkm | PKM2 | | 9 | | Mus musculus pyruvate kinase, muscle | | -19.63 | | 56.4 | | METABOLISM, MITO FXN | |
| Mns1 | MNS1 | | 9 | | Mus musculus meiosis-specific nuclear structural protein 1 (Mns1), mRNA. | | -19.61 | | 27.5 | | CILIA COMPONENT/FUNCTION | |
| Myrip | MYRIP | | 9 | | Mus musculus myosin VIIA and Rab interacting protein (Myrip), mRNA. | | -19.60 | | 33.0 | | INTRACELLULAR TRANSPORT | |
| LOC382391 | LOC382391 | |  | |  | | -19.58 | | 41.2 | | OTHER | |
| Lmbr1l | LMBR1L | | 15 | | Mus musculus limb region 1 like (Lmbr1l), mRNA. | | -19.46 | | 59.4 | | METABOLISM, MITO FXN | |
| Ahcyl1 | AHCYL1 | | 3 | | Mus musculus S-adenosylhomocysteine hydrolase-like 1 (Ahcyl1), mRNA. | | -19.45 | | 52.7 | | TRANSCRIPTION/TRANSLATION | |
| Ccdc24 | LOC381546 | | 4 | | Mus musculus coiled-coil domain containing 24 | | -19.44 | | 16.4 | | UNKNOWN FUNCTION | |
| Kcnj14 | KCNJ14 | | 7 | | Mus musculus potassium inwardly-rectifying channel, subfamily J, member 14 (Kcnj14), mRNA. | | -19.34 | | 7.6 | | INTRACELLULAR TRANSPORT | |
| Ttll3 | TTLL3 | | 6 | | Mus musculus tubulin tyrosine ligase-like family, member 3 (Ttll3), mRNA. | | -19.31 | | 27.1 | | CILIA COMPONENT/FUNCTION | |
| Tspan7 | TSPAN7 | | X | | Mus musculus tetraspanin 7 (Tspan7), mRNA. | | -19.27 | | 64.8 | | CELL-CELL COMMUNICATION | |
| LOC100047090 | LOC100047090 | |  | | PREDICTED: Mus musculus similar to VIP2 receptor for vasoactive intestinal peptide (VIP) (LOC100047090), misc RNA. | | -19.20 | | 50.7 | | OTHER | |
| Tmem138 | TMEM138 | | 19 | | Mus musculus transmembrane protein 138 (Tmem138), mRNA. | | -19.07 | | 54.8 | | CILIA COMPONENT/FUNCTION | |
| Plekha6 | PLEKHA6 | | 1 | | Mus musculus pleckstrin homology domain containing, family A member 6 (Plekha6), mRNA. | | -18.99 | | 45.6 | | UNKNOWN FUNCTION | |
| Pcbp3 | PCBP3 | | 10 | | Mus musculus poly(rC) binding protein 3 (Pcbp3), mRNA. | | -18.91 | | 65.0 | | TRANSCRIPTION/TRANSLATION | |
| 4632418H02Rik | 4632418H02RIK | | 9 | | Mus musculus RIKEN cDNA 4632418H02 gene | | -18.81 | | 36.7 | | UNKNOWN FUNCTION | |
| Adrb2 | ADRB2 | | 18 | | Mus musculus adrenergic receptor, beta 2 (Adrb2), mRNA. | | -18.79 | | 18.8 | | SYNAPTIC/SECRETORY FUNCTION | |
| Lpcat2 | LPCAT2 | | 8 | | Mus musculus lysophosphatidylcholine acyltransferase 2 (Lpcat2), mRNA. | | -18.70 | | 28.9 | | METABOLISM, MITO FXN | |
| Tmem27 | TMEM27 | | X | | Mus musculus transmembrane protein 27 (Tmem27), mRNA. | | -18.69 | | 34.7 | | METABOLISM, MITO FXN | |
| Casz1 | CASZ1 | | 4 | | Mus musculus castor homolog 1, zinc finger (Drosophila) (Casz1), mRNA. | | -18.68 | | 52.7 | | TRANSCRIPTION/TRANSLATION | |
| Hnt | HNT | | 9 | | Mus musculus neurotrimin (Hnt), mRNA. | | -18.62 | | 41.0 | | NEURON FUNCTION | |
| Plk1 | PLK1 | | 7 | | Mus musculus polo-like kinase 1 (Drosophila) (Plk1), mRNA. | | -18.61 | | 7.3 | | METABOLISM, MITO FXN | |
| Armc9 | ARMC9 | | 1 | | Mus musculus armadillo repeat containing 9 (Armc9), transcript variant 1, mRNA. | | -18.56 | | 57.0 | | UNKNOWN FUNCTION | |
| Hsbp1l1 | 1810005K13RIK | | 18 | | Mus musculus heat shock factor binding protein 1-like 1 | | -18.51 | | 27.8 | | UNKNOWN FUNCTION | |
| Rgs16 | RGS16 | | 1 | | Mus musculus regulator of G-protein signaling 16 (Rgs16), mRNA. | | -18.51 | | 61.7 | | INTRACELLULAR SIGNALING | |
| Klf9 | KLF9 | | 19 | | Mus musculus Kruppel-like factor 9 (Klf9), mRNA. | | -18.30 | | 63.0 | | TRANSCRIPTION/TRANSLATION | |
| Tapt1 | TAPT1 | | 5 | | Mus musculus transmembrane anterior posterior transformation 1 (Tapt1), mRNA. | | -18.23 | | 65.3 | | CELL-CELL COMMUNICATION | |
| Peli3 | PELI3 | | 19 | | Mus musculus pellino 3 (Peli3), mRNA. | | -18.04 | | 33.1 | | METABOLISM, MITO FXN | |
| 1700003M02Rik | 1700003M02RIK | | 4 | | Mus musculus RIKEN cDNA 1700003M02 gene (1700003M02Rik), mRNA. | | -17.99 | | 40.1 | | UNKNOWN FUNCTION | |
| D16Ertd472e | D16ERTD472E | | 16 | | Mus musculus DNA segment, Chr 16, ERATO Doi 472, expressed (D16Ertd472e), mRNA. | | -17.90 | | 33.0 | | UNKNOWN FUNCTION | |
| Bbs5 | BBS5 | | 2 | | Mus musculus Bardet-Biedl syndrome 5 (human) (Bbs5), mRNA. | | -17.77 | | 41.1 | | PHOTORECEPTOR GENE | |
| Atg16l1 | ATG16L1 | | 1 | | Mus musculus autophagy-related 16-like 1 (yeast) (Atg16l1), transcript variant b, mRNA. | | -17.75 | | 64.9 | | METABOLISM, MITO FXN | |
| Zdhhc7 | ZDHHC7 | | 8 | | Mus musculus zinc finger, DHHC domain containing 7 (Zdhhc7), mRNA. | | -17.67 | | 65.0 | | METABOLISM, MITO FXN | |
| LOC239143 | LOC239143 | |  | |  | | -17.64 | | 21.8 | | UNKNOWN GENE | |
| Ubxn11 | UBXN11 | | 4 | | Mus musculus UBX domain protein 11 (Ubxn11), mRNA. | | -17.58 | | 30.6 | | METABOLISM, MITO FXN | |
| LOC386078 | LOC386078 | |  | |  | | -17.56 | | 63.7 | | OTHER | |
| Adcy6 | ADCY6 | | 15 | | Mus musculus adenylate cyclase 6 (Adcy6), mRNA. | | -17.49 | | 28.4 | | METABOLISM, MITO FXN | |
| Osgep | OSGEP | | 14 | | Mus musculus O-sialoglycoprotein endopeptidase (Osgep), mRNA. | | -17.42 | | 61.4 | | METABOLISM, MITO FXN | |
| Dhh | DHH | | 15 | | Mus musculus desert hedgehog (Dhh), mRNA. | | -17.28 | | 17.6 | | CELL-CELL COMMUNICATION | |
| LOC100046120 | LOC100046120 | |  | | PREDICTED: Mus musculus similar to clusterin (LOC100046120), mRNA. | | -17.25 | | 66.1 | | OTHER | |
| Nadkd1 | 1110020G09RIK | | 15 | | Mus musculus NAD kinase domain containing 1 | | -17.25 | | 26.9 | | METABOLISM, MITO FXN | |
| Cln3 | CLN3 | | 7 | | Mus musculus ceroid lipofuscinosis, neuronal 3, juvenile (Batten, Spielmeyer-Vogt disease) (Cln3), mRNA. | | -17.24 | | 61.6 | | METABOLISM, MITO FXN | |
| Lrrc46 | LRRC46 | | 11 | | Mus musculus leucine rich repeat containing 46 (Lrrc46), mRNA. | | -17.12 | | 49.1 | | UNKNOWN FUNCTION | |
| LOC382163 | LOC382163 | |  | |  | | -17.01 | | 52.9 | | OTHER | |
| Sdk1 | SDK1 | | 5 | | Mus musculus sidekick homolog 1 (chicken) (Sdk1), mRNA. | | -16.93 | | 59.6 | | CELL ADHESION | |
| Syt7 | B230112P13RIK | | 19 | | Mus musculus synaptotagmin VII | | -16.93 | | 64.3 | | SYNAPTIC/SECRETORY FUNCTION | |
| Chst1 | CHST1 | | 2 | | Mus musculus carbohydrate (keratan sulfate Gal-6) sulfotransferase 1 (Chst1), mRNA. | | -16.83 | | 65.3 | | METABOLISM, MITO FXN | |
| LOC229395 | LOC229395 | |  | |  | | -16.82 | | -5.6 | | OTHER | |
| LOC100048299 | LOC100048299 | |  | | PREDICTED: Mus musculus similar to Myn protein - mouse (LOC100048299), mRNA. | | -16.81 | | 64.0 | | OTHER | |
| Bsg | BSG | | 10 | | Mus musculus basigin (Bsg), transcript variant 1, mRNA. | | -16.80 | | 64.5 | | CELL-CELL COMMUNICATION | |
| Tgm4 | TGM4 | | 9 | | Mus musculus transglutaminase 4 (prostate) (Tgm4), mRNA. | | -16.79 | | 27.7 | | METABOLISM, MITO FXN | |
| LOC100044089 | LOC100044089 | | 3 | | PREDICTED: Mus musculus similar to potassium channel regulatory protein KChAP (LOC100044089), mRNA. | | -16.55 | | 46.4 | | OTHER | |
| Slc25a18 | SLC25A18 | | 6 | | Mus musculus solute carrier family 25 (mitochondrial carrier), member 18 (Slc25a18), mRNA. | | -16.55 | | 42.8 | | METABOLISM, MITO FXN | |
| Mfap3l | 4933428A15RIK | | 8 | | Mus musculus microfibrillar-associated protein 3-like | | -16.33 | | 65.4 | | UNKNOWN FUNCTION | |
| Ptpn21 | PTPN21 | | 12 | | Mus musculus protein tyrosine phosphatase, non-receptor type 21 | | -16.30 | | 49.8 | | METABOLISM, MITO FXN | |
| Dynlrb2 | DYNLRB2 | | 8 | | Mus musculus dynein light chain roadblock-type 2 (Dynlrb2), mRNA. | | -16.20 | | 60.5 | | INTRACELLULAR TRANSPORT | |
| Mical2 | 9530064J02 | | 7 | | Mus musculus microtubule associated monoxygenase, calponin and LIM domain containing 2 | | -16.19 | | 57.1 | | METABOLISM, MITO FXN | |
| Pcdhga4 | PCDHGA4 | | 18 | | Mus musculus protocadherin gamma subfamily A, 4 (Pcdhga4), mRNA. | | -16.16 | | 41.1 | | NEURON FUNCTION | |
| Mt1 | MT1 | | 8 | | Mus musculus metallothionein 1 (Mt1), mRNA. | | -16.07 | | 66.0 | | METABOLISM, MITO FXN | |
| Lnx2 | LNX2 | | 5 | | Mus musculus ligand of numb-protein X 2 (Lnx2), mRNA. | | -16.04 | | 58.8 | | METABOLISM, MITO FXN | |
| Pla2g5 | PLA2G5 | | 4 | | Mus musculus phospholipase A2, group V (Pla2g5), mRNA. | | -16.03 | | 50.1 | | METABOLISM, MITO FXN | |
| Lass4 | LASS4 | | 8 | | Mus musculus longevity assurance homolog 4 (S. cerevisiae) (Lass4), mRNA. | | -16.03 | | 42.9 | | METABOLISM, MITO FXN | |
| Synm | SYNM | | 7 | | Mus musculus synemin, intermediate filament protein (Synm), transcript variant 1, mRNA. | | -16.02 | | 43.7 | | CYTOSKELETAL/NUCLEAR ENVELOPE | |
| Pcp4l1 | PCP4L1 | | 1 | | PREDICTED: Mus musculus Purkinje cell protein 4-like 1 (Pcp4l1), mRNA. | | -15.83 | | 55.8 | | NEURON FUNCTION | |
| Zc3h3 | ZC3H3 | | 15 | | Mus musculus zinc finger CCCH type containing 3 (Zc3h3), mRNA. | | -15.73 | | 52.2 | | TRANSCRIPTION/TRANSLATION | |
| Dhrs3 | RSDR1-PENDING | | 4 | | Mus musculus dehydrogenase/reductase (SDR family) member 3 | | -15.73 | | 11.4 | | METABOLISM, MITO FXN | |
| Spon2 | SPON2 | | 5 | | Mus musculus spondin 2, extracellular matrix protein (Spon2), mRNA. | | -15.71 | | 61.9 | | EXTRACELLULAR MATRIX | |
| Dhrs3 | DHRS3 | | 4 | | Mus musculus dehydrogenase/reductase (SDR family) member 3 (Dhrs3), mRNA. | | -15.69 | | -2.6 | | METABOLISM, MITO FXN | |
| Agpat5 | AGPAT5 | | 8 | | Mus musculus 1-acylglycerol-3-phosphate O-acyltransferase 5 (lysophosphatidic acid acyltransferase, epsilon) (Agpat5), mRNA. | | -15.67 | | 62.6 | | METABOLISM, MITO FXN | |
| Slc25a33 | SLC25A33 | | 4 | | Mus musculus solute carrier family 25, member 33 (Slc25a33), mRNA. | | -15.61 | | 36.9 | | METABOLISM, MITO FXN | |
| Scarf2 | SCARF2 | | 16 | | Mus musculus scavenger receptor class F, member 2 (Scarf2), mRNA. | | -15.55 | | 63.3 | | METABOLISM, MITO FXN | |
| Cyp4f13 | CYP4F13 | | 17 | | Mus musculus cytochrome P450, family 4, subfamily f, polypeptide 13 (Cyp4f13), mRNA. | | -15.55 | | 65.0 | | METABOLISM, MITO FXN | |
| Slc24a4 | SLC24A4 | | 12 | | Mus musculus solute carrier family 24 (sodium/potassium/calcium exchanger), member 4 | | -15.54 | | 46.1 | | TRANSMEMBRANE TRANSPORT | |
| Chrnb1 | CHRNB1 | | 11 | | Mus musculus cholinergic receptor, nicotinic, beta polypeptide 1 (muscle) (Chrnb1), mRNA. | | -15.44 | | 50.9 | | SYNAPTIC/SECRETORY FUNCTION | |
| D030026J11Rik | D030026J11RIK | |  | |  | | -15.39 | | 43.0 | | UNKNOWN GENE | |
| B4galt1 | B4GALT1 | | 4 | | Mus musculus UDP-Gal:betaGlcNAc beta 1,4- galactosyltransferase, polypeptide 1 | | -15.36 | | 34.5 | | METABOLISM, MITO FXN | |
| Grina | GRINA | | 15 | | Mus musculus glutamate receptor, ionotropic, N-methyl D-aspartate-associated protein 1 (glutamate binding) (Grina), mRNA. | | -15.34 | | 67.7 | | SYNAPTIC/SECRETORY FUNCTION | |
| Pitpnm3 | A330068P14RIK | | 11 | | Mus musculus PITPNM family member 3 | | -15.34 | | 11.3 | | PHOTORECEPTOR GENE | |
| Col23a1 | COL23A1 | | 11 | | Mus musculus collagen, type XXIII, alpha 1 | | -15.33 | | 63.3 | | EXTRACELLULAR MATRIX | |
| Ccdc86 | CCDC86 | | 19 | | Mus musculus coiled-coil domain containing 86 (Ccdc86), mRNA. | | -15.23 | | 64.8 | | TRANSCRIPTION/TRANSLATION | |
| Otud7b | OTUD7B | | 3 | | Mus musculus OTU domain containing 7B (Otud7b), transcript variant 1, mRNA. | | -15.21 | | 28.7 | | CILIA COMPONENT/FUNCTION | |
| D4Bwg0951e | D4BWG0951E | | 4 | | Mus musculus DNA segment, Chr 4, Brigham & Women's Genetics 0951 expressed (D4Bwg0951e), mRNA. | | -15.20 | | 56.1 | | UNKNOWN FUNCTION | |
| Dyrk2 | DYRK2 | | 10 | | Mus musculus dual-specificity tyrosine-(Y)-phosphorylation regulated kinase 2 (Dyrk2), mRNA. | | -15.17 | | 19.6 | | DNA DAMAGE/REPAIR | |
| Col5a1 | COL5A1 | | 2 | | Mus musculus procollagen, type V, alpha 1 (Col5a1), mRNA. | | -15.14 | | 55.5 | | EXTRACELLULAR MATRIX | |
| Cckbr | CCKBR | | 7 | | Mus musculus cholecystokinin B receptor (Cckbr), mRNA. | | -15.12 | | 61.4 | | METABOLISM, MITO FXN | |
| Kcnk1 | KCNK1 | | 8 | | Mus musculus potassium channel, subfamily K, member 1 (Kcnk1), mRNA. | | -15.04 | | 64.7 | | INTRACELLULAR TRANSPORT | |
| Ppp1r3c | PPP1R3C | | 19 | | Mus musculus protein phosphatase 1, regulatory (inhibitor) subunit 3C (Ppp1r3c), mRNA. | | -15.02 | | 55.6 | | METABOLISM, MITO FXN | |
| Wnt9b | WNT9B | | 11 | | Mus musculus wingless-type MMTV integration site 9B (Wnt9b), mRNA. | | -14.98 | | 13.5 | | CELL-CELL COMMUNICATION | |
| Pnpla3 | PNPLA3 | | 15 | | Mus musculus patatin-like phospholipase domain containing 3 (Pnpla3), mRNA. | | -14.95 | | 36.5 | | METABOLISM, MITO FXN | |
| Fezf2 | ZFP312 | | 14 | | Mus musculus Fez family zinc finger 2 | | -14.95 | | 66.3 | | NEURON FUNCTION | |
| 9130223C08Rik | 9130223C08RIK | | 4 | | Mus musculus RIKEN cDNA 9130223C08 gene | | -14.86 | | 32.5 | | UNKNOWN FUNCTION | |
| Nptx2 | NPTX2 | | 5 | | Mus musculus neuronal pentraxin 2 (Nptx2), mRNA. | | -14.86 | | 65.0 | | SYNAPTIC/SECRETORY FUNCTION | |
| Rtn4rl2 | RTN4RL2 | | 2 | | Mus musculus reticulon 4 receptor-like 2 (Rtn4rl2), mRNA. | | -14.81 | | 26.9 | | NEURON FUNCTION | |
| Nrcam | NRCAM | | 12 | | Mus musculus neuron-glia-CAM-related cell adhesion molecule (Nrcam), mRNA. | | -14.75 | | 65.0 | | NEURON FUNCTION | |
| Flcn | FLCN | | 11 | | Mus musculus folliculin (Flcn), mRNA. | | -14.74 | | 59.2 | | METABOLISM, MITO FXN | |
| Insm1 | INSM1 | | 2 | | Mus musculus insulinoma-associated 1 (Insm1), mRNA. | | -14.67 | | 54.3 | | TRANSCRIPTION/TRANSLATION | |
| Pcbp4 | PCBP4 | | 9 | | Mus musculus poly(rC) binding protein 4 (Pcbp4), mRNA. | | -14.65 | | 62.6 | | DNA DAMAGE/REPAIR | |
| AW549877 | AW549877 | | 15 | | Mus musculus chromosome 5 open reading frame 51expressed sequence AW549877 (AW549877), mRNA. | | -14.64 | | 68.1 | | UNKNOWN FUNCTION | |
| Lrig1 | LRIG1 | | 6 | | Mus musculus leucine-rich repeats and immunoglobulin-like domains 1 (Lrig1), mRNA. | | -14.61 | | 60.7 | | UNKNOWN FUNCTION | |
| Mbnl1 | MBNL1 | | 3 | | Mus musculus muscleblind-like 1 (Drosophila) (Mbnl1), mRNA. | | -14.60 | | 48.6 | | TRANSCRIPTION/TRANSLATION | |
| Pik3r1 | PIK3R1 | | 13 | | Mus musculus phosphatidylinositol 3-kinase, regulatory subunit, polypeptide 1 (p85 alpha) (Pik3r1), transcript variant 2, mRNA. | | -14.59 | | 54.7 | | METABOLISM, MITO FXN | |
| Ccdc113 | CCDC113 | | 8 | | Mus musculus coiled-coil domain containing 113 (Ccdc113), mRNA. | | -14.59 | | 35.7 | | UNKNOWN FUNCTION | |
| Trim36 | TRIM36 | | 18 | | Mus musculus tripartite motif-containing 36 (Trim36), mRNA. | | -14.52 | | 39.4 | | METABOLISM, MITO FXN | |
| Slc25a35 | SLC25A35 | | 11 | | Mus musculus solute carrier family 25, member 35 (Slc25a35), mRNA. | | -14.45 | | 42.7 | | METABOLISM, MITO FXN | |
| Frat2 | FRAT2 | | 19 | | Mus musculus frequently rearranged in advanced T-cell lymphomas 2 (Frat2), mRNA. | | -14.40 | | 65.4 | | INTRACELLULAR SIGNALING | |
| Prmt7 | PRMT7 | | 8 | | Mus musculus protein arginine N-methyltransferase 7 (Prmt7), mRNA. | | -14.16 | | 65.9 | | DNA DAMAGE/REPAIR | |
| Adam32 | ADAM32 | | 8 | | Mus musculus a disintegrin and metallopeptidase domain 32 (Adam32), mRNA. | | -14.13 | | 36.5 | | UNKNOWN FUNCTION | |
| Slc6a1 | SLC6A1 | | 6 | | Mus musculus solute carrier family 6 (neurotransmitter transporter, GABA), member 1 (Slc6a1), mRNA. XM_976103 | | -14.02 | | 56.3 | | SYNAPTIC/SECRETORY FUNCTION | |
| LOC382814 | LOC382814 | |  | |  | | -13.95 | | 39.8 | | OTHER | |
| Camkk1 | CAMKK1 | | 11 | | Mus musculus calcium/calmodulin-dependent protein kinase kinase 1, alpha (Camkk1), mRNA. | | -13.93 | | 47.1 | | METABOLISM, MITO FXN | |
| B230387C07Rik | B230387C07RIK | |  | |  | | -13.93 | | 62.9 | | UNKNOWN GENE | |
| LOC667337 | LOC667337 | |  | | PREDICTED: Mus musculus hypothetical LOC667337 (LOC667337), mRNA. | | -13.91 | | 28.5 | | OTHER | |
| Slc38a6 | SLC38A6 | | 12 | | PREDICTED: Mus musculus solute carrier family 38, member 6 (Slc38a6), mRNA. | | -13.87 | | 46.5 | | TRANSMEMBRANE TRANSPORT | |
| Rph3a | RPH3A | | 5 | | Mus musculus rabphilin 3A (Rph3a), mRNA. | | -13.84 | | 61.8 | | SYNAPTIC/SECRETORY FUNCTION | |
| scl0002064.1_2 | SCL0002064.1_2 | |  | |  | | -13.83 | | 52.2 | | UNKNOWN GENE | |
| Hook3 | HOOK3 | | 8 | | Mus musculus hook homolog 3 (Drosophila) | | -13.82 | | 38.4 | | INTRACELLULAR TRANSPORT | |
| Bin1 | BIN1 | | 18 | | Mus musculus bridging integrator 1 (Bin1), mRNA. | | -13.82 | | 58.1 | | SYNAPTIC/SECRETORY FUNCTION | |
| Gpc3 | GPC3 | | X | | Mus musculus glypican 3 (Gpc3), mRNA. | | -13.81 | | 54.0 | | EXTRACELLULAR MATRIX | |
| Hmgn1 | HMGN1 | | 16 | | Mus musculus high mobility group nucleosomal binding domain 1 | | -13.75 | | 62.6 | | TRANSCRIPTION/TRANSLATION | |
| Fam110a | FAM110A | | 2 | | Mus musculus family with sequence similarity 110, member A (Fam110a), transcript variant 1, mRNA. | | -13.73 | | 53.3 | | UNKNOWN FUNCTION | |
| Syt1 | SYT1 | | 10 | | Mus musculus synaptotagmin I | | -13.72 | | 66.6 | | SYNAPTIC/SECRETORY FUNCTION | |
| Gpsm1 | GPSM1 | |  | | Mus musculus G-protein signalling modulator 1 (AGS3-like, C. elegans) (Gpsm1), mRNA. | | -13.67 | | 58.2 | | INTRACELLULAR SIGNAL TRANSDUCTION | |
| Lgmn | LGMN | | 12 | | Mus musculus legumain (Lgmn), mRNA. | | -13.65 | | 49.7 | | METABOLISM, MITO FXN | |
| Prickle2 | 6230400G14RIK | | 6 | | Mus musculus prickle homolog 2 | | -13.63 | | 67.3 | | CILIA COMPONENT/FUNCTION | |
| Slc18a3 | SLC18A3 | | 14 | | Mus musculus solute carrier family 18 (vesicular monoamine), member 3 (Slc18a3), mRNA. | | -13.58 | | 56.1 | | NEURON FUNCTION | |
| Ndrg1 | NDRL | | 15 | | Mus musculus N-myc downstream regulated gene 1 | | -13.57 | | 51.9 | | METABOLISM, MITO FXN | |
| Ntng2 | 2610016D08RIK | | 17 | | Mus musculus netrin G2 | | -13.54 | | 22.6 | | NEURON FUNCTION | |
| Scara3 | SCARA3 | |  | | Mus musculus scavenger receptor class A, member 3 (Scara3), mRNA. | | -13.51 | | 55.8 | | METABOLISM, MITO FXN | |
| scl000951.1_9 | SCL000951.1_9 | |  | |  | | -13.47 | | 7.0 | | UNKNOWN GENE | |
| Pskh1 | PSKH1 | | 8 | | Mus musculus protein serine kinase H1 (Pskh1), mRNA. | | -13.43 | | 67.7 | | METABOLISM, MITO FXN | |
| 1520402A20Rik | 1520402A20RIK | |  | |  | | -13.34 | | 57.3 | | UNKNOWN GENE | |
| Ttyh1 | TTYH1 | | 7 | | Mus musculus tweety homolog 1 (Drosophila) | | -13.32 | | 62.3 | | METABOLISM, MITO FXN | |
| Ganc | GANC | | 2 | | Mus musculus glucosidase, alpha; neutral C (Ganc), mRNA. | | -13.32 | | 54.7 | | METABOLISM, MITO FXN | |
| Stk36 | STK36 | | 1 | | Mus musculus serine/threonine kinase 36 (fused homolog, Drosophila) (Stk36), mRNA. | | -13.23 | | 54.3 | | CILIA COMPONENT/FUNCTION | |
| LOC100045967 | LOC100045967 | |  | | PREDICTED: Mus musculus hypothetical protein LOC100045967 (LOC100045967), misc RNA. | | -13.21 | | 65.3 | | OTHER | |
| Slc19a1 | SLC19A1 | | 10 | | Mus musculus solute carrier family 19 (sodium/hydrogen exchanger), member 1 (Slc19a1), mRNA. | | -13.21 | | 58.5 | | TRANSMEMBRANE TRANSPORT | |
| Zfp787 | ZFP787 | | 7 | | Mus musculus zinc finger protein 787 (Zfp787), mRNA. | | -13.18 | | 60.7 | | TRANSCRIPTION/TRANSLATION | |
| Zfp238 | ZFP238 | | 1 | | Mus musculus zinc finger protein 238 (Zfp238), transcript variant 1, mRNA. | | -13.16 | | 60.8 | | TRANSCRIPTION/TRANSLATION | |
| Gtf2f2 | GTF2F2 | | 14 | | Mus musculus general transcription factor IIF, polypeptide 2 (Gtf2f2), mRNA. | | -13.15 | | 53.3 | | TRANSCRIPTION/TRANSLATION | |
| Chrna6 | CHRNA6 | | 8 | | Mus musculus cholinergic receptor, nicotinic, alpha polypeptide 6 (Chrna6), mRNA. | | -13.12 | | 66.0 | | SYNAPTIC/SECRETORY FUNCTION | |
| LOC677213 | LOC677213 | |  | | PREDICTED: Mus musculus similar to U2AF homology motif (UHM) kinase 1 (LOC677213), mRNA. | | -13.06 | | 60.7 | | OTHER | |
