# Supplementary material for: Transcription Coactivators p300 and CBP Are Necessary for Photoreceptor-Specific Chromatin Organization and Gene Expression
Source: PLoS One. 2013 Jul 26;8(7):e69721. doi: 10.1371/journal.pone.0069721 (PMC3724885; doi:10.1371/journal.pone.0069721)
Supplement: Table S3 — 520 genes down-regulated in R-DCKO vs Cre negative. (DOCX) [file pone.0069721.s008.docx]

| **Table S3. 579 genes up-regulated in *R-DCKO* vs *Cre negative*** | | | | |  |  |
| --- | --- | --- | --- | --- | --- | --- |
| **SYMBOL** | **ILMN_GENE** | **CHROM-OSOME** | **DEFINITION** | **R-DCKO DIFF SCORE** | **%**  **CRE NEG** | **CELL PROCESS** |
| Uchl1 | UCHL1 | 5 | Mus musculus ubiquitin carboxy-terminal hydrolase L1 (Uchl1), mRNA. | 13.06 | 139.31 | NEURONAL FXN |
| Pex11b | PEX11B | 3 | Mus musculus peroxisomal biogenesis factor 11b (Pex11b), mRNA. | 13.15 | 158.50 | METABOLISM/MITO FXN |
| Slc35b2 | SLC35B2 | 17 | Mus musculus solute carrier family 35, member B2 (Slc35b2), mRNA. | 13.15 | 161.18 | TRANSMEMBRANE TRANSPORT |
| Med22 | MED22 | 2 | Mus musculus mediator complex subunit 22 (Med22), transcript variant 1, mRNA. | 13.20 | 157.33 | TRANSCRIPTION/TRANSLATION |
| Bahd1 | BAHD1 | 2 | Mus musculus bromo adjacent homology domain containing 1 (Bahd1), mRNA. | 13.22 | 166.57 | CHROMATIN |
| Nudt16l1 | NUDT16L1 | 16 | Mus musculus nudix (nucleoside diphosphate linked moiety X)-type motif 16-like 1 (Nudt16l1), mRNA. | 13.22 | 144.09 | UNKNOWN FUNCTION |
| Pnma2 | PNMA2 | 14 | Mus musculus paraneoplastic antigen MA2 (Pnma2), mRNA. | 13.26 | 180.13 | APOPTOSIS |
| Lin54 | LIN54 | 5 | Mus musculus lin-54 homolog (C. elegans) (Lin54), mRNA. | 13.29 | 145.54 | TRANSCRIPTION/TRANSLATION |
| Marcks | MARCKS | 10 | Mus musculus myristoylated alanine rich protein kinase C substrate (Marcks), mRNA. | 13.33 | 139.40 | CYTOSKELETAL/NUCLEAR ENVELOPE |
| Phpt1 | PHPT1 | 2 | Mus musculus phosphohistidine phosphatase 1 (Phpt1), mRNA. | 13.33 | 139.73 | METABOLISM/MITO FXN |
| Anapc5 | ANAPC5 | 5 | Mus musculus anaphase-promoting complex subunit 5 (Anapc5), transcript variant 1, mRNA. | 13.34 | 155.51 | CELL CYCLE |
| Tmem55b | TMEM55B | 14 | Mus musculus transmembrane protein 55b (Tmem55b), mRNA. XM_919952 XM_919965 | 13.34 | 132.69 | METABOLISM/MITO FXN |
| Hist1h2bf | HIST1H2BF | 13 | Mus musculus histone cluster 1, H2bf (Hist1h2bf), mRNA. | 13.37 | 292.43 | CHROMATIN |
| 2310047K21Rik | 2310047K21RIK | 2 |  | 13.37 | 152.20 | UNKNOWN FUNCTION |
| Mrpl3 | MRPL3 | 9 | Mus musculus mitochondrial ribosomal protein L3 (Mrpl3), nuclear gene encoding mitochondrial protein, mRNA. | 13.37 | 157.75 | METABOLISM/MITO FXN |
| 2810025M15Rik | 2810025M15RIK | 1 | PREDICTED: Mus musculus RIKEN cDNA 2810025M15 gene (2810025M15Rik), misc RNA. | 13.38 | 140.77 | UNKNOWN FUNCTION |
| Cgrrf1 | CGRRF1 | 14 | Mus musculus cell growth regulator with ring finger domain 1 (Cgrrf1), mRNA. | 13.38 | 146.94 | CELL CYCLE |
| Tapbpl | TAPBPL | 6 | Mus musculus TAP binding protein-like (Tapbpl), mRNA. | 13.40 | 318.03 | TRANSMEMBRANE TRANSPORT |
| Sepn1 | SEPN1 | 4 | Mus musculus selenoprotein N, 1 (Sepn1), mRNA. | 13.42 | 140.22 | UNKNOWN FUNCTION |
| Ccdc45 | F630025I20RIK | 11 | Mus musculus coiled-coil domain containing 45 | 13.46 | 143.19 | CYTOSKELETAL/NUCLEAR ENVELOPE |
| Ercc1 | ERCC1 | 7 | Mus musculus excision repair cross-complementing rodent repair deficiency, complementation group 1 (Ercc1), mRNA. | 13.46 | 150.83 | DNA REPAIR/REPLICATION |
| LOC383981 | LOC383981 |  |  | 13.57 | 194.18 | OTHER |
| Men1 | MEN1 | 19 | Mus musculus multiple endocrine neoplasia 1 (Men1), mRNA. | 13.58 | 155.06 | TRANSCRIPTION/TRANSLATION |
| Pcbd2 | PCBD2 | 13 | Mus musculus pterin 4 alpha carbinolamine dehydratase/dimerization cofactor of hepatocyte nuclear factor 1 alpha (TCF1) 2 (Pcbd2), mRNA. | 13.59 | 176.24 | METABOLISM/MITO FXN |
| Mcm6 | MCM6 | 1 | Mus musculus minichromosome maintenance deficient 6 (MIS5 homolog, S. pombe) (S. cerevisiae) (Mcm6), mRNA. | 13.63 | 319.73 | CELL CYCLE |
| Dpm3 | DPM3 | 3 | Mus musculus dolichyl-phosphate mannosyltransferase polypeptide 3 | 13.68 | 147.93 | METABOLISM/MITO FXN |
| Mrps16 | MRPS16 | 14 | Mus musculus mitochondrial ribosomal protein S16 (Mrps16), nuclear gene encoding mitochondrial protein, mRNA. | 13.70 | 143.79 | METABOLISM/MITO FXN |
| Nudt2 | NUDT2 | 4 | Mus musculus nudix (nucleoside diphosphate linked moiety X)-type motif 2 (Nudt2), mRNA. | 13.71 | 196.46 | METABOLISM/MITO FXN |
| Camk1 | CAMK1 | 6 | Mus musculus calcium/calmodulin-dependent protein kinase I (Camk1), mRNA. | 13.72 | 228.10 | SYNAPTIC FUNCTION |
| Pgls | PGLS | 8 | Mus musculus 6-phosphogluconolactonase | 13.74 | 183.35 | METABOLISM/MITO FXN |
| Churc1 | CHURC1 | 12 | Mus musculus churchill domain containing 1 | 13.74 | 166.80 | TRANSCRIPTION/TRANSLATION |
| 2510002D24Rik | 2510002D24RIK | 16 | Mus musculus RIKEN cDNA 2510002D24 gene (2510002D24Rik), mRNA. XM_922927 | 13.78 | 159.77 | UNKNOWN FUNCTION |
| Fbxo16 | FBXO16 | 14 | Mus musculus F-box protein 16 (Fbxo16), mRNA. | 13.84 | 174.92 | METABOLISM/MITO FXN |
| Gtf3c5 | GTF3C5 | 2 | Mus musculus general transcription factor IIIC, polypeptide 5 (Gtf3c5), mRNA. | 13.85 | 167.34 | TRANSCRIPTION/TRANSLATION |
| Ddx47 | DDX47 | 6 | Mus musculus DEAD (Asp-Glu-Ala-Asp) box polypeptide 47 (Ddx47), mRNA. | 13.85 | 145.46 | METABOLISM/MITO FXN |
| Dpp9 | DPP9 | 17 | Mus musculus dipeptidylpeptidase 9 (Dpp9), mRNA. | 13.88 | 176.48 | METABOLISM/MITO FXN |
| Uxt | UXT | X | Mus musculus ubiquitously expressed transcript (Uxt), mRNA. | 13.89 | 196.68 | TRANSCRIPTION/TRANSLATION |
| C1galt1 | 2210410E06RIK | 6 | Mus musculus core 1 synthase, glycoprotein-N-acetylgalactosamine 3-beta-galactosyltransferase, 1 | 13.91 | 146.70 | METABOLISM/MITO FXN |
| Kat2a | KAT2A | 11 | Mus musculus K(lysine) acetyltransferase 2A (Kat2a), transcript variant 2, mRNA. | 13.92 | 150.14 | CHROMATIN |
| Rpp25l | 2810432D09RIK | 4 | Mus musculus ribonuclease P/MRP 25 subunit-like; RIKEN cDNA 2810432D09 gene (2810432D09Rik), mRNA. | 13.92 | 182.61 | METABOLISM/MITO FXN |
| Cars2 | CARS2 | 8 | PREDICTED: Mus musculus cysteinyl-tRNA synthetase 2 (mitochondrial)(putative) (Cars2), mRNA. | 13.93 | 173.18 | METABOLISM/MITO FXN |
| Topors | TOPORS | 4 | Mus musculus topoisomerase I binding, arginine/serine-rich (Topors), mRNA. | 13.93 | 147.45 | RETINAL DISEASE GENE |
| Bbx | BBX | 16 | Mus musculus bobby sox homolog (Drosophila) | 13.93 | 193.17 | TRANSCRIPTION/TRANSLATION |
| Zfp551 | ZFP551 | 7 | Mus musculus zinc fingr protein 551 (Zfp551), mRNA. XM_921380 XM_921386 XM_921391 XM_921394 | 13.95 | 153.66 | TRANSCRIPTION/TRANSLATION |
| Tmem101 | TMEM101 | 11 | Mus musculus transmembrane protein 101 (Tmem101), mRNA. | 13.96 | 147.22 | CELL-CELL COMMUNICATION |
| Tceal1 | TCEAL1 | X | Mus musculus transcription elongation factor A (SII)-like 1 (Tceal1), mRNA. | 14.00 | 154.21 | TRANSCRIPTION/TRANSLATION |
| Sft2d3 | SFT2D3 | 18 | PREDICTED: Mus musculus SFT2 domain containing 3 (Sft2d3), mRNA. | 14.01 | 168.63 | INTRACELLULAR TRANSPORT |
| Stk11 | STK11 | 10 | Mus musculus serine/threonine kinase 11 (Stk11), mRNA. | 14.02 | 144.61 | METABOLISM/MITO FXN |
| Uap1l1 | UAP1L1 | 2 | Mus musculus UDP-N-acteylglucosamine pyrophosphorylase 1-like 1 (Uap1l1), mRNA. XM_918982 | 14.02 | 176.99 | METABOLISM/MITO FXN |
| 1810037C20Rik | 1810037C20RIK | X | Mus musculus RIKEN cDNA 1810037C20 gene (1810037C20Rik), mRNA. | 14.07 | 142.12 | UNKNOWN FUNCTION |
| Dis3l2 | DIS3L2 | 1 | Mus musculus DIS3 mitotic control homolog (S. cerevisiae)-like 2 (Dis3l2), mRNA. | 14.13 | 148.19 | CELL CYCLE |
| Cenpw | 2610036L11RIK | 10 | Mus musculus centromere protein W; RIKEN cDNA 2610036L11Rik | 14.15 | 191.83 | CELL CYCLE |
| Lyrm2 | LYRM2 | 4 | Mus musculus LYR motif containing 2 (Lyrm2), mRNA. | 14.17 | 165.55 | METABOLISM/MITO FXN |
| LOC668573 | LOC668573 | 17 | PREDICTED: Mus musculus similar to ribosomal protein (LOC668573), misc RNA. | 14.19 | 140.31 | OTHER |
| Nasp | NASP | 4 | Mus musculus nuclear autoantigenic sperm protein (histone-binding) (Nasp), transcript variant 2, mRNA. | 14.30 | 195.92 | CELL CYCLE |
| scl000710.1_2295 | SCL000710.1_2295 |  |  | 14.33 | 149.89 | UNKNOWN GENE |
| Fahd2a | FAHD2A | 2 | Mus musculus fumarylacetoacetate hydrolase domain containing 2A (Fahd2a), mRNA. | 14.37 | 175.73 | METABOLISM/MITO FXN |
| Fxc1 | FXC1 | 7 | Mus musculus fractured callus expressed transcript 1 (Fxc1), mRNA. | 14.39 | 157.25 | METABOLISM/MITO FXN |
| Krtcap2 | KRTCAP2 | 3 | Mus musculus keratinocyte associated protein 2 (Krtcap2), mRNA. | 14.39 | 146.04 | METABOLISM/MITO FXN |
| Xab1 | XAB1 | 5 | Mus musculus XPA binding protein 1 (Xab1), mRNA. | 14.41 | 142.78 | INTRACELLULAR SIGNAL TRANSDUCTION |
| Msto1 | MSTO1 | 3 | Mus musculus misato homolog 1 (Drosophila) (Msto1), mRNA. | 14.42 | 174.16 | METABOLISM/MITO FXN |
| Nln | NLN | 13 | Mus musculus neurolysin (metallopeptidase M3 family) (Nln), mRNA. | 14.45 | 177.82 | METABOLISM/MITO FXN |
| 2700094K13Rik | 2700094K13RIK | 2 | Mus musculus RIKEN cDNA 2700094K13 gene (2700094K13Rik), transcript variant 2, mRNA. | 14.49 | 183.53 | METABOLISM/MITO FXN |
| Prmt3 | PRMT3 | 7 | Mus musculus protein arginine N-methyltransferase 3 (Prmt3), mRNA. | 14.53 | 143.31 | METABOLISM/MITO FXN |
| Emd | EMD | X | Mus musculus emerin (Emd), mRNA. | 14.55 | 161.88 | CYTOSKELETAL/NUCLEAR ENVELOPE |
| Fuom | 1810014F10RIK | 7 | Mus musculus fucose mutarotase; RIKEN cDNA 1810014F10 gene (1810014F10Rik), mRNA. | 14.61 | 149.44 | METABOLISM/MITO FXN |
| Bmi1 | BMI1 | 2 | Mus musculus Bmi1 polycomb ring finger oncogene (Bmi1), mRNA. | 14.64 | 160.83 | TRANSCRIPTION/TRANSLATION |
| Flywch2 | FLYWCH2 | 17 | Mus musculus FLYWCH family member 2 (Flywch2), mRNA. | 14.65 | 142.14 | UNKNOWN FUNCTION |
| Hist2h2aa1 | HIST2H2AA1 | 3 | Mus musculus histone cluster 2, H2aa1 | 14.66 | 484.84 | CHROMATIN |
| AI413582 | AI413582 | 17 | Mus musculus uncharacterized protein C6orf1 homolog | 14.69 | 168.65 | UNKNOWN FUNCTION |
| Iah1 | IAH1 | 12 | Mus musculus isoamyl acetate-hydrolyzing esterase 1 homolog (S. cerevisiae) (Iah1), mRNA. | 14.73 | 221.41 | UNKNOWN FUNCTION |
| Nsa2 | 5730427N09RIK | 13 | Mus musculus ribosome biogenesis homolog (S. cerevisiae) | 14.74 | 162.78 | CELL CYCLE |
| Med27 | MED27 | 2 | Mus musculus mediator complex subunit 27 (Med27), mRNA. | 14.75 | 145.30 | TRANSCRIPTION/TRANSLATION |
| Nipsnap3a | NIPSNAP3A | 4 | Mus musculus nipsnap homolog 3A (C. elegans) (Nipsnap3a), mRNA. | 14.80 | 297.19 | INTRACELLULAR TRANSPORT |
| Mettl2 | 2810438F06RIK | 11 | Mus musculous methyltransferase like 2 | 14.80 | 145.47 | METABOLISM/MITO FXN |
| B230317C12Rik | B230317C12RIK | 2 | Mus musculus RIKEN cDNA B230317C12 gene (B230317C12Rik), mRNA. | 14.84 | 158.49 | METABOLISM/MITO FXN |
| Cdk9 | CDK9 | 2 | Mus musculus cyclin-dependent kinase 9 (CDC2-related kinase) (Cdk9), mRNA. | 14.85 | 152.18 | TRANSCRIPTION/TRANSLATION |
| Chchd4 | CHCHD4 | 6 | Mus musculus coiled-coil-helix-coiled-coil-helix domain containing 4 (Chchd4), nuclear gene encoding mitochondrial protein, mRNA. | 14.86 | 146.76 | METABOLISM/MITO FXN |
| Rbm4b | RBM4B | 19 | Mus musculus RNA binding motif protein 4B (Rbm4b), mRNA. | 14.86 | 182.00 | TRANSCRIPTION/TRANSLATION |
| Rabep2 | RABEP2 | 7 | Mus musculus rabaptin, RAB GTPase binding effector protein 2 (Rabep2), mRNA. | 14.86 | 189.49 | CELL-CELL COMMUNICATION |
| Dennd3 | DENND3 | 15 | Mus musculus DENN/MADD domain containing 3 (Dennd3), mRNA. | 14.88 | 265.17 | METABOLISM/MITO FXN |
| Slc27a2 | SLC27A2 | 2 | Mus musculus solute carrier family 27 (fatty acid transporter), member 2 (Slc27a2), mRNA. | 14.99 | 863.83 | METABOLISM/MITO FXN |
| 1810009N02Rik | 1810009N02RIK | 12 | Mus musculus RIKEN cDNA 1810009N02 gene (1810009N02Rik), mRNA. | 15.05 | 272.66 | METABOLISM/MITO FXN |
| Zfp30 | ZFP30 | 7 | Mus musculus zinc finger protein 30 (Zfp30), mRNA. | 15.07 | 165.18 | TRANSCRIPTION/TRANSLATION |
| Slc4a2 | SLC4A2 | 5 | Mus musculus solute carrier family 4 (anion exchanger), member 2 (Slc4a2), mRNA. | 15.12 | 146.83 | METABOLISM/MITO FXN |
| Eef1e1 | EEF1E1 | 13 | Mus musculus eukaryotic translation elongation factor 1 epsilon 1 (Eef1e1), mRNA. | 15.13 | 150.73 | METABOLISM/MITO FXN |
| Phf13 | PHF13 | 4 | Mus musculus PHD finger protein 13 (Phf13), mRNA. | 15.14 | 179.74 | CHROMATIN |
| Fsd1 | FSD1 | 17 | Mus musculus fibronectin type 3 and SPRY domain-containing protein (Fsd1), mRNA. | 15.19 | 152.42 | CYTOSKELETAL/NUCLEAR ENVELOPE |
| Igf2bp3 | IGF2BP3 | 6 | Mus musculus insulin-like growth factor 2 mRNA binding protein 3 | 15.22 | 268.41 | TRANSCRIPTION/TRANSLATION |
| Ippk | IPPK | 13 | Mus musculus inositol 1,3,4,5,6-pentakisphosphate 2-kinase (Ippk), mRNA. | 15.26 | 156.83 | INTRACELLULAR SIGNAL TRANSDUCTION |
| Micu1 | CBARA1 | 10 | Mus musculus mitochondrial calcium uptake 1 (also called Cbara1) | 15.28 | 147.73 | METABOLISM/MITO FXN |
| Pbx4 | PBX4 | 8 | Mus musculus pre B cell leukemia homeobox 4 | 15.29 | 216.37 | TRANSCRIPTION/TRANSLATION |
| Ermp1 | ERMP1 | 19 | Mus musculus endoplasmic reticulum metallopeptidase 1 (Ermp1), mRNA. | 15.30 | 182.86 | METABOLISM/MITO FXN |
| LOC100044298 | LOC100044298 | 9 | PREDICTED: Mus musculus hypothetical protein LOC100044298 (LOC100044298), mRNA. | 15.30 | 183.61 | UNKNOWN FUNCTION |
| Slc46a1 | SLC46A1 | 11 | Mus musculus solute carrier family 46, member 1 (Slc46a1), mRNA. | 15.31 | 198.60 | METABOLISM/MITO FXN |
| Cenpv | 3110013H01RIK | 11 | Mus musculus centromere protein V | 15.34 | 180.32 | CELL CYCLE |
| Gpr45 | 9230112G11RIK | 1 | Mus musculus G protein-coupled receptor 45 | 15.34 | 296.12 | CELL-CELL COMMUNICATION |
| Nedd1 | NEDD1 | 10 | Mus musculus neural precursor cell expressed, developmentally down-regulated gene 1 (Nedd1), mRNA. | 15.38 | 171.91 | CELL CYCLE |
| C430002D13Rik | C430002D13RIK |  |  | 15.38 | -347.89 | UNKNOWN GENE |
| Mrps9 | MRPS9 | 1 | Mus musculus mitochondrial ribosomal protein S9 (Mrps9), mRNA. | 15.40 | 145.24 | METABOLISM/MITO FXN |
| Hmgb2l1 | HMGB2L1 | 8 | Mus musculus high mobility group box 2-like 1 (Hmgb2l1), mRNA. | 15.53 | 169.73 | CHROMATIN |
| Manea | MANEA | 4 | Mus musculus mannosidase, endo-alpha (Manea), mRNA. | 15.55 | 152.90 | METABOLISM/MITO FXN |
| Chac1 | CHAC1 | 2 | Mus musculus ChaC, cation transport regulator-like 1 (E. coli) (Chac1), mRNA. | 15.55 | 166.10 | METABOLISM/MITO FXN |
| Sun2 | B230369L08RIK | 15 | Mus musculus Sad1 and UNC84 domain containing 2 | 15.56 | 200.26 | CYTOSKELETAL/NUCLEAR ENVELOPE |
| Ppdpf | 2700038C09RIK | 2 | Mus musculus RIKEN cDNA 2700038C09 gene; pancreatic progenitor cell differentiation and proliferation factor homolog (zebrafish) | 15.57 | 172.72 | CELL-CELL COMMUNICATION |
| Pxmp4 | PXMP4 | 2 | Mus musculus peroxisomal membrane protein 4 (Pxmp4), mRNA. | 15.60 | 166.79 | METABOLISM/MITO FXN |
| Usp11 | USP11 | X | Mus musculus ubiquitin specific peptidase 11 (Usp11), mRNA. | 15.61 | 172.08 | METABOLISM/MITO FXN |
| Zfp28 | ZFP28 | 7 | Mus musculus zinc finger protein 28 (Zfp28), mRNA. | 15.65 | 184.66 | TRANSCRIPTION/TRANSLATION |
| Saysd1 | 1810063B07RIK | 14 | Mus musculus SAYSVFN motif domain containing 1 | 15.69 | 167.17 | INTRACELLULAR TRANSPORT |
| A530065H22Rik | A530065H22RIK | not found |  | 15.70 | 145.89 | UNKNOWN GENE |
| Mtvr2 | MTVR2 | 19 | Mus musculus mammary tumor virus receptor 2 (Mtvr2), transcript variant 2, mRNA. | 15.72 | 173.71 | UNKNOWN FUNCTION |
| Lmf1 | LMF1 | 17 | Mus musculus lipase maturation factor 1 (Lmf1), mRNA. | 15.77 | 171.86 | METABOLISM/MITO FXN |
| Inha | INHA | 1 | Mus musculus inhibin alpha (Inha), mRNA. | 15.78 | 177.68 | CELL-CELL COMMUNICATION |
| Rnaset2 | RNASET2 | 17 | Mus musculus ribonuclease T2 (Rnaset2), transcript variant 2, mRNA. | 15.79 | 148.13 | METABOLISM/MITO FXN |
| scl0001647.1_23 | SCL0001647.1_23 |  |  | 15.80 | 168.95 | UNKNOWN GENE |
| Ufc1 | UFC1 | 1 | Mus musculus ubiquitin-fold modifier conjugating enzyme 1 (Ufc1), mRNA. | 15.80 | 143.03 | METABOLISM/MITO FXN |
| Thap7 | THAP7 | 16 | Mus musculus THAP domain containing 7 | 15.83 | 149.82 | TRANSCRIPTION/TRANSLATION |
| Fchsd1 | FCHSD1 | 18 | Mus musculus FCH and double SH3 domains 1 (Fchsd1), mRNA. | 15.85 | 151.36 | UNKNOWN FUNCTION |
| Ccdc159 | 2510048L02RIK | 9 | Mus musculus coiled-coil domain containing 159; RIKEN cDNA 2510048L02 gene (2510048L02Rik), mRNA. | 15.89 | 508.26 | UNKNOWN FUNCTION |
| Zfp414 | ZFP414 | 17 | Mus musculus zinc finger protein 414 (Zfp414), mRNA. | 15.92 | 181.25 | TRANSCRIPTION/TRANSLATION |
| Rabac1 | RABAC1 | 7 | Mus musculus Rab acceptor 1 (prenylated) (Rabac1), mRNA. | 15.93 | 157.42 | INTRACELLULAR SIGNAL TRANSDUCTION |
| Ngdn | 1500001L15RIK | 14 | Mus musculus neuroguidin, EIF4E binding protein | 15.95 | 143.03 | TRANSCRIPTION/TRANSLATION |
| Jund1 | JUND1 | 8 | Mus musculus Jun proto-oncogene related gene d1 (Jund1), mRNA. | 16.02 | 222.38 | TRANSCRIPTION/TRANSLATION |
| Ldb1 | LDB1 | 19 | Mus musculus LIM domain binding 1 (Ldb1), transcript variant 3, mRNA. | 16.02 | 142.95 | TRANSCRIPTION/TRANSLATION |
| Pafah1b3 | PAFAH1B3 | 7 | Mus musculus platelet-activating factor acetylhydrolase, isoform 1b, alpha1 subunit (Pafah1b3), mRNA. | 16.02 | 254.08 | METABOLISM/MITO FXN |
| Tceb2 | TCEB2 | 17 | Mus musculus transcription elongation factor B (SIII), polypeptide 2 (Tceb2), mRNA. | 16.03 | 146.29 | TRANSCRIPTION/TRANSLATION |
| Tarsl2 | TARSL2 | 7 | Mus musculus threonyl-tRNA synthetase-like 2 (Tarsl2), mRNA. | 16.07 | 178.86 | METABOLISM/MITO FXN |
| Hist1h2al | EG667728 | 13 | PREDICTED: Mus musculus predicted gene, EG667728; histone cluster 1, H2al | 16.07 | 254.50 | CHROMATIN |
| Paox | PAOX | 7 | Mus musculus polyamine oxidase (exo-N4-amino) (Paox), mRNA. | 16.11 | 183.86 | METABOLISM/MITO FXN |
| Zbtb45 | ZBTB45 | 7 | Mus musculus zinc finger and BTB domain containing 45 (Zbtb45), mRNA. | 16.14 | 154.78 | TRANSCRIPTION/TRANSLATION |
| Dnlz | D2BWG1335E | 2 | Mus musculus DNL-type zinc finger; DNA segment, Chr 2, Brigham & Women's Genetics 1335 expressed (D2Bwg1335e), mRNA. | 16.18 | 242.27 | METABOLISM/MITO FXN |
| Bad | BAD | 19 | Mus musculus BCL2-associated agonist of cell death (Bad), mRNA. | 16.18 | 149.50 | APOPTOSIS |
| Scrn2 | SCRN2 | 11 | Mus musculus secernin 2 (Scrn2), mRNA. | 16.19 | 184.20 | METABOLISM/MITO FXN |
| Stambp | STAMBP | 6 | Mus musculus Stam binding protein (Stambp), mRNA. | 16.21 | 146.05 | INTRACELLULAR SIGNAL TRANSDUCTION |
| Mgst3 | MGST3 | 1 | Mus musculus microsomal glutathione S-transferase 3 (Mgst3), mRNA. | 16.22 | 157.05 | METABOLISM/MITO FXN |
| Ppp4r1l-ps | 1700007P14RIK | 2 | Mus musculus protein phosphatase 4, regulatory subunit 1-like, pseudogene | 16.30 | 275.03 | OTHER |
| Ppil3 | PPIL3 | 1 | Mus musculus peptidylprolyl isomerase (cyclophilin)-like 3 (Ppil3), transcript variant 1, mRNA. | 16.33 | 191.88 | TRANSCRIPTION/TRANSLATION |
| Tsen34 | TSEN34 | 7 | Mus musculus tRNA splicing endonuclease 34 homolog (S. cerevisiae) (Tsen34), mRNA. | 16.36 | 149.62 | TRANSCRIPTION/TRANSLATION |
| LOC381114 | LOC381114 |  |  | 16.40 | 143.09 | OTHER |
| Stard6 | STARD6 | 18 | Mus musculus StAR-related lipid transfer (START) domain containing 6 (Stard6), mRNA. | 16.41 | 285.30 | METABOLISM/MITO FXN |
| Rrp15 | RRP15 | 1 | Mus musculus ribosomal RNA processing 15 homolog (S. cerevisiae) (Rrp15), mRNA. | 16.48 | 158.57 | METABOLISM/MITO FXN |
| Cenpt | CENPT | 8 | Mus musculus centromere protein T (Cenpt), mRNA. | 16.50 | 148.20 | CELL CYCLE |
| Tbc1d25 | TBC1D25 | X | Mus musculus TBC1 domain family, member 25 (Tbc1d25), mRNA. | 16.54 | 162.80 | INTRACELLULAR SIGNAL TRANSDUCTION |
| scl0002337.1_39 | SCL0002337.1_39 |  |  | 16.55 | 173.91 | UNKNOWN GENE |
| Hist1h3f | HIST1H3F | 13 | Mus musculus histone cluster 1, H3f (Hist1h3f), mRNA. | 16.56 | 324.08 | CHROMATIN |
| Scotin | SCOTIN | 9 | Mus musculus scotin gene (Scotin), transcript variant 1, mRNA. | 16.57 | 183.21 | APOPTOSIS |
| Cmc1 | CMC1 | 9 | Mus musculus COX assembly mitochondrial protein homolog (S. cerevisiae) (Cmc1), nuclear gene encoding mitochondrial protein, mRNA. | 16.60 | 145.49 | METABOLISM/MITO FXN |
| Atp5j | ATP5J | 16 | Mus musculus ATP synthase, H+ transporting, mitochondrial F0 complex, subunit F (Atp5j), nuclear gene encoding mitochondrial protein, mRNA. | 16.61 | 105.25 | METABOLISM/MITO FXN |
| 1110002D22Rik | 1110002D22RIK | not found |  | 16.62 | 145.95 | UNKNOWN GENE |
| Bhlhb9 | BHLHB9 | X | Mus musculus basic helix-loop-helix domain containing, class B9 (Bhlhb9), mRNA. | 16.64 | 143.99 | TRANSCRIPTION/TRANSLATION |
| B4galt3 | B4GALT3 | 1 | Mus musculus UDP-Gal:betaGlcNAc beta 1,4-galactosyltransferase, polypeptide 3 (B4galt3), mRNA. | 16.68 | 162.64 | METABOLISM/MITO FXN |
| LOC634015 | LOC634015 | 16 | PREDICTED: Mus musculus similar to Proteasome subunit beta type 3 (Proteasome theta chain) (Proteasome chain 13) (Proteasome component C10-II) (LOC634015), mRNA. | 16.70 | 143.91 | OTHER |
| Prdx3 | PRDX3 | 19 | Mus musculus peroxiredoxin 3 | 16.77 | 146.16 | METABOLISM/MITO FXN |
| Cklf | CKLF | 8 | Mus musculus chemokine-like factor (Cklf), transcript variant 1, mRNA. | 16.80 | 327.21 | CELL-CELL COMMUNICATION |
| Psip1 | PSIP1 | 4 | Mus musculus PC4 and SFRS1 interacting protein 1 (Psip1), mRNA. | 16.86 | 153.71 | TRANSCRIPTION/TRANSLATION |
| Gatad1 | GATAD1 | 5 | Mus musculus GATA zinc finger domain containing 1 (Gatad1), mRNA. | 16.91 | 164.02 | TRANSCRIPTION/TRANSLATION |
| Erh | ERH | 12 | Mus musculus enhancer of rudimentary homolog (Drosophila) (Erh), mRNA. | 16.92 | 145.90 | METABOLISM/MITO FXN |
| Snapin | SNAPIN | 3 | Mus musculus SNAP-associated protein (Snapin), mRNA. | 16.94 | 164.35 | INTRACELLULAR TRANSPORT |
| Map4k2 | MAP4K2 | 19 | Mus musculus mitogen-activated protein kinase kinase kinase kinase 2 (Map4k2), mRNA. | 16.95 | 155.65 | INTRACELLULAR SIGNAL TRANSDUCTION |
| LOC674427 | LOC674427 |  | PREDICTED: Mus musculus similar to ribosomal protein L7a (LOC674427), misc RNA. | 17.03 | 244.15 | OTHER |
| Nubp1 | NUBP1 | 16 | Mus musculus nucleotide binding protein 1 | 17.09 | 178.03 | METABOLISM/MITO FXN |
| Hist1h2bc | HIST1H2BC | 13 | Mus musculus histone cluster 1, H2bc (Hist1h2bc), mRNA. | 17.11 | 251.01 | CHROMATIN |
| Rnaseh2b | 2610207P08RIK | 14 | Mus musculus ribonuclease H2, subunit B | 17.14 | 154.58 | CELL CYCLE |
| Erich1 | ERICH1 | 8 | Mus musculus glutamate-rich 1 (Erich1), mRNA. | 17.15 | 152.53 | UNKNOWN FUNCTION |
| Ier3ip1 | IER3IP1 | 18 | Mus musculus immediate early response 3 interacting protein 1 (Ier3ip1), mRNA. | 17.17 | 145.05 | METABOLISM/MITO FXN |
| Rps6kl1 | RPS6KL1 | 12 | Mus musculus ribosomal protein S6 kinase-like 1 (Rps6kl1), mRNA. | 17.17 | 177.27 | METABOLISM/MITO FXN |
| LOC637353 | LOC637353 | 4 | PREDICTED: Mus musculus similar to Anp32b protein (LOC637353), mRNA. | 17.20 | 183.56 | OTHER |
| Pgcp | PGCP | 15 | Mus musculus plasma glutamate carboxypeptidase (Pgcp), transcript variant 2, mRNA. | 17.20 | 172.84 | METABOLISM/MITO FXN |
| Rpain | 2400006N03RIK | 11 | Mus musculus RPA interacting protein | 17.21 | 177.28 | DNA REPAIR/REPLICATION |
| Ard1a | ARD1A | X | Mus musculus ARD1 homolog A, N-acetyltransferase (S. cerevisiae) (Ard1a), mRNA. | 17.28 | 160.15 | METABOLISM/MITO FXN |
| E430018J23Rik | E430018J23RIK | 7 | Mus musculus RIKEN cDNA E430018J23 gene (E430018J23Rik), mRNA. | 17.30 | 181.27 | UNKNOWN FUNCTION |
| D17Wsu104e | D17WSU104E | 17 | Mus musculus DNA segment, Chr 17, Wayne State University 104, expressed | 17.30 | 151.77 | METABOLISM/MITO FXN |
| Atraid | 0610007C21RIK | 5 | Mus musculus Atraid all-trans retinoic acid induced differentiation factor;RIKEN cDNA 0610007C21 gene (0610007C21Rik), transcript variant 2, mRNA. | 17.31 | 144.91 | APOPTOSIS |
| LOC665250 | LOC665250 | 11 | PREDICTED: Mus musculus similar to RNA polymerase II transcription factor SIII p18 subunit (LOC665250), misc RNA. | 17.32 | 144.09 | OTHER |
| LOC100046163 | LOC100046163 |  | PREDICTED: Mus musculus similar to Nme6 protein (LOC100046163), mRNA. | 17.34 | 202.64 | OTHER |
| LOC230765 | LOC230765 | 12 | PREDICTED: Mus musculus histone deacetylase 1 pseudogene | 17.37 | 176.85 | OTHER |
| 2610037P13Rik | 2610037P13RIK | 17 | Mus musculus RIKEN cDNA 2610037P13 gene | 17.41 | 151.67 | UNKNOWN FUNCTION |
| Ccdc111 | CCDC111 | 8 | Mus musculus coiled-coil domain containing 111 (Ccdc111), mRNA. | 17.48 | 188.62 | DNA REPAIR/REPLICATION |
| Spcs3 | SPCS3 | 8 | Mus musculus signal peptidase complex subunit 3 homolog (S. cerevisiae) (Spcs3), mRNA. | 17.54 | 177.05 | METABOLISM/MITO FXN |
| Pycrl | PYCRL | 15 | Mus musculus pyrroline-5-carboxylate reductase-like | 17.65 | 155.81 | METABOLISM/MITO FXN |
| Noa1 | 2610024G14RIK | 5 | Mus musculus nitric oxide associated 1 | 17.67 | 149.96 | METABOLISM/MITO FXN |
| Zfhx2 | ZFHX2 | 14 | Mus musculus zinc finger homeobox 2 (Zfhx2), mRNA. | 17.70 | 177.96 | TRANSCRIPTION/TRANSLATION |
| Tmem179b | TMEM179B | 19 | Mus musculus transmembrane protein 179B (Tmem179b), mRNA. | 17.71 | 177.63 | UNKNOWN FUNCTION |
| Izumo4 | 9030607L17RIK | 10 | Mus musculus IZUMO family member 4 | 17.73 | 187.08 | UNKNOWN FUNCTION |
| Gltp | GLTP | 5 | Mus musculus glycolipid transfer protein (Gltp), mRNA. | 17.73 | 184.30 | INTRACELLULAR TRANSPORT |
| Iba57 | A230051G13RIK | 11 | Mus musculus IBA57, iron-sulfur cluster assembly homolog (S. cerevisiae) | 17.73 | 202.14 | METABOLISM/MITO FXN |
| Hist1h3e | HIST1H3E | 13 | Mus musculus histone cluster 1, H3e (Hist1h3e), mRNA. | 17.73 | 408.48 | CHROMATIN |
| Rps19bp1 | RPS19BP1 | 15 | Mus musculus ribosomal protein S19 binding protein 1 (Rps19bp1), mRNA. | 17.77 | 174.69 | METABOLISM/MITO FXN |
| Ccdc112 | CCDC112 | 18 | PREDICTED: Mus musculus coiled-coil domain containing 112 (Ccdc112), mRNA. | 17.79 | 240.41 | UNKNOWN FUNCTION |
| Rab39 | RAB39 | 9 | Mus musculus RAB39, member RAS oncogene family (Rab39), mRNA. | 17.84 | 319.10 | INTRACELLULAR SIGNAL TRANSDUCTION |
| Macrod1 | MACROD1 | 19 | Mus musculus MACRO domain containing 1 (Macrod1), mRNA. | 17.88 | 237.15 | METABOLISM/MITO FXN |
| Nenf | NENF | 1 | Mus musculus neuron derived neurotrophic factor (Nenf), mRNA. | 17.99 | 219.36 | CELL-CELL COMMUNICATION |
| Wbscr22 | WBSCR22 | 5 | Mus musculus Williams Beuren syndrome chromosome region 22 | 18.03 | 165.32 | CHROMATIN |
| Haghl | HAGHL | 17 | Mus musculus hydroxyacylglutathione hydrolase-like | 18.05 | 176.77 | METABOLISM/MITO FXN |
| Snx6 | SNX6 | 12 | Mus musculus sorting nexin 6 (Snx6), mRNA. | 18.08 | 173.53 | INTRACELLULAR TRANSPORT |
| Hint3 | HINT3 | 10 | Mus musculus histidine triad nucleotide binding protein 3 (Hint3), mRNA. | 18.10 | 195.77 | METABOLISM/MITO FXN |
| Ninl | 4930519N13RIK | 2 | Mus musculus Ninein-like; RIKEN cDNA 4930519N13 gene (4930519N13Rik), mRNA. | 18.12 | 409.00 | METABOLISM/MITO FXN |
| Tmem159 | TMEM159 | 7 | Mus musculus transmembrane protein 159 (Tmem159), mRNA. | 18.12 | 166.63 | UNKNOWN FUNCTION |
| Stx17 | STX17 | 4 | Mus musculus syntaxin 17 | 18.12 | 208.40 | INTRACELLULAR TRANSPORT |
| Dpagt1 | DPAGT1 | 9 | Mus musculus dolichyl-phosphate (UDP-N-acetylglucosamine) acetylglucosaminephosphotransferase 1 (GlcNAc-1-P transferase) (Dpagt1), mRNA. | 18.16 | 153.09 | METABOLISM/MITO FXN |
| D8Ertd738e | D8ERTD738E | 8 | Mus musculus DNA segment, Chr 8, ERATO Doi 738, expressed (D8Ertd738e), mRNA. | 18.24 | 162.94 | UNKNOWN FUNCTION |
| Golm1 | GOLM1 | 13 | Mus musculus golgi membrane protein 1 (Golm1), transcript variant 2, mRNA. | 18.27 | 161.86 | METABOLISM/MITO FXN |
| 4833438C02Rik | 4833438C02RIK | 19 | Mus musculus RIKEN cDNA 4833438C02 gene | 18.35 | 471.80 | UNKNOWN FUNCTION |
| LOC627985 | LOC627985 | 4 | PREDICTED: Mus musculus hypothetical LOC627985 (LOC627985), mRNA. | 18.35 | 151.36 | OTHER |
| Coa6 | 1810063B05RIK | 8 | Mus musculus Cytochrome c oxidase assembly factor 6; RIKEN cDNA 1810063B05 gene (1810063B05Rik), mRNA. | 18.37 | 185.40 | METABOLISM/MITO FXN |
| Exoc3 | EXOC3 | 13 | Mus musculus exocyst complex component 3 (Exoc3), mRNA. | 18.37 | 158.28 | INTRACELLULAR TRANSPORT |
| Zfp326 | ZFP326 | 5 | Mus musculus zinc finger protein 326 (Zfp326), mRNA. | 18.38 | 161.14 | TRANSCRIPTION/TRANSLATION |
| Fubp1 | FUBP1 | 3 | Mus musculus far upstream element (FUSE) binding protein 1 | 18.44 | 158.54 | TRANSCRIPTION/TRANSLATION |
| Mrpl33 | MRPL33 | 5 | Mus musculus mitochondrial ribosomal protein L33 (Mrpl33), nuclear gene encoding mitochondrial protein, mRNA. | 18.45 | 146.89 | METABOLISM/MITO FXN |
| Fkbp9 | FKBP9 | 6 | Mus musculus FK506 binding protein 9 (Fkbp9), mRNA. | 18.47 | 151.10 | METABOLISM/MITO FXN |
| Wdr21 | WDR21 | 12 | Mus musculus WD repeat domain 21 (Wdr21), mRNA. | 18.51 | 157.62 | METABOLISM/MITO FXN |
| Pias4 | PIAS4 | 10 | Mus musculus protein inhibitor of activated STAT 4 (Pias4), mRNA. | 18.66 | 160.91 | TRANSCRIPTION/TRANSLATION |
| Phf2 | PHF2 | 13 | Mus musculus PHD finger protein 2 (Phf2), mRNA. | 18.70 | 150.53 | CHROMATIN |
| Bola2 | BOLA2 | 7 | Mus musculus bolA-like 2 (E. coli) (Bola2), mRNA. | 18.74 | 149.09 | UNKNOWN FUNCTION |
| Ccdc46 | CCDC46 | 11 | Mus musculus coiled-coil domain containing 46 (Ccdc46), transcript variant 2, mRNA. | 18.75 | 183.00 | SYNAPTIC FUNCTION |
| Rnaseh2a | RNASEH2A | 8 | Mus musculus ribonuclease H2, large subunit (Rnaseh2a), mRNA. | 18.76 | 209.67 | METABOLISM/MITO FXN |
| Tmem205 | MGC18837 | 9 | Mus musculus transmembrane protein 205 (Tmem205), mRNA. | 18.76 | 208.09 | UNKNOWN FUNCTION |
| Mfsd9 | MFSD9 | 1 | Mus musculus major facilitator superfamily domain containing 9 (Mfsd9), mRNA. | 18.81 | 409.28 | METABOLISM/MITO FXN |
| ENSMUSG00000068790 | ENSMUSG00000068790 | 14 | Mus musculus predicted gene, ENSMUSG00000068790 (ENSMUSG00000068790), mRNA. | 18.82 | 186.68 | UNKNOWN GENE |
| Prkag1 | PRKAG1 | 15 | Mus musculus protein kinase, AMP-activated, gamma 1 non-catalytic subunit (Prkag1), mRNA. | 18.87 | 149.29 | METABOLISM/MITO FXN |
| Mrpl17 | MRPL17 | 7 | Mus musculus mitochondrial ribosomal protein L17 | 18.91 | 146.78 | METABOLISM/MITO FXN |
| Mthfd2 | MTHFD2 | 6 | Mus musculus methylenetetrahydrofolate dehydrogenase (NAD+ dependent), methenyltetrahydrofolate cyclohydrolase | 18.96 | 204.10 | METABOLISM/MITO FXN |
| Itpripl1 | 1700041B20RIK | 2 | Mus musculus inositol 1,4,5-triphosphate receptor interacting protein-like 1; RIKEN cDNA 1700041B20 gene, transcript variant 1 (1700041B20Rik), mRNA. | 18.97 | 232.66 | UNKNOWN FUNCTION |
| Ddost | DDOST | 4 | Mus musculus dolichyl-di-phosphooligosaccharide-protein glycotransferase (Ddost), mRNA. | 19.01 | 176.94 | METABOLISM/MITO FXN |
| Rfc2 | RFC2 | 5 | Mus musculus replication factor C (activator 1) 2 (Rfc2), mRNA. | 19.15 | 152.57 | DNA REPAIR/REPLICATION |
| Gpaa1 | GPAA1 | 15 | Mus musculus GPI anchor attachment protein 1 (Gpaa1), mRNA. | 19.20 | 164.19 | METABOLISM/MITO FXN |
| Tsen15 | 5730449L18RIK | 1 | Mus musculus Tsen15 tRNA splicing endonuclease 15 homolog; RIKEN cDNA 5730449L18 gene (5730449L18Rik), mRNA. | 19.24 | 151.08 | METABOLISM/MITO FXN |
| Ikbip | 1700023M03RIK | 10 | Mus musculus IKBKB interacting protein | 19.28 | 150.20 | APOPTOSIS |
| Papolg | PAPOLG | 11 | Mus musculus poly(A) polymerase gamma (Papolg), mRNA. | 19.28 | 158.50 | TRANSCRIPTION/TRANSLATION |
| Sord | SORD | 2 | Mus musculus sorbitol dehydrogenase (Sord), mRNA. | 19.30 | 212.83 | METABOLISM/MITO FXN |
| Tmem80 | 5530601I19RIK | 7 | Mus musculus transmembrane protein 80 | 19.30 | 162.18 | UNKNOWN FUNCTION |
| Myg1 | MYG1 | 15 | Mus musculus melanocyte proliferating gene 1 (Myg1), mRNA. | 19.32 | 159.93 | UNKNOWN FUNCTION |
| 2010001F03Rik | 2010001F03RIK | not found |  | 19.36 | 305.82 | UNKNOWN GENE |
| Asphd1 | ASPHD1 | 7 | Mus musculus aspartate beta-hydroxylase domain containing 1 (Asphd1), mRNA. | 19.37 | 153.13 | METABOLISM/MITO FXN |
| Dapk3 | DAPK3 | 10 | Mus musculus death-associated protein kinase 3 (Dapk3), mRNA. | 19.38 | 160.30 | APOPTOSIS |
| Fut10 | FUT10 | 8 | Mus musculus fucosyltransferase 10 (Fut10), transcript variant A, mRNA. | 19.39 | 165.55 | METABOLISM/MITO FXN |
| Rcor1 | RCOR1 | 12 | Mus musculus REST corepressor 1 (Rcor1), mRNA. | 19.47 | 165.34 | TRANSCRIPTION/TRANSLATION |
| Man2c1 | MAN2C1 | 9 | Mus musculus mannosidase, alpha, class 2C, member 1 (Man2c1), mRNA. | 19.53 | 150.25 | METABOLISM/MITO FXN |
| Zfp251 | ZFP251 | 15 | Mus musculus zinc finger protein 251 (Zfp251), mRNA. | 19.60 | 164.54 | TRANSCRIPTION/TRANSLATION |
| Ndufb7 | NDUFB7 | 8 | Mus musculus NADH dehydrogenase (ubiquinone) 1 beta subcomplex, 7 (Ndufb7), nuclear gene encoding mitochondrial protein, mRNA. | 19.61 | 156.71 | METABOLISM/MITO FXN |
| Hes6 | HES6 | 1 | Mus musculus hairy and enhancer of split 6 (Drosophila) (Hes6), mRNA. | 19.65 | 157.97 | TRANSCRIPTION/TRANSLATION |
| LOC667582 | LOC667582 | 13 | PREDICTED: Mus musculus similar to EAP30 subunit of ELL complex (LOC667582), misc RNA. | 19.71 | 149.72 | OTHER |
| Cc2d1a | CC2D1A | 8 | Mus musculus coiled-coil and C2 domain containing 1A (Cc2d1a), mRNA. | 19.90 | 151.10 | SYNAPTIC FUNCTION |
| Shf | SHF | 2 | Mus musculus Src homology 2 domain containing F (Shf), mRNA. | 19.95 | 282.19 | APOPTOSIS |
| 8430410A17Rik | 8430410A17RIK | 6 | Mus musculus RIKEN cDNA 8430410A17 gene (8430410A17Rik), mRNA. | 20.04 | 222.46 | UNKNOWN FUNCTION |
| Limd1 | LIMD1 | 9 | Mus musculus LIM domains containing 1 (Limd1), mRNA. | 20.04 | 198.59 | TRANSCRIPTION/TRANSLATION |
| LOC381947 | LOC381947 |  |  | 20.04 | 153.74 | OTHER |
| Msl3l2 | MSL3L2 | 10 | Mus musculus male-specific lethal 3-like 2 (Drosophila) (Msl3l2), non-coding RNA. | 20.04 | 186.51 | UNKNOWN FUNCTION |
| Rab33b | RAB33B | 3 | Mus musculus RAB33B, member of RAS oncogene family (Rab33b), mRNA. | 20.04 | 150.83 | INTRACELLULAR SIGNAL TRANSDUCTION |
| Unc119b | UNC119B | 5 | Mus musculus unc-119 homolog B (C. elegans) (Unc119b), mRNA. | 20.07 | 174.22 | CILIA COMPONENT |
| Txnip | TXNIP | 3 | Mus musculus thioredoxin interacting protein (Txnip), transcript variant 1, mRNA. | 20.12 | 629.26 | INTRACELLULAR SIGNAL TRANSDUCTION |
| Wdr8 | WDR8 | 4 | Mus musculus WD repeat domain 8 | 20.22 | 177.16 | UNKNOWN FUNCTION |
| Setmar | SETMAR | 6 | PREDICTED: Mus musculus SET domain and mariner transposase fusion gene (Setmar), mRNA. | 20.26 | 188.77 | CHROMATIN |
| Sri | SRI | 5 | Mus musculus sorcin (Sri), transcript variant 1, mRNA. | 20.27 | 200.78 | INTRACELLULAR SIGNAL TRANSDUCTION |
| Lsm8 | LSM8 | 6 | Mus musculus LSM8 homolog, U6 small nuclear RNA associated (S. cerevisiae) (Lsm8), mRNA. | 20.29 | 149.16 | TRANSCRIPTION/TRANSLATION |
| Arfgap3 | ARFGAP3 | 15 | Mus musculus ADP-ribosylation factor GTPase activating protein 3 (Arfgap3), mRNA. | 20.39 | 190.74 | INTRACELLULAR TRANSPORT |
| Gm12942 | LOC100046844 | 4 | PREDICTED: Mus musculus similar to transmembrane protein 35 (LOC100046844), mRNA. | 20.52 | 424.71 | UNKNOWN FUNCTION |
| LOC385822 | LOC385822 |  |  | 20.52 | 187.93 | OTHER |
| Dennd2a | DENND2A |  | Mus musculus DENN/MADD domain containing 2A (Dennd2a), mRNA. | 20.57 | 151.09 | INTRACELLULAR TRANSPORT |
| Irf1 | IRF1 | 11 | Mus musculus interferon regulatory factor 1 (Irf1), mRNA. | 20.63 | 245.74 | TRANSCRIPTION/TRANSLATION |
| Aldh18a1 | ALDH18A1 | 19 | Mus musculus aldehyde dehydrogenase 18 family, member A1 (Aldh18a1), nuclear gene encoding mitochondrial protein, transcript variant 1, mRNA. | 20.66 | 164.54 | METABOLISM/MITO FXN |
| Myl6 | MYL6 | 10 | Mus musculus myosin, light polypeptide 6, alkali, smooth muscle and non-muscle | 20.67 | 171.67 | INTRACELLULAR SIGNAL TRANSDUCTION |
| Brd2 | BRD2 | 17 | Mus musculus bromodomain containing 2 (Brd2), transcript variant 2, mRNA. | 20.69 | 164.79 | TRANSCRIPTION/TRANSLATION |
| Hyal2 | HYAL2 | 9 | Mus musculus hyaluronoglucosaminidase 2 (Hyal2), mRNA. | 20.72 | 195.26 | METABOLISM/MITO FXN |
| Map4k4 | 9430080K19RIK | 1 | Mus musculus mitogen-activated protein kinase kinase kinase kinase 4 | 20.81 | 149.80 | INTRACELLULAR SIGNAL TRANSDUCTION |
| Rpl19 | RPL19 | 11 | Mus musculus ribosomal protein L19 (Rpl19), mRNA. | 20.81 | 152.71 | METABOLISM/MITO FXN |
| Rnaseh2c | RNASEH2C | 19 | Mus musculus ribonuclease H2, subunit C (Rnaseh2c), mRNA. | 20.84 | 194.68 | CELL CYCLE |
| Nme2 | NME2 | 11 | Mus musculus non-metastatic cells 2, protein (NM23B) expressed in (Nme2), transcript variant 1, mRNA. | 20.84 | 173.29 | METABOLISM/MITO FXN |
| H2afx | H2AFX | 9 | Mus musculus H2A histone family, member X (H2afx), mRNA. | 20.95 | 155.69 | CHROMATIN |
| Tpm4 | TPM4 | 8 | Mus musculus tropomyosin 4 (Tpm4), mRNA. | 21.05 | 184.38 | CYTOSKELETAL/NUCLEAR ENVELOPE |
| Rem2 | REM2 | 14 | Mus musculus rad and gem related GTP binding protein 2 (Rem2), mRNA. | 21.06 | 168.08 | INTRACELLULAR SIGNAL TRANSDUCTION |
| Tmem128 | 2810021O14RIK | 5 | Mus musculus transmembrane protein 128 | 21.13 | 171.74 | UNKNOWN FUNCTION |
| Siva1 | SIVA1 | 12 | Mus musculus SIVA1, apoptosis-inducing factor (Siva1), mRNA. | 21.14 | 190.95 | APOPTOSIS |
| LOC100048696 | LOC100048696 |  | PREDICTED: Mus musculus hypothetical protein LOC100048696 (LOC100048696), mRNA. | 21.17 | 281.05 | OTHER |
| AI846148 | AI846148 | 19 | Mus musculus expressed sequence AI846148 (AI846148), mRNA. XM_896219 XM_896224 XM_912777 XM_922495 XM_922500 XM_922504 XM_922508 XM_922513 XM_922518 | 21.17 | 164.54 | UNKNOWN FUNCTION |
| Alkbh7 | ALKBH7 | 17 | Mus musculus alkB, alkylation repair homolog 7 (E. coli) (Alkbh7), mRNA. | 21.22 | 159.08 | METABOLISM/MITO FXN |
| Vangl2 | VANGL2 | 1 | Mus musculus vang-like 2 (van gogh, Drosophila) (Vangl2), mRNA. | 21.34 | 268.98 | CELL-CELL COMMUNICATION |
| Zfp771 | ZFP771 | 7 | Mus musculus zinc finger protein 771 (Zfp771), mRNA. | 21.42 | 188.25 | TRANSCRIPTION/TRANSLATION |
| Actr1b | ACTR1B | 1 | Mus musculus ARP1 actin-related protein 1B, centractin beta | 21.46 | 140.41 | INTRACELLULAR TRANSPORT |
| A430106B04Rik | A430106B04RIK | not found |  | 21.65 | 175.88 | UNKNOWN GENE |
| Pcif | 4930445K14RIK | 2 | Mus musculus PDX1 C-terminal inhibiting factor 1; RIKEN cDNA 4930445K14RIK, mRNA. | 21.65 | 155.85 | METABOLISM/MITO FXN |
| Hist1h3h | HIST1H3H | 13 | Mus musculus histone cluster 1, H3h (Hist1h3h), mRNA. | 21.67 | 251.31 | CHROMATIN |
| Acd | ACD | 8 | Mus musculus adrenocortical dysplasia (Acd), mRNA. | 21.67 | 171.02 | METABOLISM/MITO FXN |
| Cggbp1 | CGGBP1 | 16 | Mus musculus CGG triplet repeat binding protein 1 (Cggbp1), mRNA. | 21.70 | 150.67 | TRANSCRIPTION/TRANSLATION |
| Ino80b | INO80B | 6 | Mus musculus INO80 complex subunit B (Ino80b), mRNA. | 21.71 | 171.93 | CHROMATIN |
| Mrps28 | MRPS28 | 3 | Mus musculus mitochondrial ribosomal protein S28 (Mrps28), nuclear gene encoding mitochondrial protein, mRNA. | 21.74 | 185.64 | METABOLISM/MITO FXN |
| Dnase2a | DNASE2A | 8 | Mus musculus deoxyribonuclease II alpha (Dnase2a), mRNA. | 21.80 | 593.41 | METABOLISM/MITO FXN |
| Rps6ka1 | RPS6KA1 | 4 | Mus musculus ribosomal protein S6 kinase polypeptide 1 (Rps6ka1), mRNA. | 21.85 | 192.21 | INTRACELLULAR SIGNAL TRANSDUCTION |
| Rfxap | RFXAP | 3 | Mus musculus regulatory factor X-associated protein (Rfxap), mRNA. | 21.87 | 158.49 | TRANSCRIPTION/TRANSLATION |
| 4933431K14Rik | 4933431K14RIK | 9 | PREDICTED: Mus musculus RIKEN cDNA 4933431K14 gene (4933431K14Rik), mRNA. | 21.93 | 208.85 | UNKNOWN FUNCTION |
| Cib2 | CIB2 | 9 | Mus musculus calcium and integrin binding family member 2 (Cib2), mRNA. | 21.95 | 255.95 | INTRACELLULAR SIGNAL TRANSDUCTION |
| Dpp3 | DPP3 | 19 | Mus musculus dipeptidylpeptidase 3 (Dpp3), mRNA. | 22.08 | 160.68 | METABOLISM/MITO FXN |
| Psmb6 | LOC100048803 | 11 | Mus musculus proteasome (prosome, macropain) subunit, beta type 6 | 22.20 | 157.68 | METABOLISM/MITO FXN |
| Actl6b | ACTL6B | 5 | Mus musculus actin-like 6B (Actl6b), mRNA. | 22.20 | 183.31 | CHROMATIN |
| Haus5 | 2310022K01RIK | 7 | Mus musculus HAUS augmin-like complex, subunit 5 | 22.21 | 191.84 | CELL CYCLE |
| Ccdc84 | CCDC84 | 9 | Mus musculus coiled-coil domain containing 84 (Ccdc84), mRNA. | 22.22 | 169.24 | UNKNOWN FUNCTION |
| Psmb1 | PSMB1 | 17 | Mus musculus proteasome (prosome, macropain) subunit, beta type 1 (Psmb1), mRNA. | 22.23 | 150.44 | METABOLISM/MITO FXN |
| scl000408.1_6 | SCL000408.1_6 |  |  | 22.35 | 212.48 | UNKNOWN GENE |
| Hus1 | HUS1 | 11 | Mus musculus Hus1 homolog (S. pombe) (Hus1), mRNA. | 22.38 | 169.35 | CELL CYCLE |
| Snrpa | SNRPA | 7 | Mus musculus small nuclear ribonucleoprotein polypeptide A (Snrpa), transcript variant 2, mRNA. | 22.38 | 165.25 | TRANSCRIPTION/TRANSLATION |
| 2210016F16Rik | 2210016F16RIK | 13 | PREDICTED: Mus musculus RIKEN cDNA 2210016F16 gene (2210016F16Rik), mRNA. | 22.57 | 250.99 | UNKNOWN FUNCTION |
| Mpnd | MPND | 17 | Mus musculus MPN domain containing (Mpnd), mRNA. | 22.58 | 168.47 | METABOLISM/MITO FXN |
| Tmem14c | TMEM14C | 13 | Mus musculus transmembrane protein 14C (Tmem14c), mRNA. | 22.58 | 167.07 | UNKNOWN FUNCTION |
| Zc3hc1 | ZC3HC1 | 6 | Mus musculus zinc finger, C3HC type 1 (Zc3hc1), mRNA. | 22.58 | 175.52 | CELL CYCLE |
| Tmed4 | TMED4 | 11 | Mus musculus transmembrane emp24 protein transport domain containing 4 (Tmed4), mRNA. | 22.62 | 168.54 | METABOLISM/MITO FXN |
| Lin7b | LIN7B | 7 | Mus musculus lin-7 homolog B (C. elegans) (Lin7b), mRNA. | 22.66 | 218.48 | CELL ADHESION |
| Eif2ak2 | EIF2AK2 | 17 | Mus musculus eukaryotic translation initiation factor 2-alpha kinase 2 (Eif2ak2), mRNA. | 22.70 | 287.03 | TRANSCRIPTION/TRANSLATION |
| Bcl7c | BCL7C | 7 | Mus musculus B cell CLL/lymphoma 7C | 22.74 | 153.68 | APOPTOSIS |
| EG666609 | EG666609 | 17 | PREDICTED: Mus musculus predicted gene, EG666609 (EG666609), mRNA. | 22.77 | 150.68 | TRANSCRIPTION/TRANSLATION |
| Tmem160 | TMEM160 | 7 | Mus musculus transmembrane protein 160 (Tmem160), mRNA. | 22.79 | 179.35 | UNKNOWN FUNCTION |
| Ethe1 | ETHE1 | 7 | Mus musculus ethylmalonic encephalopathy 1 (Ethe1), mRNA. | 22.80 | 189.78 | METABOLISM/MITO FXN |
| Nadk | NADK | 4 | Mus musculus NAD kinase (Nadk), mRNA. | 22.82 | 177.92 | METABOLISM/MITO FXN |
| Cgrrf1 | 1110038G02RIK | 14 | Mus musculus cell growth regulator with ring finger domain 1 | 22.84 | 162.89 | CELL CYCLE |
| Xkr8 | XKR8 | 4 | Mus musculus X Kell blood group precursor related family member 8 homolog (Xkr8), mRNA. | 22.97 | 191.06 | UNKNOWN FUNCTION |
| Nt5c3l | NT5C3L | 11 | Mus musculus 5'-nucleotidase, cytosolic III-like (Nt5c3l), mRNA. | 23.08 | 198.33 | METABOLISM/MITO FXN |
| Rab42 | 9530096D07RIK | 4 | Mus musculus Rab42, member RAS oncogene family; RIKEN cDNA 9530096D07 gene (9530096D07Rik), mRNA. | 23.13 | 311.71 | INTRACELLULAR SIGNAL TRANSDUCTION |
| Smarcb1 | SMARCB1 | 10 | Mus musculus SWI/SNF related, matrix associated, actin dependent regulator of chromatin, subfamily b, member 1 (Smarcb1), mRNA. | 23.19 | 197.70 | CHROMATIN |
| D930048N14Rik | D930048N14RIK | 11 | PREDICTED: Mus musculus RIKEN cDNA D930048N14 gene (D930048N14Rik), misc RNA. | 23.40 | 259.47 | UNKNOWN FUNCTION |
| EG216185 | EG216185 | 10 | PREDICTED: Mus musculus predicted gene, EG216185 (EG216185), mRNA. | 23.47 | 154.18 | UNKNOWN FUNCTION |
| Vps37d | VPS37D | 5 | Mus musculus vacuolar protein sorting 37D (yeast) (Vps37d), mRNA. | 23.62 | 171.79 | INTRACELLULAR TRANSPORT |
| Pex26 | PEX26 | 6 | Mus musculus peroxisome biogenesis factor 26 (Pex26), mRNA. | 23.79 | 179.79 | METABOLISM/MITO FXN |
| Mmgt2 | BC025076 | 11 | Mus musculus membrane magnesium transporter2 | 23.93 | 189.27 | METABOLISM/MITO FXN |
| Slc29a3 | SLC29A3 | 10 | Mus musculus solute carrier family 29 (nucleoside transporters), member 3 (Slc29a3), mRNA. | 23.93 | 196.76 | METABOLISM/MITO FXN |
| Cdk4 | CDK4 | 10 | Mus musculus cyclin-dependent kinase 4 (Cdk4), mRNA. | 23.94 | 183.94 | CELL CYCLE |
| Rhbdd2 | RHBDL7 | 5 | Mus musculus rhomboid domain containing 2 | 23.97 | 165.07 | UNKNOWN FUNCTION |
| Hyi | HYI | 4 | Mus musculus hydroxypyruvate isomerase homolog (E. coli) (Hyi), mRNA. | 24.01 | 170.31 | METABOLISM/MITO FXN |
| Ppapdc2 | PPAPDC2 | 19 | Mus musculus phosphatidic acid phosphatase type 2 domain containing 2 (Ppapdc2), mRNA. | 24.07 | 161.00 | METABOLISM/MITO FXN |
| Arpc3 | ARPC3 | 5 | Mus musculus actin related protein 2/3 complex, subunit 3 (Arpc3), mRNA. | 24.15 | 158.45 | CYTOSKELETAL/NUCLEAR ENVELOPE |
| Ints10 | INTS10 | 8 | Mus musculus integrator complex subunit 10 (Ints10), mRNA. | 24.16 | 155.76 | TRANSCRIPTION/TRANSLATION |
| 2510022D24Rik | 2510022D24RIK |  | PREDICTED: Mus musculus RIKEN cDNA 2510022D24 gene (2510022D24Rik), mRNA. | 24.18 | 160.24 | UNKNOWN FUNCTION |
| LOC381256 | LOC381256 |  |  | 24.18 | 264.54 | OTHER |
| Polr2j | POLR2J | 5 | Mus musculus polymerase (RNA) II (DNA directed) polypeptide J (Polr2j), mRNA. | 24.21 | 153.28 | TRANSCRIPTION/TRANSLATION |
| 2310057K05Rik | 2310057K05RIK | not found |  | 24.21 | 178.94 | UNKNOWN GENE |
| Hes5 | HES5 | 4 | Mus musculus hairy and enhancer of split 5 (Drosophila) (Hes5), mRNA. | 24.29 | 216.72 | TRANSCRIPTION/TRANSLATION |
| Mcm9 | BC062185 | 10 | Mus musculus minichromosome maintenance complex component 9 | 24.40 | 201.72 | CELL CYCLE |
| Gm1673 | GM1673 | 5 | Mus musculus gene model 1673, (NCBI) (Gm1673), mRNA. XM_922421 | 24.41 | 161.62 | UNKNOWN FUNCTION |
| Lamtor2 | MAPBPIP-PENDING | 3 | Mus musculus late endosomal/lysosomal adaptor, MAPK and MTOR activator 2 | 24.43 | 157.32 | METABOLISM/MITO FXN |
| Tmem176b | TMEM176B | 6 | Mus musculus transmembrane protein 176B (Tmem176b), mRNA. | 24.52 | 195.94 | UNKNOWN FUNCTION |
| Cd63 | CD63 | 10 | Mus musculus CD63 antigen | 24.67 | 179.89 | INTRACELLULAR TRANSPORT |
| 2810032G03Rik | 2810032G03RIK | 12 |  | 24.74 | 191.39 | UNKNOWN FUNCTION |
| 2010004M13Rik | 2010004M13RIK | 7 |  | 24.77 | 163.14 | UNKNOWN FUNCTION |
| Hint2 | HINT2 | 4 | Mus musculus histidine triad nucleotide binding protein 2 (Hint2), mRNA. | 24.79 | 164.73 | METABOLISM/MITO FXN |
| Cflar | 2310024N18RIK | 1 | CASP8 and FADD-like apoptosis regulator | 24.93 | 162.64 | APOPTOSIS |
| Renbp | RENBP | X | Mus musculus renin binding protein (Renbp), mRNA. | 24.99 | 180.81 | METABOLISM/MITO FXN |
| LOC100048589 | LOC100048589 |  | PREDICTED: Mus musculus similar to CDNA sequence BC052040, transcript variant 1 (LOC100048589), mRNA. | 25.03 | 166.85 | OTHER |
| Ccdc167 | 1110021J02RIK | 17 | Mus musculus coiled-coil domain containing 167 | 25.03 | 218.47 | UNKNOWN FUNCTION |
| 9530077C05Rik | 9530077C05RIK | 9 | Mus musculus RIKEN cDNA 9530077C05 gene (9530077C05Rik), mRNA. | 25.04 | 185.20 | UNKNOWN FUNCTION |
| Cbwd1 | CBWD1 | 19 | Mus musculus COBW domain containing 1 (Cbwd1), mRNA. | 25.06 | 172.51 | UNKNOWN FUNCTION |
| Fchsd2 | FCHSD2 | 7 | Mus musculus FCH and double SH3 domains 2 (Fchsd2), mRNA. | 25.12 | 157.30 | UNKNOWN FUNCTION |
| Mrpl27 | MRPL27 | 11 | Mus musculus mitochondrial ribosomal protein L27 (Mrpl27), nuclear gene encoding mitochondrial protein, mRNA. | 25.12 | 155.69 | METABOLISM/MITO FXN |
| EG667190 | EG667190 | X | PREDICTED: Mus musculus predicted gene, EG667190 (EG667190), misc RNA. | 25.23 | 181.01 | OTHER |
| Gtf2h4 | GTF2H4 | 17 | Mus musculus general transcription factor II H, polypeptide 4 (Gtf2h4), mRNA. | 25.23 | 166.53 | TRANSCRIPTION/TRANSLATION |
| Psmb6 | PSMB6 | 11 | Mus musculus proteasome (prosome, macropain) subunit, beta type 6 (Psmb6), mRNA. | 25.32 | 153.57 | METABOLISM/MITO FXN |
| Rbfa | 1110032A13RIK | 18 | Mus musculus ribosome binding factor A; RIKEN cDNA 1110032A13 gene (1110032A13Rik), mRNA. | 25.38 | 163.45 | METABOLISM/MITO FXN |
| Rbfa | 1110032A13RIK | 18 | Mus musculus ribosome binding factor A; RIKEN cDNA 1110032A13 gene (1110032A13Rik), mRNA. | 25.38 | 163.45 | METABOLISM/MITO FXN |
| Copg | COPG | 6 | Mus musculus coatomer protein complex, subunit gamma (Copg), transcript variant 2, mRNA. | 25.43 | 136.56 | INTRACELLULAR TRANSPORT |
| Srrp | SRRP | 4 | Mus musculus serine-arginine repressor protein (Srrp), mRNA. | 25.49 | 184.87 | TRANSCRIPTION/TRANSLATION |
| C230098O21Rik | C230098O21RIK | 8 |  | 25.79 | 203.04 | UNKNOWN FUNCTION |
| Mphosph6 | MPHOSPH6 | 8 | Mus musculus M phase phosphoprotein 6 (Mphosph6), mRNA. | 25.83 | 157.94 | METABOLISM/MITO FXN |
| Rabl4 | RABL4 | 15 | Mus musculus RAB, member of RAS oncogene family-like 4 (Rabl4), mRNA. | 25.92 | 158.42 | CILIA COMPONENT |
| Snrpf | SNRPF | 10 | PREDICTED: Mus musculus small nuclear ribonucleoprotein polypeptide F (Snrpf), mRNA. | 26.03 | 156.57 | TRANSCRIPTION/TRANSLATION |
| Zfp41 | ZFP41 | 15 | Mus musculus zinc finger protein 414 (Zfp414), mRNA. | 26.11 | 164.10 | TRANSCRIPTION/TRANSLATION |
| Hist1h2ai | HIST1H2AI | 13 | Mus musculus histone cluster 1, H2ai (Hist1h2ai), mRNA. | 26.26 | 233.80 | CHROMATIN |
| LOC100041500 | LOC100041500 | 10 | PREDICTED: Mus musculus similar to LSM7 homolog, U6 small nuclear RNA associated (LOC100041500), mRNA. | 26.34 | 155.71 | UNKNOWN FUNCTION |
| LOC100046039 | LOC100046039 |  | PREDICTED: Mus musculus similar to histone deacetylase HD1 (LOC100046039), mRNA. | 26.39 | 195.92 | OTHER |
| Npm3 | NPM3 | 19 | Mus musculus nucleoplasmin 3 (Npm3), mRNA. | 26.40 | 191.76 | CHROMATIN |
| Mmp11 | MMP11 | 10 | Mus musculus matrix metallopeptidase 11 (Mmp11), mRNA. | 26.46 | -1069.18 | METABOLISM/MITO FXN |
| Banf1 | BANF1 | 19 | Mus musculus barrier to autointegration factor 1 (Banf1), transcript variant 1, mRNA. | 26.52 | 163.82 | CELL CYCLE |
| N6amt2 | N6AMT2 | 14 | Mus musculus N-6 adenine-specific DNA methyltransferase 2 (putative) (N6amt2), mRNA. | 26.60 | 196.73 | CHROMATIN |
| Gm8387 | LOC677180 | 16 | PREDICTED: Mus musculus similar to translocase of inner mitochondrial membrane 17 homolog B (yeast) (LOC677180), misc RNA. | 26.66 | 175.17 | OTHER |
| LOC331595 | LOC331595 |  |  | 26.69 | -238.27 | OTHER |
| Nradd | NRADD | 9 | Mus musculus neurotrophin receptor associated death domain (Nradd), mRNA. | 26.70 | 423.93 | APOPTOSIS |
| Hint1 | HINT1 | 11 | Mus musculus histidine triad nucleotide binding protein 1 (Hint1), mRNA. | 26.74 | 155.06 | METABOLISM/MITO FXN |
| Tcfcp2l1 | TCFCP2L1 | 1 | Mus musculus transcription factor CP2-like 1 (Tcfcp2l1), mRNA. | 26.77 | -502.47 | TRANSCRIPTION/TRANSLATION |
| Cib1 | CIB1 | 7 | Mus musculus calcium and integrin binding 1 (calmyrin) (Cib1), mRNA. | 26.99 | 165.41 | APOPTOSIS |
| Nudt1 | NUDT1 | 5 | Mus musculus nudix (nucleoside diphosphate linked moiety X)-type motif 1 (Nudt1), mRNA. | 27.00 | 200.15 | DNA REPAIR/REPLICATION |
| Setd6 | SETD6 | 8 | Mus musculus SET domain containing 6 (Setd6), mRNA. | 27.16 | 171.42 | CHROMATIN |
| 2810428I15Rik | 2810428I15RIK | 8 |  | 27.17 | 160.36 | UNKNOWN FUNCTION |
| Fam96b | 1110019N10RIK | 8 | Mus musculus RIKEN cDNA 1110019N10 gene;family with sequence similarity 96, member B | 27.18 | 168.19 | CELL CYCLE |
| Triap1 | TRIAP1 | 5 | Mus musculus TP53 regulated inhibitor of apoptosis 1 (Triap1), mRNA. | 27.19 | 160.91 | APOPTOSIS |
| Msh6 | MSH6 | 17 | Mus musculus mutS homolog 6 (E. coli) (Msh6), mRNA. | 27.25 | 158.72 | DNA REPAIR/REPLICATION |
| Gusb | GUS-S | 5 | Mus musculus glucuronidase, beta | 27.28 | 207.67 | METABOLISM/MITO FXN |
| Tbcb | TBCB | 7 | Mus musculus tubulin folding cofactor B (Tbcb), mRNA. | 27.48 | 156.46 | METABOLISM/MITO FXN |
| Atat1 | 2610110G12RIK | 17 | Mus musculus alpha tubulin acetyltransferase 1 | 27.49 | 234.12 | METABOLISM/MITO FXN |
| Dcaf15 | BC057552 | 8 | Mus musculus DDB1 and CUL4 associated factor 15 | 27.51 | 166.48 | UNKNOWN FUNCTION |
| Ints7 | INTS7 | 1 | Mus musculus integrator complex subunit 7 (Ints7), mRNA. | 27.51 | 162.20 | TRANSCRIPTION/TRANSLATION |
| Gnaq | 6230401I02RIK | 19 | Mus musculus guanine nucleotide binding protein, alpha q polypeptide | 27.58 | 158.11 | INTRACELLULAR SIGNAL TRANSDUCTION |
| Fibp | FIBP | 19 | Mus musculus fibroblast growth factor (acidic) intracellular binding protein | 27.68 | 163.42 | CELL-CELL COMMUNICATION |
| Skap2 | SKAP2 | 6 | Mus musculus src family associated phosphoprotein 2 (Skap2), mRNA. | 27.79 | 225.60 | INTRACELLULAR SIGNAL TRANSDUCTION |
| Pard6g | PARD6G | 18 | Mus musculus par-6 partitioning defective 6 homolog gamma (C. elegans) (Pard6g), mRNA. | 27.82 | 170.87 | CELL ADHESION |
| Wtip | WTIP | 7 | Mus musculus WT1-interacting protein (Wtip), mRNA. | 27.91 | 161.11 | TRANSCRIPTION/TRANSLATION |
| LOC100043919 | LOC100043919 | Un\|NT_166434.1 | PREDICTED: Mus musculus hypothetical protein LOC100043919 (LOC100043919), mRNA. | 27.92 | 286.59 | OTHER |
| Taf9b | TAF9B | X | Mus musculus TAF9B RNA polymerase II, TATA box binding protein (TBP)-associated factor (Taf9b), mRNA. | 27.93 | 178.67 | TRANSCRIPTION/TRANSLATION |
| 1500004F05Rik | 1500004F05RIK | 8 | PREDICTED: Mus musculus RIKEN cDNA 1500004F05 gene (1500004F05Rik), mRNA. | 28.02 | 160.34 | UNKNOWN FUNCTION |
| Phb2 | PHB2 | 6 | Mus musculus prohibitin 2 (Phb2), mRNA. | 28.15 | 161.52 | TRANSCRIPTION/TRANSLATION |
| Vgll4 | VGLL4 | 6 | Mus musculus vestigial like 4 (Drosophila) (Vgll4), mRNA. | 28.25 | 220.49 | TRANSCRIPTION/TRANSLATION |
| Azi1 | AZI1 | 11 | Mus musculus 5-azacytidine induced gene 1 (Azi1), mRNA. | 28.27 | 175.85 | CELL CYCLE |
| Elof1 | ELOF1 | 9 | Mus musculus elongation factor 1 homolog (ELF1, S. cerevisiae) (Elof1), mRNA. | 28.27 | 161.27 | TRANSCRIPTION/TRANSLATION |
| Akr7a5 | AKR7A5 | 4 | Mus musculus aldo-keto reductase family 7, member A5 (aflatoxin aldehyde reductase) (Akr7a5), mRNA. | 28.28 | 155.15 | METABOLISM/MITO FXN |
| Tmem185b | TMEM185B | 1 | Mus musculus transmembrane protein 185B (Tmem185b), mRNA. | 28.28 | 165.50 | UNKNOWN FUNCTION |
| Fbxl6 | FBXL6 | 15 | Mus musculus F-box and leucine-rich repeat protein 6 (Fbxl6), mRNA. | 28.39 | 166.46 | METABOLISM/MITO FXN |
| Tnfrsf12a | TNFRSF12A | 17 | Mus musculus tumor necrosis factor receptor superfamily, member 12a (Tnfrsf12a), mRNA. | 28.51 | 363.34 | APOPTOSIS |
| Blvrb | BLVRB | 7 | Mus musculus biliverdin reductase B (flavin reductase (NADPH)) (Blvrb), mRNA. | 28.62 | 451.19 | METABOLISM/MITO FXN |
| Ttf2 | TTF2 | 3 | Mus musculus transcription termination factor, RNA polymerase II (Ttf2), mRNA. | 28.65 | 272.92 | TRANSCRIPTION/TRANSLATION |
| Ano10 | ANO10 | 9 | Mus musculus anoctamin 10 (Ano10), mRNA. | 28.67 | 202.72 | TRANSMEMBRANE TRANSPORT |
| Eif4e3 | EIF4E3 | 6 | Mus musculus eukaryotic translation initiation factor 4E member 3 (Eif4e3), mRNA. | 28.71 | 221.72 | TRANSCRIPTION/TRANSLATION |
| Tmem126a | TMEM126A | 7 | Mus musculus transmembrane protein 126A (Tmem126a), mRNA. | 28.75 | 159.65 | RETINAL DISEASE GENE |
| Alkbh6 | ALKBH6 | 7 | Mus musculus alkB, alkylation repair homolog 6 (E. coli) (Alkbh6), mRNA. | 28.83 | 166.95 | METABOLISM/MITO FXN |
| Spsb2 | SPSB2 | 6 | Mus musculus splA/ryanodine receptor domain and SOCS box containing 2 (Spsb2), mRNA. | 29.12 | 396.89 | CELL-CELL COMMUNICATION |
| Smarca1 | SMARCA1 | X | Mus musculus SWI/SNF related, matrix associated, actin dependent regulator of chromatin, subfamily a, member 1 (Smarca1), mRNA. | 29.29 | 186.72 | CHROMATIN |
| Nsmce1 | NSMCE1 | 7 | Mus musculus non-SMC element 1 homolog (S. cerevisiae) (Nsmce1), mRNA. | 29.45 | 222.14 | DNA REPAIR/REPLICATION |
| Txnl4 | TXNL4 | 18 | Mus musculus thioredoxin-like 4A | 29.45 | 170.39 | TRANSCRIPTION/TRANSLATION |
| Pgbd1 | PGBD1 | 13 | PREDICTED: Mus musculus piggyBac transposable element derived 1 (Pgbd1), misc RNA. | 29.75 | 283.90 | TRANSCRIPTION/TRANSLATION |
| Nudt22 | NUDT22 | 19 | Mus musculus nudix (nucleoside diphosphate linked moiety X)-type motif 22 (Nudt22), mRNA. | 29.76 | 166.29 | UNKNOWN FUNCTION |
| Sfrs3 | SFRS3 | 17 | Mus musculus splicing factor, arginine/serine-rich 3 (SRp20) (Sfrs3), mRNA. | 29.80 | 165.67 | TRANSCRIPTION/TRANSLATION |
| Brd9 | BRD9 | 13 | Mus musculus bromodomain containing 9 (Brd9), mRNA. | 29.86 | 161.32 | TRANSCRIPTION/TRANSLATION |
| Dhps | DHPS | 8 | Mus musculus deoxyhypusine synthase (Dhps), mRNA. | 29.86 | 163.81 | TRANSCRIPTION/TRANSLATION |
| Med11 | MED11 | 11 | Mus musculus mediator of RNA polymerase II transcription, subunit 11 homolog (S. cerevisiae) (Med11), mRNA. | 29.90 | 195.84 | TRANSCRIPTION/TRANSLATION |
| Lmnb2 | LMNB2 | 10 | Mus musculus lamin B2 (Lmnb2), mRNA. | 29.98 | 181.27 | CYTOSKELETAL/NUCLEAR ENVELOPE |
| Prmt1 | HRMT1L2 | 7 | Mus musculus protein arginine N-methyltransferase 1 | 30.13 | 171.13 | CHROMATIN |
| Accs | ACCS | 2 | Mus musculus 1-aminocyclopropane-1-carboxylate synthase homolog (Arabidopsis)(non-functional) (Accs), mRNA. | 30.23 | 230.53 | METABOLISM/MITO FXN |
| Dnajc15 | DNAJC15 | 14 | Mus musculus DnaJ (Hsp40) homolog, subfamily C, member 15 (Dnajc15), mRNA. | 30.33 | 162.61 | METABOLISM/MITO FXN |
| Ppp1r26 | GM347 | 2 | Mus musculus gene model 347, (NCBI); protein phosphatase 1 regulatory unit 26 | 30.41 | 161.41 | METABOLISM/MITO FXN |
| Pold1 | POLD1 | 7 | Mus musculus polymerase (DNA directed), delta 1, catalytic subunit (Pold1), mRNA. | 30.44 | 177.13 | DNA REPAIR/REPLICATION |
| Rnpep | RNPEP | 1 | Mus musculus arginyl aminopeptidase (aminopeptidase B) | 30.66 | 194.60 | METABOLISM/MITO FXN |
| Pih1d1 | PIH1D1 | 7 | Mus musculus PIH1 domain containing 1 (Pih1d1), mRNA. | 30.92 | 166.85 | METABOLISM/MITO FXN |
| Gm6722 | LOC268569 | 12 | Mus musculus predicted gene 6722, H2A histone family, member Z pseudogene | 30.94 | 159.45 | OTHER |
| H2afz | H2AFZ | 3 | Mus musculus H2A histone family, member Z (H2afz), mRNA. | 30.94 | 160.28 | CHROMATIN |
| Sfrs7 | SFRS7 | 17 | Mus musculus splicing factor, arginine/serine-rich 7 (Sfrs7), mRNA. | 30.94 | 161.34 | TRANSCRIPTION/TRANSLATION |
| Znrf2 | ZNRF2 | 6 | Mus musculus zinc and ring finger 2 (Znrf2), mRNA. | 30.94 | 177.03 | SYNAPTIC FUNCTION |
| Hsd17b4 | HSD17B4 | 18 | Mus musculus hydroxysteroid (17-beta) dehydrogenase 4 (Hsd17b4), mRNA. | 30.98 | 160.57 | METABOLISM/MITO FXN |
| Bex4 | BEX4 | X | Mus musculus brain expressed gene 4 (Bex4), mRNA. | 31.12 | 252.39 | TRANSCRIPTION/TRANSLATION |
| 2310061J03Rik | 2310061J03RIK | 16 |  | 31.15 | 214.20 | UNKNOWN FUNCTION |
| Unc119B | AA407659 | 5 | Mus musculus unc-119 homolog B (C. elegans); expressed sequence AA407659 (AA407659), mRNA. | 31.26 | 171.79 | CILIA COMPONENT |
| Gtf2f1 | GTF2F1 | 17 | Mus musculus general transcription factor IIF, polypeptide 1 (Gtf2f1), mRNA. | 31.30 | 170.80 | TRANSCRIPTION/TRANSLATION |
| Sergef | SERGEF | 7 | Mus musculus secretion regulating guanine nucleotide exchange factor (Sergef), mRNA. | 31.31 | 201.78 | METABOLISM/MITO FXN |
| Bnip1 | BNIP1 | 17 | Mus musculus BCL2/adenovirus E1B interacting protein 1 (Bnip1), mRNA. | 31.38 | 172.98 | METABOLISM/MITO FXN |
| Npepl1 | NPEPL1 | 2 | Mus musculus aminopeptidase-like 1 (Npepl1), mRNA. | 31.42 | 219.19 | METABOLISM/MITO FXN |
| Gdap1l1 | GDAP1L1 | 2 | Mus musculus ganglioside-induced differentiation-associated protein 1-like 1 (Gdap1l1), mRNA. | 31.59 | 185.90 | UNKNOWN FUNCTION |
| 0610007J10Rik | 0610007J10RIK | not found |  | 31.81 | 161.65 | UNKNOWN GENE |
| 1810037I17Rik | 1810037I17RIK | 3 | Mus musculus RIKEN cDNA 1810037I17 gene (1810037I17Rik), mRNA. | 31.93 | 188.37 | UNKNOWN FUNCTION |
| Cdk2 | CDK2 | 10 | Mus musculus cyclin-dependent kinase 2 | 31.97 | 261.19 | CELL CYCLE |
| Gpx7 | GPX7 | 4 | Mus musculus glutathione peroxidase 7 (Gpx7), mRNA. | 31.99 | 244.82 | METABOLISM/MITO FXN |
| Stx1a | STX1A | 5 | Mus musculus syntaxin 1A (brain) (Stx1a), mRNA. | 31.99 | 164.46 | SYNAPTIC FUNCTION |
| H2-Q7 | LOC100045864 | 17 | Mus musculus histocompatibility 2, Q region locus 7 | 32.21 | 171.01 | CELL-CELL COMMUNICATION |
| Pop5 | POP5 | 5 | Mus musculus processing of precursor 5, ribonuclease P/MRP family (S. cerevisiae) (Pop5), mRNA. | 32.26 | 174.78 | METABOLISM/MITO FXN |
| Hist1h3d | HIST1H3D | 13 | Mus musculus histone cluster 1, H3d | 32.50 | 304.78 | CHROMATIN |
| Gnb2 | GNB2 | 5 | Mus musculus guanine nucleotide binding protein (G protein), beta 2 (Gnb2), mRNA. | 32.54 | 192.71 | INTRACELLULAR SIGNAL TRANSDUCTION |
| Itpkc | ITPKC | 7 | Mus musculus inositol 1,4,5-trisphosphate 3-kinase C (Itpkc), mRNA. | 32.67 | 180.50 | INTRACELLULAR SIGNAL TRANSDUCTION |
| Ltbr | LTBR | 6 | Mus musculus lymphotoxin B receptor (Ltbr), mRNA. | 32.84 | 411.03 | CELL-CELL COMMUNICATION |
| Iah1 | 4833421E05RIK | 12 | Mus musculus isoamyl acetate-hydrolyzing esterase 1 homolog (S. cerevisiae) (Iah1), mRNA. | 33.27 | 259.73 | UNKNOWN FUNCTION |
| Mex3a | MEX3A | 3 | Mus musculus mex3 homolog A (C. elegans) (Mex3a), mRNA. | 33.39 | 332.71 | TRANSCRIPTION/TRANSLATION |
| Timm50 | TIMM50 | 7 | Mus musculus translocase of inner mitochondrial membrane 50 homolog (yeast) (Timm50), nuclear gene encoding mitochondrial protein, mRNA. | 33.48 | 185.92 | METABOLISM/MITO FXN |
| Zfp260 | ZFP260 | 7 | Mus musculus zinc finger protein 260 | 33.56 | 169.46 | TRANSCRIPTION/TRANSLATION |
| Prdx4 | PRDX4 | X | Mus musculus peroxiredoxin 4 | 33.69 | 233.11 | METABOLISM/MITO FXN |
| Ict1 | ICT1 | 11 | Mus musculus immature colon carcinoma transcript 1 (Ict1), mRNA. | 33.99 | 173.47 | METABOLISM/MITO FXN |
| Bsdc1 | BSDC1 | 4 | Mus musculus BSD domain containing 1 (Bsdc1), mRNA. | 34.02 | 194.86 | UNKNOWN FUNCTION |
| Rbm11 | RBM11 | 16 | Mus musculus RNA binding motif protein 11 (Rbm11), mRNA. | 34.15 | 282.63 | TRANSCRIPTION/TRANSLATION |
| 0610011I19Rik | 0610011I19RIK | not found |  | 34.36 | 181.60 | UNKNOWN GENE |
| Blvra | BLVRA | 2 | Mus musculus biliverdin reductase A | 34.36 | 166.86 | METABOLISM/MITO FXN |
| Lrwd1 | 1200011O22RIK | 5 | Mus musculus leucine-rich repeats and WD repeat domain containing 1 (RIKEN cDNA 1200011O22 gene) | 34.58 | 197.10 | CHROMATIN |
| 2310033P09Rik | 2310033P09RIK | 11 | Mus musculus RIKEN cDNA 2310033P09 gene (2310033P09Rik), mRNA. | 34.69 | 215.50 | UNKNOWN FUNCTION |
| LOC100045300 | LOC100045300 |  | PREDICTED: Mus musculus similar to Dr1 associated protein 1 (negative cofactor 2 alpha) (LOC100045300), mRNA. | 34.93 | 163.71 | OTHER |
| Fbxo6 | FBXO6 | 4 | Mus musculus F-box protein 6 (Fbxo6), mRNA. | 35.05 | 165.75 | METABOLISM/MITO FXN |
| Rhno1 | 5930416I19RIK | 6 | Mus musculus RAD9-HUS1-RAD1 interacting nuclear orphan 1; RIKEN cDNA 5930416I19 gene (5930416I19Rik), mRNA. | 35.16 | 178.06 | CELL CYCLE |
| Idnk | 5133401N09RIK | 13 | Mus musculus idnK gluconokinase homolog (E. coli); RIKEN cDNA 5133401N09 gene, transcript variant 1, mRNA. | 35.17 | 188.12 | METABOLISM/MITO FXN |
| Commd10 | COMMD10 | 18 | Mus musculus COMM domain containing 10 (Commd10), mRNA. | 35.68 | 171.13 | UNKNOWN FUNCTION |
| Edf1 | EDF1 | 2 | Mus musculus endothelial differentiation-related factor 1 (Edf1), mRNA. | 35.95 | 175.61 | TRANSCRIPTION/TRANSLATION |
| Hmox2 | HMOX2 | 16 | Mus musculus heme oxygenase (decycling) 2 (Hmox2), mRNA. | 35.96 | 172.30 | METABOLISM/MITO FXN |
| Sertad3 | SERTAD3 | 7 | Mus musculus SERTA domain containing 3 (Sertad3), mRNA. | 35.98 | 203.66 | TRANSCRIPTION/TRANSLATION |
| Trappc2l | TRAPPC2L | 8 | Mus musculus trafficking protein particle complex 2-like (Trappc2l), mRNA. | 36.04 | 165.52 | INTRACELLULAR TRANSPORT |
| Apaf1 | APAF1 | 10 | Mus musculus apoptotic peptidase activating factor 1 (Apaf1), transcript variant 1, mRNA. | 36.16 | 188.77 | APOPTOSIS |
| Ppm1m | 2810423O19RIK | 9 | Mus musculus protein phosphatase 1M | 36.32 | 286.75 | TRANSCRIPTION/TRANSLATION |
| Rdbp | RDBP | 17 | Mus musculus RD RNA-binding protein (Rdbp), transcript variant 2, mRNA. | 36.37 | 194.32 | TRANSCRIPTION/TRANSLATION |
| Pigyl | PIGYL | 9 | Mus musculus phosphatidylinositol glycan anchor biosynthesis, class Y-like (Pigyl), mRNA. | 36.57 | 205.84 | METABOLISM/MITO FXN |
| Simc1 | 4732471D19RIK | 13 | Mus musculus SUMO-interacting motifs containing 1; RIKEN cDNA 4732471D19 gene (4732471D19Rik), mRNA. | 36.57 | 214.05 | UNKNOWN FUNCTION |
| Rit1 | RIT1 | 3 | Mus musculus Ras-like without CAAX 1 (Rit1), mRNA. | 37.35 | 170.10 | INTRACELLULAR SIGNAL TRANSDUCTION |
| H1fx | H1FX | 6 | Mus musculus H1 histone family, member X (H1fx), mRNA. | 37.60 | 232.07 | CHROMATIN |
| Akap5 | 3526401B18RIK | 12 | Mus musculus A kinase (PRKA) anchor protein 5 | 37.68 | 213.06 | METABOLISM/MITO FXN |
| Bambi-ps1 | BAMBI-PS1 | 2 | Mus musculus BMP and activin membrane-bound inhibitor, pseudogene (Xenopus laevis) | 37.83 | 284.01 | UNKNOWN FUNCTION |
| Mettl22 | BC024814 | 16 | Mus musculus methyltransferase like 22 | 37.95 | 179.83 | UNKNOWN FUNCTION |
| Acot6 | 4632408A20RIK | 12 | Mus musculus acyl-CoA thioesterase 6 | 38.03 | 200.04 | METABOLISM/MITO FXN |
| Dolpp1 | DOLPP1 | 2 | Mus musculus dolichyl pyrophosphate phosphatase 1 (Dolpp1), mRNA. | 38.10 | 232.55 | METABOLISM/MITO FXN |
| Fbxw17 | FBXW17 | 13 | Mus musculus F-box and WD-40 domain protein 17 (Fbxw17), mRNA. | 38.10 | 223.59 | METABOLISM/MITO FXN |
| Ankrd13a | ANKRD13A | 5 | Mus musculus ankyrin repeat domain 13a (Ankrd13a), mRNA. | 38.16 | 175.79 | UNKNOWN FUNCTION |
| Hist1h2bj | HIST1H2BJ | 13 | Mus musculus histone cluster 1, H2bj (Hist1h2bj), mRNA. | 38.68 | 308.87 | CHROMATIN |
| Txnrd2 | TXNRD2 | 16 | Mus musculus thioredoxin reductase 2 | 38.84 | 186.46 | METABOLISM/MITO FXN |
| Ssr4 | SSR4 | X | Mus musculus signal sequence receptor, delta | 38.91 | 169.05 | METABOLISM/MITO FXN |
| Ppp1r35 | 2010007H12RIK | 5 | Mus musculus protein phosphatase regulatory subunit 35; RIKEN cDNA 2010007H12 gene (2010007H12Rik), mRNA. | 39.47 | 198.26 | METABOLISM/MITO FXN |
| Rpl10-sl1 | LOC100043391 | 2 | ribosomal protein L10, pseudogene 1; Mus musculus ribosomal protein L10, pseudogene 1; PREDICTED: similar to QM protein (LOC100043391), mRNA. | 40.07 | 168.06 | OTHER |
| Twf2 | TWF2 | 9 | Mus musculus twinfilin, actin-binding protein, homolog 2 (Drosophila) (Twf2), mRNA. | 40.11 | 331.71 | CYTOSKELETAL/NUCLEAR ENVELOPE |
| Usp28 | USP28 | 9 | Mus musculus ubiquitin specific peptidase 28 (Usp28), mRNA. | 40.22 | 205.51 | METABOLISM/MITO FXN |
| Sat2 | SAT2 | 11 | Mus musculus spermidine/spermine N1-acetyl transferase 2 (Sat2), mRNA. XM_181304 XM_901865 XM_901869 XM_901870 | 40.28 | 180.38 | METABOLISM/MITO FXN |
| Syde1 | SYDE1 | 10 | PREDICTED: Mus musculus synapse defective 1, Rho GTPase, homolog 1 (C. elegans) (Syde1), mRNA. | 40.30 | 208.75 | INTRACELLULAR SIGNAL TRANSDUCTION |
| Prkra | PRKRA | 2 | Mus musculus protein kinase, interferon inducible double stranded RNA dependent activator (Prkra), mRNA. | 40.48 | 173.39 | TRANSCRIPTION/TRANSLATION |
| Wbp5 | WBP5 | x | Mus musculus WW domain binding protein 5 | 40.61 | 184.92 | TRANSCRIPTION/TRANSLATION |
| LOC100044101 | LOC100044101 | 16 | PREDICTED: Mus musculus similar to thioredoxin reductase 2 (LOC100044101), mRNA. | 40.66 | 204.93 | OTHER |
| Bik | BIK | 15 | Mus musculus BCL2-interacting killer (Bik), mRNA. | 40.67 | 1873.88 | APOPTOSIS |
| Wdsub1 | WDSUB1 | 2 | Mus musculus WD repeat, SAM and U-box domain containing 1 (Wdsub1), mRNA. | 40.98 | 356.44 | METABOLISM/MITO FXN |
| Tmem141 | TMEM141 | 2 | Mus musculus transmembrane protein 141 (Tmem141), mRNA. XM_979169 | 41.06 | 187.00 | UNKNOWN FUNCTION |
| Tlk1 | TLK1 | 2 | Mus musculus tousled-like kinase 1 (Tlk1), mRNA. XM_001002220 XM_982936 XM_982971 XM_983010 XM_983048 XM_983091 XM_983131 | 41.08 | 171.09 | CHROMATIN |
| Nagk | NAGK | 6 | Mus musculus N-acetylglucosamine kinase | 41.69 | 210.26 | METABOLISM/MITO FXN |
| Stxbp2 | STXBP2 | 8 | Mus musculus syntaxin binding protein 2 | 42.25 | 209.42 | INTRACELLULAR TRANSPORT |
| Dock6 | DOCK6 | 9 | Mus musculus dedicator of cytokinesis 6 (Dock6), mRNA. | 42.52 | 242.57 | INTRACELLULAR SIGNAL TRANSDUCTION |
| Mcm5 | MCM5 | 8 | Mus musculus minichromosome maintenance deficient 5, cell division cycle 46 (S. cerevisiae) (Mcm5), mRNA. | 42.74 | 212.88 | CELL CYCLE |
| Lxn | LXN | 3 | Mus musculus latexin (Lxn), mRNA. | 42.87 | 190.19 | METABOLISM/MITO FXN |
| Pygo1 | PYGO1 | 9 | Mus musculus pygopus 1 | 42.89 | 273.94 | TRANSCRIPTION/TRANSLATION |
| Dgcr6 | DGCR6 | 16 | Mus musculus DiGeorge syndrome critical region gene 6 (Dgcr6), mRNA. | 42.91 | 177.91 | CELL-CELL COMMUNICATION |
| Mvp | MVP | 7 | Mus musculus major vault protein (Mvp), mRNA. | 42.92 | 221.17 | INTRACELLULAR SIGNAL TRANSDUCTION |
| Sertad2 | SERTAD2 | 11 | Mus musculus SERTA domain containing 2 (Sertad2), transcript variant 1, mRNA. | 43.00 | 228.85 | TRANSCRIPTION/TRANSLATION |
| Zfp747 | ZFP747 | 7 | Mus musculus zinc finger protein 747 (Zfp747), mRNA. | 43.02 | 201.43 | UNKNOWN FUNCTION |
| 6330549D23Rik | 6330549D23RIK | 3 | Mus musculus RIKEN cDNA 6330549D23 gene (6330549D23Rik), non-coding RNA. | 43.40 | 314.60 | UNKNOWN FUNCTION |
| LOC100047486 | LOC100047486 |  | PREDICTED: Mus musculus hypothetical protein LOC100047486 (LOC100047486), misc RNA. | 43.80 | 186.96 | OTHER |
| 0610011F06Rik | 0610011F06RIK | 17 | Mus musculus RIKEN cDNA 0610011F06 gene (0610011F06Rik), mRNA. | 43.81 | 195.11 | UNKNOWN FUNCTION |
| Hist2h2ab | HIST2H2AB | 3 | Mus musculus histone cluster 2, H2ab (Hist2h2ab), mRNA. | 43.82 | 534.26 | CHROMATIN |
| 4932408C11Rik | 4932408C11RIK | not found |  | 44.11 | 700.80 | UNKNOWN GENE |
| Tmem22 | TMEM22 | 9 | PREDICTED: Mus musculus transmembrane protein 22 (Tmem22), mRNA. | 44.32 | 211.70 | METABOLISM/MITO FXN |
| Casp6 | CASP6 | 3 | Mus musculus caspase 6 (Casp6), mRNA. | 44.59 | 245.92 | APOPTOSIS |
| Lage3 | LAGE3 | X | Mus musculus L antigen family, member 3 (Lage3), mRNA. | 44.94 | 180.05 | UNKNOWN FUNCTION |
| Leprel4 | 1110036O03RIK | 11 | Mus musculus leprecan-like 4; RIKEN cDNA 1110036O03 gene | 45.35 | 281.28 | TRANSCRIPTION/TRANSLATION |
| Prkx | PRKX | X | Mus musculus protein kinase, X-linked (Prkx), mRNA. | 45.54 | 205.30 | METABOLISM/MITO FXN |
| Lzic | LZIC | 4 | Mus musculus leucine zipper and CTNNBIP1 domain containing (Lzic), mRNA. | 45.63 | 228.12 | UNKNOWN FUNCTION |
| Hist2h3b | HIST2H3B | 3 | Mus musculus histone cluster 2, H3b (Hist2h3b), mRNA. | 45.91 | 213.45 | CHROMATIN |
| Ccdc120 | CCDC120 | X | Mus musculus coiled-coil domain containing 120 (Ccdc120), mRNA. | 46.12 | 194.02 | UNKNOWN FUNCTION |
| Lsm10 | LSM10 | 4 | Mus musculus U7 snRNP-specific Sm-like protein LSM10 (Lsm10), mRNA. | 46.16 | 208.52 | TRANSCRIPTION/TRANSLATION |
| LINCoo493 | GM561 | 2 | Mus musculus gene model 561, (NCBI); Long intergenic non-protein-coding RNA 493 | 46.82 | 180.44 | METABOLISM/MITO FXN |
| Apitd1 | APITD1 | 4 | Mus musculus apoptosis-inducing, TAF9-like domain 1 (Apitd1), mRNA. | 47.38 | 240.87 | DNA REPAIR/REPLICATION |
| Gpr19 | GPR19 | 6 | Mus musculus G protein-coupled receptor 19 (Gpr19), mRNA. | 47.74 | 195.36 | CELL-CELL COMMUNICATION |
| Hist2h2aa2 | HIST2H2AA2 | 3 | Mus musculus histone cluster 2, H2aa2 (Hist2h2aa2), mRNA. | 47.96 | 431.97 | CHROMATIN |
| Sssca1 | SSSCA1 | 19 | Mus musculus Sjogren's syndrome/scleroderma autoantigen 1 homolog (human) (Sssca1), mRNA. | 47.99 | 183.52 | CHROMATIN |
| Ypel1 | YPEL1 | 16 | Mus musculus yippee-like 1 (Drosophila) (Ypel1), mRNA. | 48.77 | 268.11 | CYTOSKELETAL/NUCLEAR ENVELOPE |
| Spata24 | 5133400G04RIK | 18 | Mus musculus spermatogenesis associated 24; RIKEN cDNA 5133400G04 gene, transcript variant 2, mRNA. | 49.04 | 232.48 | TRANSCRIPTION/TRANSLATION |
| Nadk | BC004012 | 4 | Mus musculus NAD kinase (Nadk), mRNA. | 49.23 | 182.75 | METABOLISM/MITO FXN |
| Thoc6 | THOC6 | 17 | Mus musculus THO complex 6 homolog (Drosophila) (Thoc6), mRNA. | 50.09 | 217.98 | TRANSCRIPTION/TRANSLATION |
| rp9 | RP9 | 9 | Mus musculus retinitis pigmentosa 9 (human) (rp9), mRNA. | 50.69 | 232.26 | RETINAL DISEASE GENE |
| Samd10 | SAMD10 | 2 | Mus musculus sterile alpha motif domain containing 10 (Samd10), mRNA. | 50.71 | 198.73 | UNKNOWN FUNCTION |
| Gpn2 | GPN2 | 4 | Mus musculus GPN-loop GTPase 2 (Gpn2), mRNA. | 51.37 | 188.81 | UNKNOWN FUNCTION |
| Nme3 | NME3 | 17 | Mus musculus non-metastatic cells 3, protein expressed in (Nme3), mRNA. | 51.54 | 183.53 | METABOLISM/MITO FXN |
| 6820445E23Rik | 4930563B10RIK | 11 | Mus musculus RIKEN cDNA 6820445E23 gene | 52.67 | 193.76 | UNKNOWN FUNCTION |
| Sertad1 | SERTAD1 | 7 | Mus musculus SERTA domain containing 1 (Sertad1), mRNA. | 52.97 | 354.05 | TRANSCRIPTION/TRANSLATION |
| Btbd6 | BTBD6 | 12 | Mus musculus BTB (POZ) domain containing 6 | 53.05 | 195.78 | TRANSCRIPTION/TRANSLATION |
| 2610524H06Rik | 2610524H06RIK | 5 | Mus musculus RIKEN cDNA 2610524H06 gene (2610524H06Rik), mRNA. | 53.55 | 190.08 | UNKNOWN FUNCTION |
| 6330562C20Rik | 6330562C20RIK | 3 | Mus musculus RIKEN cDNA 6330562C20 gene | 54.29 | 319.60 | UNKNOWN FUNCTION |
| LOC100045228 | LOC100045228 |  | PREDICTED: Mus musculus similar to neuroligin 3 (LOC100045228), mRNA. | 54.91 | 214.85 | OTHER |
| Hist1h2bh | HIST1H2BH | 13 | Mus musculus histone cluster 1, H2bh (Hist1h2bh), mRNA. | 55.44 | 311.77 | CHROMATIN |
| Hist1h2be | HIST1H2BE | 13 | Mus musculus histone cluster 1, H2be (Hist1h2be), mRNA. | 56.82 | 300.76 | CHROMATIN |
| Rbmx | RBMX | X | Mus musculus RNA binding motif protein, X chromosome (Rbmx), mRNA. | 58.30 | 197.23 | TRANSCRIPTION/TRANSLATION |
| Znf512b | ZNF512B | 2 | PREDICTED: Mus musculus zinc finger protein 512B (Znf512b), mRNA. | 58.75 | 186.93 | TRANSCRIPTION/TRANSLATION |
| Tmem205 | TMEM205 | 9 | Mus musculus transmembrane protein 205 (Tmem205), mRNA. | 59.65 | 193.23 | UNKNOWN FUNCTION |
| Noc2l | NOC2L | 4 | Mus musculus nucleolar complex associated 2 homolog (S. cerevisiae) (Noc2l), mRNA. | 59.83 | 447.53 | CHROMATIN |
| Bloc1s1 | BLOC1S1 | 10 | Mus musculus biogenesis of lysosome-related organelles complex-1, subunit 1 (Bloc1s1), mRNA. | 59.92 | 191.63 | METABOLISM/MITO FXN |
| Hexim1 | HEXIM1 | 11 | Mus musculus hexamethylene bis-acetamide inducible 1 (Hexim1), mRNA. | 61.02 | 338.16 | TRANSCRIPTION/TRANSLATION |
| Nmral1 | NMRAL1 | 16 | Mus musculus NmrA-like family domain containing 1. | 61.35 | 219.04 | UNKNOWN FUNCTION |
| Fam221a | D330028D13RIK | 6 | Mus musculus family with sequence similarity 221, member A; RIKEN cDNA D330028D13 gene (D330028D13Rik), mRNA. | 62.82 | 415.07 | UNKNOWN FUNCTION |
| BC026585 | BC026585 | 1 | Mus musculus cDNA sequence BC026585 | 64.54 | 264.97 | METABOLISM/MITO FXN |
| Trp53i13 | 2410019G02RIK | 11 | Mus musculus transformation related protein 53 inducible protein 13 | 65.06 | 258.29 | UNKNOWN FUNCTION |
| Tmem120a | TMEM120A | 5 | Mus musculus transmembrane protein 120A (Tmem120a), mRNA. | 66.62 | 226.22 | UNKNOWN FUNCTION |
| Hist2h2ac | HIST2H2AC | 3 | Mus musculus histone cluster 2, H2ac (Hist2h2ac), mRNA. | 72.18 | 245.26 | CHROMATIN |
| Ruvbl2 | RUVBL2 | 7 | Mus musculus RuvB-like protein 2 (Ruvbl2), mRNA. | 73.46 | 215.10 | TRANSCRIPTION/TRANSLATION |
| Prrc1 | PRRC1 | 18 | Mus musculus proline-rich coiled-coil 1 (Prrc1), mRNA. | 73.79 | 227.10 | INTRACELLULAR SIGNAL TRANSDUCTION |
| Cryl1 | CRYL1 | 14 | Mus musculus crystallin, lambda 1 (Cryl1), mRNA. | 77.51 | 235.92 | METABOLISM/MITO FXN |
| Hist1h2bn | HIST1H2BN | 13 | Mus musculus histone cluster 1, H2bn (Hist1h2bn), mRNA. | 78.19 | 339.26 | CHROMATIN |
| Ech1 | ECH1 | 7 | Mus musculus enoyl coenzyme A hydratase 1, peroxisomal (Ech1), mRNA. | 81.65 | 211.63 | METABOLISM/MITO FXN |
| Rell1 | RELL1 | 5 | Mus musculus RELT-like 1 (Rell1), mRNA. | 83.12 | 357.88 | UNKNOWN FUNCTION |
| Rab3d | RAB3D | 9 | Mus musculus RAB3D, member RAS oncogene family (Rab3d), mRNA. | 86.41 | 354.25 | INTRACELLULAR SIGNAL TRANSDUCTION |
| Psme1 | PSME1 | 14 | Mus musculus proteasome (prosome, macropain) 28 subunit, alpha (Psme1), mRNA. | 89.86 | 216.06 | METABOLISM/MITO FXN |
| Psme2 | PSME2 | 14 | Mus musculus proteasome (prosome, macropain) 28 subunit, beta (Psme2), transcript variant 1, mRNA. | 89.97 | 334.75 | METABOLISM/MITO FXN |
| 2810405F04Rik | 2810405F04RIK | not found |  | 90.18 | 276.66 | UNKNOWN GENE |
| Xrcc6bp1 | 1110068E08RIK | 10 | Mus musculus XRCC6 binding protein 1 | 92.96 | 254.03 | DNA REPAIR/REPLICATION |
| Bax | BAX | 7 | Mus musculus Bcl2-associated X protein (Bax), mRNA. | 97.01 | 231.38 | APOPTOSIS |
| Galk1 | GALK1 | 11 | Mus musculus galactokinase 1 (Galk1), mRNA. | 101.42 | 246.28 | METABOLISM/MITO FXN |
| Jun | JUN | 4 | Mus musculus Jun oncogene (Jun), mRNA. | 103.15 | 269.87 | TRANSCRIPTION/TRANSLATION |
| Fnbp1l | FNBP1L | 3 | Mus musculus formin binding protein 1-like (Fnbp1l), mRNA. | 105.38 | 243.53 | CELL-CELL COMMUNICATION |
| Dusp23 | DUSP23 | 1 | Mus musculus dual specificity phosphatase 23 (Dusp23), mRNA. | 122.72 | 300.62 | METABOLISM/MITO FXN |
| Hist1h2bm | HIST1H2BM | 13 | Mus musculus histone cluster 1, H2bm (Hist1h2bm), mRNA. | 127.14 | 318.22 | CHROMATIN |
| Hist2h2be | HIST2H2BE | 3 | Mus musculus histone cluster 2, H2be (Hist2h2be), mRNA. | 171.41 | 402.35 | CHROMATIN |
| Rsph9 | 1700027N10RIK | 17 | Mus musculus radial spoke head 9 homolog (Chlamydomonas); RIKEN cDNA 1700027N10 gene. | 215.77 | 307.02 | CILIA COMPONENT |
| Hist1h2bk | HIST1H2BK | 13 | Mus musculus histone cluster 1, H2bk (Hist1h2bk), mRNA. | 226.66 | 323.35 | CHROMATIN |
