# Supplementary material for: Transcription Coactivators p300 and CBP Are Necessary for Photoreceptor-Specific Chromatin Organization and Gene Expression
Source: PLoS One. 2013 Jul 26;8(7):e69721. doi: 10.1371/journal.pone.0069721 (PMC3724885; doi:10.1371/journal.pone.0069721)
Supplement: Table S4 — p300/CBP dependent genes linked to retinal disease. (DOCX) [file pone.0069721.s009.docx]

| **Table S4. p300/CBP dependent genes linked to retinal disease** | |  |  |
| --- | --- | --- | --- |
| **SYMBOL** | **DEFINITION** | **%**  ***CRE NEG*** | **DOWN IN**  ***Crx^-/-^* ?** |
| Guca1b | Mus musculus guanylate cyclase activator 1B | 2.7 | Yes |
| Fscn2 | Mus musculus fascin homolog 2, actin-bundling protein, retinal | 3.1 | Yes |
| Rpgrip1 | Mus musculus retinitis pigmentosa GTPase regulator interacting protein 1 | 5.9 | Yes |
| Pde6a | Mus musculus phosphodiesterase 6A, cGMP-specific, rod, alpha | 6.1 | Yes |
| Rho | Mus musculus rhodopsin | 8.2 | Yes |
| Slc24a1 | Mus musculus solute carrier family 24 (sodium/potassium/calcium exchanger), member 1 | 8.3 | Yes |
| Cacna2d4 | Mus musculus calcium channel, voltage-dependent, alpha 2/delta subunit 4 (Cacna2d4), mRNA. | 8.8 | No |
| Cabp4 | Mus musculus calcium binding protein 4 | 10.0 | Yes |
| Grk1 | Mus musculus G protein-coupled receptor kinase 1 | 10.2 | Yes |
| Abca4 | ATP-binding cassette, sub-family A (ABC1), member 4 | 12.8 | Yes |
| Rp1l1 | Mus musculus retinitis pigmentosa 1 homolog (human)-like 1 | 13.0 | No |
| Pitpnm3 | Mus musculus PITPNM family member 3 | 13.5 | Yes |
| Pde6b | Mus musculus phosphodiesterase 6B, cGMP, rod receptor, beta polypeptide | 13.6 | Yes |
| Cnga1 | Mus musculus cyclic nucleotide gated channel alpha 1 | 13.9 | Yes |
| Rdh12 | Mus musculus retinol dehydrogenase 12 | 13.9 | Yes |
| Gnat1 | Mus musculus guanine nucleotide binding protein, alpha transducing 1 | 15.3 | Yes |
| Aipl1 | Mus musculus aryl hydrocarbon receptor-interacting protein-like 1 | 17.5 | Yes |
| Cngb1 | PREDICTED: Mus musculus cyclic nucleotide gated channel beta 1 | 18.2 | No |
| Rom1 | Mus musculus rod outer segment membrane protein 1 | 18.6 | Yes |
| Prph2 | Mus musculus peripherin 2 | 19.1 | No |
| Cacna1f | Mus musculus calcium channel, voltage-dependent, alpha 1F subunit | 20.0 | Yes |
| Impg2 | Mus musculus interphotoreceptor matrix proteoglycan 2 | 21.4 | No |
| Tulp1 | Mus musculus tubby like protein 1 | 21.4 | Yes |
| Mak | Mus musculus male germ cell-associated kinase | 22.0 | No |
| Pcdh15 | Mus musculus protocadherin 15 | 22.0 | No |
| Rgs9bp | Mus musculus regulator of G-protein signalling 9 binding protein | 22.1 | Yes |
| Rd3 | Mus musculus retinal degeneration 3 | 23.0 | No |
| Nrl | Mus musculus neural retina leucine zipper gene | 23.4 | Yes |
| Unc119 | Mus musculus unc-119 homolog (C. elegans) | 23.4 | Yes |
| Pde6g | Mus musculus phosphodiesterase 6G, cGMP-specific, rod, gamma | 24.7 | Yes |
| Rbp3 | Mus musculus retinol binding protein 3, interstitial | 25.3 | No |
| Impdh1 | Mus musculus inosine 5'-phosphate dehydrogenase 1 | 26.0 | Yes |
| Crx | Mus musculus cone-rod homeobox containing gene | 30.8 | Yes |
| Sag | Mus musculus retinal S-antigen (Arrestin-1) | 31.8 | Yes |
| Bbs7 | Mus musculus Bardet-Biedl syndrome 7 | 39.3 | No |
| Bbs5 | Mus musculus Bardet-Biedl syndrome 5 (human) (Bbs5), mRNA. | 41.1 | Yes |
| Arl6 | Mus musculus ADP-ribosylation factor-like 6 | 41.4 | No |
| Elovl4 | Mus musculus elongation of very long chain fatty acids (FEN1/Elo2, SUR4/Elo3, yeast)-like 4 | 42.9 | No |
| Myo7a | Mus musculus myosin VIIa | 43.6 | No |
| Nphp4 | Mus musculus nephronophthisis 4 (juvenile) homolog (human) | 46.0 | No |
| Htra1 | Mus musculus HtrA serine peptidase 1 | 48.9 | No |
| Pla2g5 | Mus musculus phospholipase A2, group V | 50.1 | No |
| Crb1 | Mus musculus crumbs homolog 1 (Drosophila) | 53.2 | Yes |
| Ttc8 | Mus musculus tetratricopeptide repeat domain 8 | 55.2 | No |
| Cln3 | Mus musculus ceroid lipofuscinosis, neuronal 3, juvenile (Batten, Spielmeyer-Vogt disease) | 61.6 | No |
