# Supplementary material for: Transcription Coactivators p300 and CBP Are Necessary for Photoreceptor-Specific Chromatin Organization and Gene Expression
Source: PLoS One. 2013 Jul 26;8(7):e69721. doi: 10.1371/journal.pone.0069721 (PMC3724885; doi:10.1371/journal.pone.0069721)
Supplement: Table S5 — p300/CBP dependent genes downregulated in Crx−/−. (DOCX) [file pone.0069721.s010.docx]

| **Table S5. p300/CBP dependent genes downregulated in *Crx^-/-^*** | |  |
| --- | --- | --- |
| **SYMBOL** | **DEFINITION** | **% *CRE NEG*** |
| Dhrs3 | Mus musculus dehydrogenase/reductase (SDR family) member 3 | <1.3 |
| Guca1b | Mus musculus guanylate cyclase activator 1B | 2.7 |
| Fscn2 | Mus musculus fascin homolog 2, actin-bundling protein, retinal (Strongylocentrotus purpuratus) | 3.1 |
| 2610034M16Rik | Mus musculus RIKEN cDNA 2610034M16 gene | 3.2 |
| Wisp1 | Mus musculus WNT1 inducible signaling pathway protein 1 | 4.2 |
| Esrrb | Mus musculus estrogen related receptor, beta | 5.6 |
| Rpgrip1 | Mus musculus retinitis pigmentosa GTPase regulator interacting protein 1 | 5.9 |
| Pde6a | Mus musculus phosphodiesterase 6A, cGMP-specific, rod, alpha | 6.1 |
| Kcnj14 | Mus musculus potassium inwardly-rectifying channel, subfamily J, member 14 | 7.6 |
| Aqp1 | Mus musculus aquaporin 1 | 8.0 |
| Rho | Mus musculus rhodopsin | 8.2 |
| Slco4a1 | Mus musculus solute carrier organic anion transporter family, member 4a1 | 8.2 |
| Slc24a1 | Mus musculus solute carrier family 24 (sodium/potassium/calcium exchanger), member 1 | 8.3 |
| Vtn | Mus musculus vitronectin | 8.6 |
| A930004D18Rik | Mus musculus RIKEN cDNA A930004D18 gene | 8.7 |
| Sntg2 | Mus musculus syntrophin, gamma 2 | 8.7 |
| Drd4 | Mus musculus dopamine receptor 4 | 9.1 |
| Grtp1 | Mus musculus GH regulated TBC protein 1 | 9.9 |
| Cabp4 | Mus musculus calcium binding protein 4 | 10.0 |
| Grk1 | Mus musculus G protein-coupled receptor kinase 1 | 10.2 |
| Mc1r | Mus musculus melanocortin 1 receptor | 10.6 |
| Cdr2 | Mus musculus cerebellar degeneration-related 2 | 10.8 |
| Susd3 | Mus musculus sushi domain containing 3 | 11.0 |
| Nxnl2 | Mus musculus Nucleoredoxin-like 2, RIKEN cDNA 4930519N16 gene | 11.2 |
| 4930430E16Rik | Mus musculus RIKEN cDNA 4930430E16 gene | 11.6 |
| Tnfaip3 | Mus musculus tumor necrosis factor, alpha-induced protein 3 | 11.7 |
| Samd11 | Mus musculus sterile alpha motif domain containing 11 | 11.9 |
| Hcls1 | Mus musculus hematopoietic cell specific Lyn substrate 1 | 12.0 |
| Abca4 | ATP-binding cassette, sub-family A (ABC1), member 4 | 12.8 |
| 1500016O10Rik | PREDICTED: Mus musculus RIKEN cDNA 1500016O10 gene | 13.1 |
| Gas7 | Mus musculus growth arrest specific 7 | 13.4 |
| Pitpnm3 | Mus musculus PITPNM family member 3 | 13.5 |
| Pde6b | Mus musculus phosphodiesterase 6B, cGMP, rod receptor, beta polypeptide | 13.6 |
| Mpp4 | Mus musculus membrane protein, palmitoylated 4 (MAGUK p55 subfamily member 4) | 13.8 |
| Slc16a6 | Mus musculus solute carrier family 16 (monocarboxylic acid transporters), member 6 | 13.8 |
| Cnga1 | Mus musculus cyclic nucleotide gated channel alpha 1 | 13.9 |
| Nt5e | Mus musculus 5' nucleotidase, ecto | 13.9 |
| Rdh12 | Mus musculus retinol dehydrogenase 12 | 13.9 |
| Akp2 | Mus musculus alkaline phosphatase 2, liver | 15.2 |
| Tnfsf12-tnfsf13 | Mus musculus tumor necrosis factor (ligand) superfamily, member 12-member 13 (Tnfsf12-tnfsf13) | 15.2 |
| Cds1 | Mus musculus CDP-diacylglycerol synthase 1 | 15.3 |
| Gnat1 | Mus musculus guanine nucleotide binding protein, alpha transducing 1 | 15.3 |
| Ppargc1b | Mus musculus peroxisome proliferative activated receptor, gamma, coactivator 1 beta | 16.1 |
| Kcnv2 | Mus musculus potassium channel, subfamily V, member 2 | 16.7 |
| Wdr17 | Mus musculus WD repeat domain 17 | 17.1 |
| Mylk | Mus musculus myosin, light polypeptide kinase | 17.4 |
| Aipl1 | Mus musculus Aipl1 aryl hydrocarbon receptor-interacting protein-like 1 | 17.5 |
| Rcvrn | Mus musculus recoverin (Rcvrn), mRNA. | 18.0 |
| Ppap2c | Mus musculus phosphatidic acid phosphatase type 2C | 18.1 |
| Ankrd33 | Mus musculus ankyrin repeat domain 33 | 18.2 |
| Rom1 | Mus musculus rod outer segment membrane protein 1 | 18.6 |
| Gnb1 | Mus musculus guanine nucleotide binding protein (G protein), beta 1 | 19.2 |
| Dyrk2 | Mus musculus dual-specificity tyrosine-(Y)-phosphorylation regulated kinase 2 | 19.6 |
| Cacna1f | Mus musculus calcium channel, voltage-dependent, alpha 1F subunit | 20.0 |
| Rtbdn | Mus musculus retbindin | 20.6 |
| Mdm1 | Mus musculus transformed mouse 3T3 cell double minute 1 | 20.9 |
| Tulp1 | Mus musculus tubby like protein 1 | 21.4 |
| Rgs9bp | Mus musculus regulator of G-protein signalling 9 binding protein | 22.1 |
| Slc17a7 | Mus musculus solute carrier family 17 (sodium-dependent inorganic phosphate cotransporter), member 7 | 22.1 |
| Nrl | Mus musculus neural retina leucine zipper gene | 23.4 |
| Unc119 | Mus musculus unc-119 homolog (C. elegans) | 23.4 |
| Pfkfb2 | Mus musculus 6-phosphofructo-2-kinase/fructose-2,6-biphosphatase 2 | 23.6 |
| Cpm | PREDICTED: Mus musculus carboxypeptidase M | 24.2 |
| Pde6g | Mus musculus phosphodiesterase 6G, cGMP-specific, rod, gamma | 24.7 |
| Ier3 | Mus musculus immediate early response 3 | 25.0 |
| C79127 | Mus musculus expressed sequence C79127 | 25.3 |
| Plekhf2 | Mus musculus pleckstrin homology domain containing, family F (with FYVE domain) member 2 | 25.8 |
| 1810009A15Rik | Mus musculus RIKEN cDNA 1810009A15 gene | 25.9 |
| Polg2 | Mus musculus polymerase (DNA directed), gamma 2, accessory subunit | 25.9 |
| Impdh1 | Mus musculus inosine 5'-phosphate dehydrogenase 1 | 26.0 |
| Nadkd1 | Mus musculus NAD kinase domain containing 1; RIKEN CDNA 1110020G09 GENE | 26.9 |
| Snta1 | Mus musculus syntrophin, acidic 1 | 27.0 |
| Wdr31 | Mus musculus WD repeat domain 31 | 27.0 |
| Stk35 | Mus musculus serine/threonine kinase 35 | 27.2 |
| 3110001A13Rik | Mus musculus RIKEN cDNA 3110001A13 gene | 29.1 |
| Icmt | Mus musculus isoprenylcysteine carboxyl methyltransferase | 29.3 |
| Kcnb1 | Mus musculus potassium voltage gated channel, Shab-related subfamily, member 1 | 30.2 |
| 6330442E10Rik | Mus musculus RIKEN cDNA 6330442E10 gene | 30.7 |
| Csda | Mus musculus cold shock domain protein A | 30.7 |
| Crx | Mus musculus cone-rod homeobox containing gene | 30.8 |
| Tdrd7 | Mus musculus tudor domain containing 7 | 31.6 |
| Sag | Mus musculus retinal S-antigen (Arrestin-1) | 31.8 |
| Tmem108 | Mus musculus transmembrane protein 108 | 35.3 |
| Rabgef1 | Mus musculus RAB guanine nucleotide exchange factor (GEF) 1 | 35.8 |
| Slc25a25 | Mus musculus solute carrier family 25 (mitochondrial carrier, phosphate carrier), member 25 | 36.0 |
| Ipmk | Mus musculus inositol polyphosphate multikinase | 36.9 |
| Bbs5 | Mus musculus Bardet-Biedl syndrome 5 (human) | 41.1 |
| Llgl2 | Mus musculus lethal giant larvae homolog 2 (Drosophila) | 41.6 |
| Stard7 | Mus musculus START domain containing 7 | 42.3 |
| Ap2a2 | Mus musculus adaptor protein complex AP-2, alpha 2 subunit | 43.1 |
| Stk17b | Mus musculus serine/threonine kinase 17b (apoptosis-inducing) | 43.3 |
| Slc6a6 | Mus musculus solute carrier family 6 (neurotransmitter transporter, taurine), member 6 | 43.8 |
| Galnt10 | Mus musculus UDP-N-acetyl-alpha-D-galactosamine:polypeptide N-acetylgalactosaminyltransferase 10 | 47.6 |
| Gas7 | Mus musculus growth arrest specific 7; RIKEN CDNA B230343A10 GENE | 47.9 |
| Ldha | Mus musculus lactate dehydrogenase A | 48.3 |
| Mbnl1 | Mus musculus muscleblind-like 1 (Drosophila) | 48.6 |
| D6Wsu176e | Mus musculus FAM3C (D6Wsu176e) | 50.3 |
| Cst3 | Mus musculus cystatin C | 50.5 |
| Adipor1 | Mus musculus adiponectin receptor 1 | 52.6 |
| Crb1 | Mus musculus crumbs homolog 1 (Drosophila) | 53.2 |
| Osgep | Mus musculus O-sialoglycoprotein endopeptidase | 61.4 |
