# Supplementary material for: Transcription Coactivators p300 and CBP Are Necessary for Photoreceptor-Specific Chromatin Organization and Gene Expression
Source: PLoS One. 2013 Jul 26;8(7):e69721. doi: 10.1371/journal.pone.0069721 (PMC3724885; doi:10.1371/journal.pone.0069721)
Supplement: Table S6 — ERG Test Parameters. (DOCX) [file pone.0069721.s011.docx]

**Table S6. ERG Test Parameters**

| **DARK-ADAPTED PROGRAM:** | **INTENSITY log[cdS/M^2^]** | **#TRIALS IN AVG** | **INTERVAL BETWEEN FLASHES** | **PAUSE AFTER STEP** |
| --- | --- | --- | --- | --- |
| Step 1 | -4.60 | 10 | 3.2 sec |  |
| Step 2 | -3.62 | 10 | 3.2 sec |  |
| Step 3 | -2.40 | 10 | 5.3 sec |  |
| Step 4 | -1.60 | 8 | 8.3 sec |  |
| Step 5 | -1.20 | 8 | 12.6 sec | 30 sec |
| Step 6 | -0.60 | 6 | 15.3 sec | 1 min |
| Step 7 | -0.01 | 6 | 20.3 sec | 1 min |
| Step 8 | 0.40 | 5 | 30.3 sec | 1.5 min |
| Step 9 | 0.89 | 5 | 32.8 sec |  |
| **LIGHT-ADAPTED PROGRAM:** |  |  |  | 10 min  light adapt |
| Step 1 | -0.01 | 10 | 1.33 sec |  |
| Step 2 | 0.40 | 10 | 1.83 sec |  |
| Step 3 | 0.89 | 8 | 2.03 sec |  |
| Step 4 | 1.41 | 6 | 2.23 sec |  |
| Step 5 | 1.90 | 6 | 2.83 sec |  |
| Step 6 | 2.39 | 5 | 3.03 sec |  |
| Step 7 | 2.67 | 5 | 3.33 sec |  |
