# Supplementary material for: Transcription Coactivators p300 and CBP Are Necessary for Photoreceptor-Specific Chromatin Organization and Gene Expression
Source: PLoS One. 2013 Jul 26;8(7):e69721. doi: 10.1371/journal.pone.0069721 (PMC3724885; doi:10.1371/journal.pone.0069721)
Supplement: Table S7 — Antibodies. (DOCX) [file pone.0069721.s012.docx]

**Table S7. Antibodies**

| **NAME** | **SOURCE** | **EPITOPE** | | **TYPE** | **SPECIES** | **USED AT** |
| --- | --- | --- | --- | --- | --- | --- |
| acetyl-Histone H3 (AcH3) | Millipore (06-599) | | Histone H3, Acetylated | polyclonal | rabbit | IHC 1:100 WB1:3000 |
| acetyl-Histone H4 | Millipore (06-598) | | Histone H4, Acetylated | polyclonal | Rabbit | IHC1:1000 WB1:3000 |
| b actin (clone AC-15) | Sigma (A1978) | | Actin N-term (20-33) (reacts against all isoforms) | mono IgG1 | mouse ascites | WB 1:3000 |
| Blue (S)-Opsin | Millipore (AB5407) | | blue opsin | polyclonal | rabbit | IHC 1:1000 |
| Calbindin D-28K | Sigma (C 7354) | | rat calbindin D-28K | polyclonal | rabbit | IHC 1:200 |
| Cbp (Kat3A) | Abcam (ab2832) | | aa 162-176 of  human Cbp | polyclonal | rabbit | IHC 1:500 |
| Chicken anti-GFP | Abcam (ab13970) | | GFP | polyclonal | chicken | IHC 1:1000 |
| Cone arrestin | Millipore (AB15282) | | cone arrestin | polyclonal | rabbit | IHC 1:1000 |
| Cone Transducin (Gat2) | Santa Cruz (sc-390) | | cone transducin | polyclonal | rabbit | IHC 1:200 |
| Crumbs | Jarema Malicki, Tufts | | crumbs | polyclonal | rabbit | IHC 1:200 |
| Crx (H00001406-M02) | Abnova (MO2) | | GST-Crx (aa 1-96) | monoclonal | mouse | IHC 1:200 |
| C-terminal binding protein 1 (CtBP1) | BD Transduction Labs (612042) | | C-term binding protein 1 | mono IgG1 | mouse | IHC 1:100 |
| GAPDH | Sigma (G9545) | | GAPDH | polyclonal | rabbit | WB 1:3000 |
| GFP (mAb3E6) | Invitrogen (A11120) | | GFP | mono IgG2a | mouse | IHC 1:1000 |
| Glial Fibrillary Acidic Protein (GFAP) | DAKO | | GFAP | polyclonal | rabbit | IHC 1:400 |
| Glutamine Synthetase | BD Transduction (610518) | | Glutamine Synth. (Muller cells) | mono IgG2a | Mouse | IHC 1:100 |
| Hes1 | Nadean Brown | | Hes1 | polyclonal | rabbit | IHC 1:250 |
| Histone H2B | Millipore (05-1352) | | Histone H2B | monoclonal | mouse | WB 1:3000 |
| Histone H3 | Millipore (05-499) | | Histone H3 | mono IgG1k | mouse | WB 1:3000 |
| Histone H3 (tri-methyl K9) | Abcam (8898) | | H3 K9-me3 | polyclonal | rabbit | IHC 1:1000 |
| Histone H3K27me3 | Millipore (07-449) | | H3H27me3 | polyclonal | rabbit | IHC 1:250 |
| Histone H3K4me3 | Millipore (ab1012) | | Histone H3 trimethyl K4 | polyclonal | rabbit | IHC 1:1000 |
| Histone H4 acetyl-K 5/8/12/16 | ThermoPierce/VWR (PA1-84526) | | Histone H4, Acetylated | polyclonal | rabbit | IHC 1:100 |
| Ki67 | BD Pharmingen (550609) | | Human Ki-67 | mono IgG1 | mouse | IHC 1:400 |
| Nestin | Millipore (MAB353) | | nestin | mono IgG1 | mouse | IHC 1:1000 |
| NeuN | Abcam (AB 77315) | | NeuN |  | mouse | IHC 1:100 |
| NeuroD (N19) | Santa Cruz(sc-1084) | | none | polyclonal | goat | IHC 0.2 γ/λ |
| Neurofilament 200  (N-52) | Sigma (N 0142) | | neurofilament 200 | mono IgG1 | mouse ascites | IHC 1:800 |
| p300-CT (RW128) | Millipore 05-257 | | human C-terminal p300 | monoclonal | mouse | IHC 1:300 |
| Pax6 | Covance (PRB-278P) | | Pax6 | polyclonal | rabbit | IHC 1:200 |
| phospho Histone H3 (Ser10) | Millipore (06-570) | | human phospho- histone H3 Ser10 | polyclonal | Rabbit | IHC 1:1000 |
| phospho-histone H2A.X (JBW301) | Millipore (05-636) | | ser139-PO4 | mono IgG1 | mouse | IHC 1:1000 |
| PKCα | Sigma (P 5704) | | protein kinase Cα aa 296-317 | mono IgG2a | mouse ascites | IHC 1:1000 |
| PSD95 | Abcam (ab18258) | | PSD95 (mouse peptide-KLH) | polyclonal | rabbit | IHC 1:500 |
| Recoverin | Abcam (ab71624) | | ecoverin C-term | polyclonal | rabbit | IHC 1:50 |
| Red/Green (M)-Opsin | Millipore (AB5405) | | rHuman R/G opsin | polyclonal | rabbit | IHC 1:1000 |
| Retinoblastoma (C-15) | Santa Cruz (sc-50) | | pan Rb | polyclonal | rabbit | IHC 1:400 |
| Rhodopsin clone RET-P1 | Sigma (O4886) | | Rhodopsin N-term (aa 4-10) | monoclonal (IgG1) | mouse ascites | IHC 1:400 |
| Ribeye (CtBP2) | BD Transduction Labs (612044) | | C-term binding protein 2 | mono IgG1 | mouse | IHC 1:2000 |
| Rod transducin (Gat1) | Santa Cruz (sc-389) | | rod transducin | polyclonal | rabbit | IHC 1:100 |
| Sox2 (C70B1) | Cell Signaling (3728) | | Sox2 | mono Rb-IgG | rabbit | IHC 1:500 |
| Syntaxin (HPC-1) | Sigma (S 0664) | | syntaxin | mono IgG1 | mouse ascites | IHC 1:100 |
| TuJ1 | Chemicon (CBL412) | | gamma tubulin-3 | mono IgG1 | mouse | IHC 1:1000 |
| VGLUT1 (vesicular glutamate transporter 1) | Millipore (ab 5905) | | VGLUT1 | polyclonal | guinea pig | IHC 1:1000 |
| ZO1 tight junction protein | Abcam (ab59720) | | ZO1 aa 432-1150 | polyclonal | rabbit | IHC 1:100 |
